# Identification and validation of postpartum depression subtypes: a population-based cohort study

**DOI:** 10.1016/j.eclinm.2025.103540

**Published:** 2025-10-09

**Authors:** Anna E. Bauer, Piotr Jaholkowski, Yi Lu, Jerry Guintivano, Laurie J. Hannigan, Elizabeth C. Corfield, Martin Tesli, Ole A. Andreassen, Yun Li, Ted Reichborn-Kjennerud, Patrick F. Sullivan, Samantha Meltzer-Brody, Alexandra Havdahl, Helga Ask

**Affiliations:** a Department of Psychiatry, University of North Carolina at Chapel Hill, Chapel Hill, NC, USA; b Department of Molecular Genetics and Microbiology, Duke University, Durham, NC, USA; c Institute of Psychiatry and Neurology, University of Oslo, Oslo, Norway; d Department of Medical Epidemiology and Biostatistics, Karolinska Institutet, Stockholm, Sweden; e Nic Waals Institute, Lovisenberg Diaconal Hospital, Oslo, Norway; f PsychGen Centre for Genetic Epidemiology and Mental Health, Norwegian Institute of Public Health, Oslo, Norway; g Population Health Sciences, Bristol Medical School, University of Bristol, Bristol, UK; h MRC Integrative Epidemiology Unit, Population Health Sciences, Bristol Medical School, University of Bristol, Bristol, UK; i Department of Mental Health and Suicide, Norwegian Institute of Public Health, Oslo, Norway; j Department of Psychiatry, Østfold Hospital, Østfold, Norway; k Centre for Precision Psychiatry, Division of Mental Health and Addiction, University of Oslo and Oslo University Hospital, Oslo, Norway; l Department of Genetics, University of North Carolina at Chapel Hill, Chapel Hill, NC, USA; m Department of Biostatistics, University of North Carolina at Chapel Hill, Chapel Hill, NC, USA; n Department of Genetics, Environment, and Mental Health, Norwegian Institute of Public Health, Oslo, Norway; o Institute of Clinical Medicine, University of Oslo, Oslo, Norway; p PROMENTA Research Centre, Department of Psychology, University of Oslo, Oslo, Norway

**Keywords:** Postpartum depression, Perinatal psychiatry, Cluster analysis, Polygenic risk, The Norwegian mother, Father and child cohort study

## Abstract

**Background:**

Postpartum depression (PPD) affects 18% of postpartum women globally. PPD is a heterogeneous condition with diverse presentations, which may differ in underlying etiology, potential outcomes, and optimal treatment. This study aimed to identify and validate PPD subtypes using comprehensive phenotypic and genetic data from a large, nationwide cohort.

**Methods:**

In this population-based cohort study, we used data from the Norwegian Mother, Father, and Child Cohort Study and Medical Birth Registry of Norway (MoBa), and applied unsupervised clustering (Uniform Manifold Approximation and Projection [UMAP] + Density-Based Spatial Clustering of Applications with Noise [DBSCAN]) to identify PPD subtypes among 7859 women with PPD (training set n = 5239). All pregnant women in Norway were eligible to enroll in MoBa between Jun 1, 1999 and Dec 31, 2008. Among women with their first recorded pregnancies in MoBa, they were considered to have clinically significant PPD if they scored ≥8 on the 6-item version of the Edinburgh Postnatal Depression Scale (EPDS) (equivalent to ≥11 on the full EPDS) administered at six months after birth. Input variables included psychiatric symptom severity, psychiatric history, trauma history, pain, and substance use. We then characterized clusters and tested associations with perinatal-relevant auxiliary variables and polygenic scores (PGS) for eight psychiatric and neurodevelopmental conditions. Findings were validated in a reserved test set (n = 2620).

**Findings:**

Nine clusters were identified in the training set (n = 5239), with seven replicating in the independent test set (n = 2620). Clusters were differentiated by symptom severity, onset timing, trauma history, and pain during pregnancy. In the training set, the cluster with the most severe symptoms was characterized by depression + trauma and comprised 28% [1483/5239] of training sample. This cluster showed marked socioeconomic adversity and elevated genetic risk for psychiatric conditions compared to other clusters (ADHD PGS OR 1.19, 95% CI 1.12–1.28; major depression PGS OR 1.15, 95% CI 1.07–1.23; PTSD PGS OR 1.09, 95% CI 1.02–1.17). In the test set, this cluster also comprised 28% [729/2620] and showed consistent associations with ADHD (OR 1.23, 95% CI 1.12–1.35), major depression (OR 1.09, 95% CI 0.99–1.20), and PTSD (OR 1.17, 95% CI 1.06–1.29). Conversely, the mild PPD cluster (10% [498/5239] of training sample) showed a protective profile (ADHD PGS OR 0.81, 95% CI 0.73–0.90; major depression PGS OR 0.85, 95% CI 0.77–0.94). In the test set, it represented 10% (263/2620), with consistent protective associations for ADHD (OR 0.84, 95% CI 0.72–0.96) and major depression (OR 0.77, 95% CI 0.67–0.89). Two early-onset clusters demonstrated distinct profiles despite similar symptom severity. The cluster characterized as early-onset PPD + pain (6% of training set [310/5239] and 7% of test set [178/2620]) had the highest prevalence of somatic conditions, including migraines (19% [58/310] training; 15% [26/178] test), nausea (47% [144/310] training; 53% [94/178] test), prenatal hospitalization (41% [128/310] training; 35% [62/178] test), and birth by caesarean section (10% [31/310] training; 8% [14/178] test), whereas the cluster characterized as early-onset PPD + anger (6% [307/5239] of training sample; 6% [158/2620] of test sample) showed many fewer physical health burdens. The clusters in the training and testing set had moderate to high concordance (ARI = 0.76 [95% CI 0.74, 0.77], FMI = 0.79 [95% CI 0.77, 0.81]).

**Interpretation:**

This study identifies clinically relevant PPD subtypes with distinct genetic and obstetric profiles, highlighting the importance of trauma-informed care, pain management, and holistic obstetric approaches in PPD prevention and treatment. Limitations of this study include reliance on self-reported data, lack of diversity in the genetically homogeneous Norwegian sample, and genetic analyses subject to power constraints, which may limit generalizability and reduce precision of results. Despite these limitations, identification of reproducible subtypes with different risk factors and outcomes provides a framework for developing targeted interventions and advancing precision psychiatry in maternal mental health.

**Funding:**

10.13039/100000025National Institute of Mental Health; 10.13039/100000071National Institute of Child Health and Human Development; MRC Integrative Epidemiology Unit, 10.13039/501100000883University of Bristol; 10.13039/501100000781European Research Council; 10.13039/100010665Marie Skłodowska-Curie Actions; 10.13039/501100006095South-Eastern Norway Regional Health Authority; 10.13039/501100005416Research Council of Norway; 10.13039/501100004785NordForsk; European Union Horizon 2020; Novo Nordisk Foundation.

**Research in context**

**Evidence before this study**

We searched PubMed and Google Scholar without language or date restrictions, using combinations of Medical Subject Headings (MeSH) and free-text terms related to postpartum depression (PPD) [*postpartum depression, postnatal depression, puerperal depression*], clinical heterogeneity [*cluster, subtype, latent class, trajectory*], physical-health correlates [*pelvic pain, caesarean section, migraine, gestational diabetes*, etc.], and psychiatric comorbidity [*major depressive disorder, bipolar disorder, schizophrenia, post traumatic stress disorder*]. Reference lists of all eligible papers and relevant reviews were hand-searched to identify any additional relevant research. To capture genetic evidence, we queried the NHGRI-EBI GWAS Catalog (search date Sep 26, 2024) for genome-wide association studies of PPD and 70 obstetric, gynecologic, endocrine, pain, and psychiatric phenotypes (full term list in Supplementary Materials). We focused on original human studies that enrolled ≥100 mothers assessed for PPD within 12 months postpartum and reported between-group comparisons of symptom patterns, timing of onset, reproductive or psychiatric outcomes, or genetic risk. We excluded animal studies, case reports, and narrative reviews. Our search identified studies that suggested possible subgroups within PPD, primarily differentiated by symptom severity and timing of onset. Recent genetic research, including the first large-scale genome-wide association study of PPD, indicated both shared genetic architecture with major depression and unique components specific to PPD. Previous investigations of PPD heterogeneity relied mainly on model-based dimension reduction techniques and did not incorporate genetic data. Prior work did not jointly examine data-driven PPD subtypes with broad reproductive health outcomes, obstetric pain phenotypes, or polygenic scores across multiple psychiatric disorders, leaving gaps in understanding how physical health and genetic liability interact to shape postpartum depression heterogeneity.

**Added value of this study**

To our knowledge, this study represents the first application of unsupervised clustering to identify PPD subtypes using comprehensive longitudinal phenotypic and genetic data. We identified nine reproducible clusters, validating previously known subgroups while revealing novel differentiating characteristics, particularly the role of trauma history and pain during pregnancy. Our findings demonstrate that PPD subtypes have distinct genetic and obstetric profiles, with some clusters showing stronger associations with polygenic scores for various psychiatric conditions. The study also highlighted the unexpected prominence of physical health factors, particularly pain, in differentiating PPD subtypes.

**Implications of all the available evidence**

The identification of distinct PPD subtypes, characterized by different combinations of psychiatric, obstetric, and genetic factors, suggests the need for more personalized treatment approaches. Our findings emphasize the importance of trauma-informed care and pain management during pregnancy, expanding beyond traditional psychiatric interventions. The regular contact between healthcare providers and women during pregnancy provides an ideal opportunity to implement targeted interventions based on subtype-specific risk factors. The identification of potential non-invasive indicators of hormonal sensitivity could enhance patient stratification in clinical trials and guide the application of newly approved PPD-specific treatments. Interpretation of these findings should be tempered by key limitations including reliance on self-reported data, a largely Norwegian/European ancestry cohort, and exploratory polygenic analyses with varying power, which may restrict generalizability and could either dilute or exaggerate some subtype-outcome associations. Despite these limitations, our results provide a framework for advancing precision psychiatry in maternal mental health, with the goal of ultimately improving outcomes for mothers and their families.

## Introduction

Postpartum depression (PPD) affects 18% of postpartum women globally, causing significant morbidity and mortality.^1^ Symptoms may begin during pregnancy or postpartum, sometimes persisting beyond the first year.^2^ While the Diagnostic and Statistical Manual (DSM-5) classifies PPD as a subtype of major depressive disorder (MDD), with onset during pregnancy or within four weeks postpartum,^3^ PPD is frequently considered part of the broader spectrum of Perinatal Mood and Anxiety Disorders (PMADs) for which mood and anxiety symptoms present within the first postpartum year.^4^ Identifying and effectively treating PPD is crucial not only for maternal well-being but also due to its profound implications for child development. Maternal depression has been linked to impaired mother-infant bonding, delayed cognitive and emotional development, and increased long-term behavioral problems in children.^5^^,^^6^ Furthermore, emerging evidence suggests that the timing, duration, and specific symptom profiles of maternal depression may differentially affect child outcomes,^7^^,^^8^ highlighting the importance of characterizing PPD heterogeneity from both maternal and child health perspectives.

Contemporary reviews emphasize that PMADs emerge from complex bio-psycho-social factors, with genetic vulnerability, hormonal fluctuations, psychological stressors, and social support all playing important roles.^9^, ^10^, ^11^ It is likely that heterogeneous subgroups exist within this broad phenotype, entailing distinct etiological mechanisms, outcomes and optimal treatment.

Prior studies of PPD heterogeneity have suggested possible subgroups, but their etiological and clinical significance remains unclear. Factors previously linked to etiological variations in depressive disorders (e.g., comorbid anxiety and hypomania) might be especially relevant in the postpartum period. Nearly half of women with PPD experience anxiety symptoms.^12^ Hypomania prevalence is high (10–20%) after delivery^13^ and bipolar disorder more often occurs following PPD than non-perinatal MDD.^14^ Mood changes driven by neuroendocrine hormone shifts^15^ and adverse life events^16^ are also linked to depression etiology, with the postpartum period being particularly susceptible due to rapid hormonal changes and childbirth-related stressors. Other factors suggested to discriminate distinct PPD subgroups include symptom severity,^17^ timing of onset,^18^^,^^19^ and suicidal ideation.^17^^,^^20^ Longitudinal trajectory studies have even suggested that perinatal predictors may differentiate patterns of maternal depressive symptoms more than a decade beyond the time of childbirth.^21^

Genetic research on PPD is also gaining traction, offering insights into biological mechanisms. The first large-scale genome-wide association study (GWAS) of PPD (18,770 cases),^22^ albeit not identifying any genome-wide significant variants, suggested a potential role for neurons involving gamma-aminobutyric acid (GABA) and revealed high genetic correlation between PPD and MDD (r_g_ = 0.95). Importantly, cell type enrichment analyses also suggested unique genetic components of PPD distinct from broader MDD. Investigating subgroups within PPD may help clarify its biological basis and mechanisms underlying other depressive disorders.

Previous studies on PPD heterogeneity have not incorporated genetic data and have predominantly used model-based dimension reduction techniques (i.e., exploratory factor analysis [EFA] and latent class analysis [LCA]).^2^^,^^11^ Non-model-based unsupervised clustering methods^2^^,^^11^^,^^19^ can handle large, complex datasets with mixed data types and do not presume a predefined structure, enabling discovery of unknown patterns or hidden subgroups.^23^

Our study extends previous work by integrating PPD-specific features, genetic data, and unsupervised clustering on rich longitudinal cohort data to uncover novel PPD subgroups. We cluster based on a range of known clinical characteristics and MDD risk factors (e.g., psychiatric history, comorbidities, adverse life events) combined with features unique to PPD (e.g., pregnancy and birth-related factors, maternal stressors). These variables were selected based on three criteria: (1) demonstrated associations with PPD in prior literature, (2) potential ability to discriminate between etiologically distinct subtypes, and (3) feasibility of assessment in routine clinical settings to maximize translational potential. We then characterize the subgroups by relevant auxiliary variables and quantify cluster associations with genetic liability for psychiatric traits.

## Methods

### Data

The Norwegian Mother, Father, and Child Cohort Study (MoBa) is a population-based study comprising approximately 114,500 children, 95,200 mothers, and 75,200 fathers, linked to data from the national Medical Birth Registry of Norway (MBRN). From Jun 1, 1999 to Dec 31, 2008, participants were recruited at their first ultrasound (gestational week 17–19) across Norway.^24^^,^^25^ All pregnant women were eligible and 41% consented to participation. All participants identified as the pregnant mother of the index child when enrolled. Sex was recorded as female at baseline; gender identity was not assessed. Participants completed questionnaires at three pregnancy timepoints and 6-months postpartum. Blood samples were collected from both parents at the first timepoint in pregnancy, and maternal and umbilical cord blood were collected at childbirth.^26^ Details of MoBa genotyping, quality-control, and imputation are previously described.^27^

**Fig. 1:**
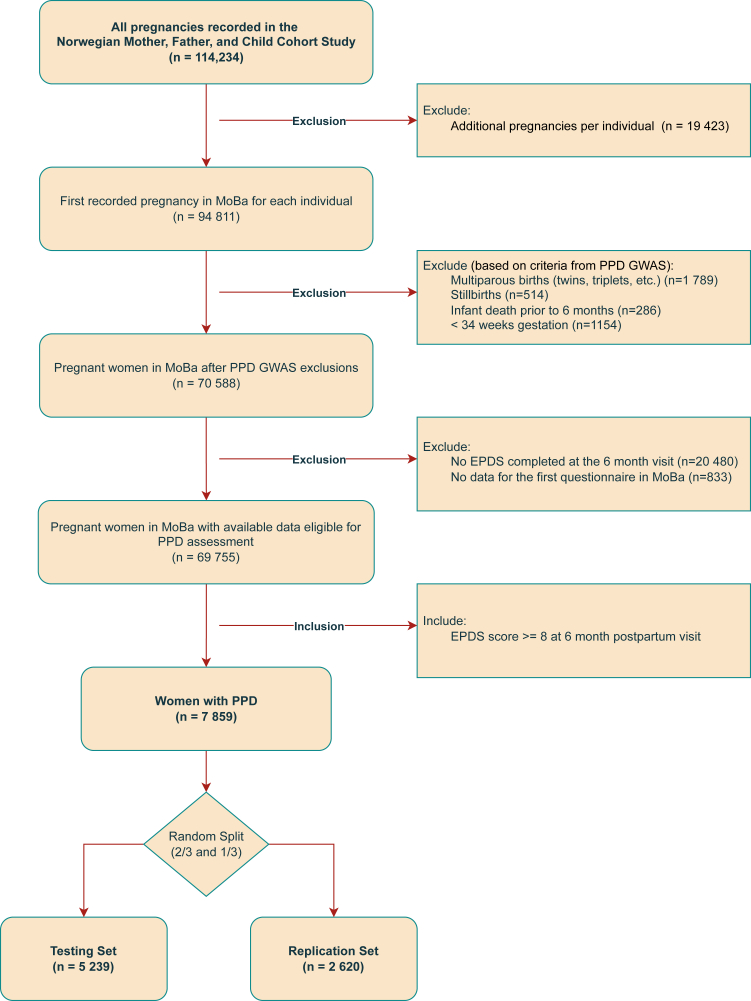
**Flow diagram of the study sample.** Flow diagram of the progress through the phases of enrollment into MoBa, exclusion criteria, selection as a PPD case for the current study, and randomization into testing and replication sets. *Abbreviations:* MoBa, Norwegian Mother, Father, and Child Cohort Study; PPD, postpartum depression; EPDS, Edinburgh Postnatal Depression Scale.

The study sample includes women with PPD, defined based on their score on the 6-item version of the Edinburgh Postnatal Depression Scale (EPDS) from the 6-month questionnaire. Although mood symptoms were assessed at several timepoints with other instruments (e.g., Hopkins Symptoms Checklist), the EPDS was only administered at the 6-month postpartum timepoint. PPD was defined as a score of ≥8, equivalent to ≥11 on the full EPDS,^28^ a definition used in prior MoBa papers,^22^ and the field.^28^ If a mother had PPD in multiple pregnancies, only the first recorded pregnancy was included. Participants were excluded if they did not complete the baseline questionnaire; had a multiple (e.g., twins), preterm (<34 weeks gestation), or stillbirth; or if their infant died prior to the 6-month assessment. The complete study sample included all women in the MoBa cohort meeting our inclusion criteria. The final complete sample included 7859 eligible women, which prior to any analysis, was randomly split into a training set for primary clustering analysis and a test set reserved for later independent replication in untouched data. Fig. 1 shows a flow diagram of selection criteria into the study.

MoBa was approved by the Regional Committee for Medical and Health Research Ethics in Norway. All women provided written informed consent at enrollment. The present secondary analyses were additionally approved by the institutional review boards at the University of North Carolina at Chapel Hill (19-1165) and the Norwegian Institute of Public Health via the Regional Committees for Medical and Health Research Ethics (14140 and 2016/1702). This study is reported in accordance with the Strengthening the Reporting of Observational Studies in Epidemiology (STROBE) and its extension, STrengthening the REporting of Genetic Association Studies (STREGA).

### Input variables

We selected (self-reported) input variables based on their ability to discriminate PPD aetiologies and clinical trajectories in prior studies, as well as their ability to be evaluated in clinical care settings. These included measures of PPD symptom severity [*EPDS, medication use for depression*]; anxiety and anger during pregnancy and postpartum [*medication use for anxiety, Hopkins Symptoms Checklist anxiety subscale, Differential Emotions Scale anger subscale*]; psychiatric history [*pre-pregnancy history/symptoms of depression, anxiety, or eating disorder*]; symptom onset [*depressive symptoms in early or late pregnancy, or early postpartum*]; substance use during pregnancy [*alcohol consumption and smoking*]; trauma history [*number of adverse life events experienced in the year prior to pregnancy, lifetime history of physical and sexual abuse*]; and physical impairment in late pregnancy and the early postpartum [*third trimester severe pelvic pain, postpartum length of hospital stay, physical health at 6 months*]. Further details are provided in Table S1.

### Characterization variables

Auxiliary characterization variables were chosen for their relevance to the perinatal period and previous associations with PPD, including demographic factors, social support, medical comorbidities, pregnancy and birth factors, infant complications, and reproductive factors. See full list in Table S2.

### Polygenic scores

We calculated polygenic scores (PGS) for psychiatric and neurodevelopmental conditions using PRSice2^29^ (Tables S3–S5) as weighted sums of risk alleles per individual, based on effect sizes from the latest published GWAS of anorexia nervosa,^30^ attention deficit hyperactivity disorder (ADHD),^31^ anxiety disorder,^32^ bipolar disorder,^33^ MDD,^34^ obsessive compulsive disorder (OCD),^35^ post-traumatic stress disorder (PTSD),^36^ and schizophrenia^37^ (Table S6). For each condition, 10 PGS were generated using single nucleotide polymorphisms (SNPs) at increasing p-value thresholds of association: p < 5.0 × 10^−8^ (the field standard for genome-wide significance),^38^ 5.0 × 10^−7^, 5.0 × 10^−6^, 5.0 × 10^−5^, 0.001, 0.005, 0.01, 0.1, 0.5, 1 (all SNPS). As a preprocessing step to account for potential confounding due to population stratification and technical batch effects, we residualised the PGS by regressing on genotype batch and the first 10 genetic principal components. We used linear regression models with normally distributed random errors and identity link function at all thresholds for each of the eight conditions. Following Coombes et al., we derived a single polygenic predictor for each condition by extracting the first principal component (PC) across all thresholds, which explained 40–67% of the variance across the different PGS thresholds for each condition, maximizing predictive power while minimizing overfitting.^39^ For the PC analysis, we used unrotated PCs, as our goal was to maximize the variance explained and create one composite polygenic score that captures the maximum shared genetic signal across thresholds. Thus, we compute eight different composite polygenic scores, one for each trait. Plots of proportion of variance explained by each of the 10 PCs and of PC loadings by p-value threshold for each of the eight conditions are presented in the Supplementary Methods.

### Statistical analysis

We performed all analyses in R (v4.2.1). Raw survey data from the full MoBa cohort were processed using an internally developed and publicly available *phenotools* package^40^ to ensure systematic coding of survey instruments and reproducibility. Missing data (n = 2720/69,755; 3.9%) were imputed with random forest nonparametric imputation using the *missForest* package with default parameters in R. The imputation model was applied to all first pregnancies in the full MoBa cohort (n = 69,755) and included all variables used for clustering and characterization as predictors (Tables S1 and S2). The original data and final imputed values for missing data points were extracted together as a complete dataset. Eligible PPD cases were then identified, and following standard machine learning practices for model development and validation,^41^ we randomly split eligible PPD cases into a training set (2/3, n = 5239) and an independent test set reserved for projection and validation (1/3, n = 2620). Both sets were derived from the same overall MoBa cohort but included different individuals, with random assignment to either the training or test set.

While k-fold cross-validation is often more efficient for model performance estimation, we opted for a split-sample validation approach to enable a true independent validation of our clustering solution. This approach allowed us to test whether the identified clusters truly represent stable, reproducible subtypes that would be found in a new sample, rather than potentially overfitting to the specific characteristics of our training data. Additionally, having a separate test set allowed for a more straightforward assessment of the reproducibility of specific cluster-variable associations, which is central to our research aims.

For unsupervised clustering, we used Uniform Manifold Approximation and Projection (UMAP) with default parameters to reduce dimensionality and project in a two-dimensional layout. Data were scaled to accommodate zero inflation and mixed data types. We applied the Density-Based Spatial Clustering of Applications with Noise (DBSCAN) to identify clusters of varying shape and size.^42^

To identify features that differentiate and characterize clusters, we visualized clusters with radar plots and computed summary statistics of auxiliary variables by cluster. Statistical comparisons were conducted using the R package *gtsummary*. For continuous variables, the non-parametric Kruskal–Wallis rank-sum test was used by default, providing robustness to non-normal distributions when comparing across categorical groups. For categorical variables, Pearson's Chi-square tests were used unless expected cell counts fell below five, in which case Fisher's exact tests were automatically applied. After testing linearity assumptions (see Box–Tidwell statistics in Table S9), separate logistic regression models were run including each of the eight PGS as exposure variables with each cluster as outcome (1 = in the specific cluster, 0 = in any other cluster). Models were adjusted for the first 10 genetic principal components, genotyping batch, and imputation batch. We chose to analyze clusters as separate binary outcomes due to interpretability and clinical relevance; specifically, no clear natural reference category emerged from the clustering approach, and it may be more clinically meaningful to evaluate whether an individual fits into a given symptom profile rather than comparing the likelihood of membership between different profiles. Following reviewer suggestions, multinomial logistic regression analyses were performed to account for the multi-category nature of cluster membership (using the lowest and highest risk groups as alternative reference categories). These results remained consistent with our primary analysis approach, but the binary approach provided more straightforward interpretations of the specific associations with each individual cluster (Supplementary Methods; Tables S11 and S12). Corrections for multiple comparisons were implemented using the False Discovery Rate (FDR)^43^ for three groups of related tests (i.e., associations between clusters and 1: input variables, 2: auxiliary variables, and 3: PGS).

### Test set validation

We projected the independent test data onto the same manifold (i.e., high-dimensional space) used for the training data. We applied DBSCAN with identical parameters to the test set,^44^ noting associations replicated if they met an FDR-corrected q-value <0.05 in both sets. Nearest neighbor linking between sets allowed us to compute the Adjusted Rand Index (ARI)^45^ and Fowlkes-Mallows Index (FMI)^46^ to empirically evaluate concordance between training and test clusters.

### Role of the funding source

The funder of the study had no role in study design, data collection, data analysis, data interpretation, or writing of the report. Study authors (AEB, LJH, EEC, AH, HA) had full access to the data in the study and all authors had final responsibility for the decision to submit for publication.

## Results

### Sample characteristics

Participant characteristics were similar in the training (n = 5239) and test (n = 2620) sets (Fig. 1), with the only statistically significant differences observed between the sets for marital/partner status (p = 0.041), confirming the success of our random splitting procedure (Table 1). Average age at enrollment was 29.6 years (SD = 5.1 years). Most (n = 7249; 92.2%) participants were married/cohabitating and approximately half had a college degree (n = 3940; 50.1%). Median EPDS score was 9.0 (IQR 8.0–11.0), and 18.0% (n = 1416) reported MDD history, 11.7% (n = 921) anxiety history, and nearly 20% childhood abuse history (n = 1500; 19.1%). Approximately a third were hospitalized during pregnancy (n = 2943; 37.5%), and a fifth experienced birth complications (n = 1629; 20.7%).

**Table 1:** Sample characteristics in the training set and the independent test set reserved for projection and replication.

Characteristic	Training set N = 5239	Independent test set N = 2620	p-value
Maternal age, mean (SD)	29.69 (5.11)	29.65 (5.07)	0.73
Region, n (%)			0.16
South-East	2786 (53.2%)	1355 (51.7%)	
West	1390 (26.5%)	697 (26.6%)	
Central	759 (14.5%)	383 (14.6%)	
North	304 (5.8%)	185 (7.1%)	
Married or cohabitating, n (%)	4809 (91.8%)	2440 (93.1%)	0.041
Maternal education, n (%)			0.22
Less than HS	755 (14.4%)	410 (15.6%)	
HS diploma and/or some college	1863 (35.6%)	891 (34%)	
College or more	2621 (50.0%)	1319 (50.3%)	
Birth year			0.19
1999–2000	118 (2.3%)	51 (1.9%)	
2001	252 (4.8%)	100 (3.8%)	
2002	421 (8.0%)	224 (8.5%)	
2003	661 (12.6%)	292 (11.1%)	
2004	669 (12.8%)	346 (13.2%)	
2005	728 (13.9%)	406 (15.5%)	
2006	765 (14.6%)	406 (15.5%)	
2007	783 (14.9%)	386 (14.7%)	
2008	610 (11.6%)	297 (11.3%)	
2009	148 (2.8%)	40 (1.5%)	
EPDS score, mean (SD)	9.91 (2.12)	9.88 (2.07)	0.55
Medication use for depression, n (%)	278 (5.3%)	150 (5.7%)	0.47
Medication use for anxiety, n (%)	154 (2.9%)	85 (3.2%)	0.50
History of eating disorder, n (%)	346 (6.6%)	178 (6.8%)	0.79
History of anxiety, n (%)	620 (11.8%)	301 (11.5%)	0.68
History of sexual abuse, n (%)	793 (15.1%)	410 (15.7%)	0.54
History of child abuse, n (%)	1007 (19.2%)	493 (18.8%)	0.69
Pregnancy was planned, n (%)	3744 (71.5%)	1908 (72.8%)	0.22
Maternal prenatal hospitalization, n (%)	1980 (37.8%)	963 (36.8%)	0.38
Initiation of labor, n (%)			0.34
Spontaneous	4079 (77.9%)	2036 (77.7%)	
Induction	772 (14.7%)	368 (14%)	
Caesarean section	388 (7.4%)	216 (8.2%)	
Birth complications, n (%)	1067 (20.4%)	562 (21.5%)	0.28
Child admitted to NICU, n (%)	702 (13.4%)	363 (13.9%)	0.60
Maternal postpartum length of stay ≥4 days, n (%)	1621 (30.9%)	780 (29.8%)	0.30

*Abbreviations:* SD, standard deviation; HS, high school; EPDS, Edinburgh Postnatal Depression Scale; NICU, neonatal intensive care unit.

### Clustering

**Fig. 2:**
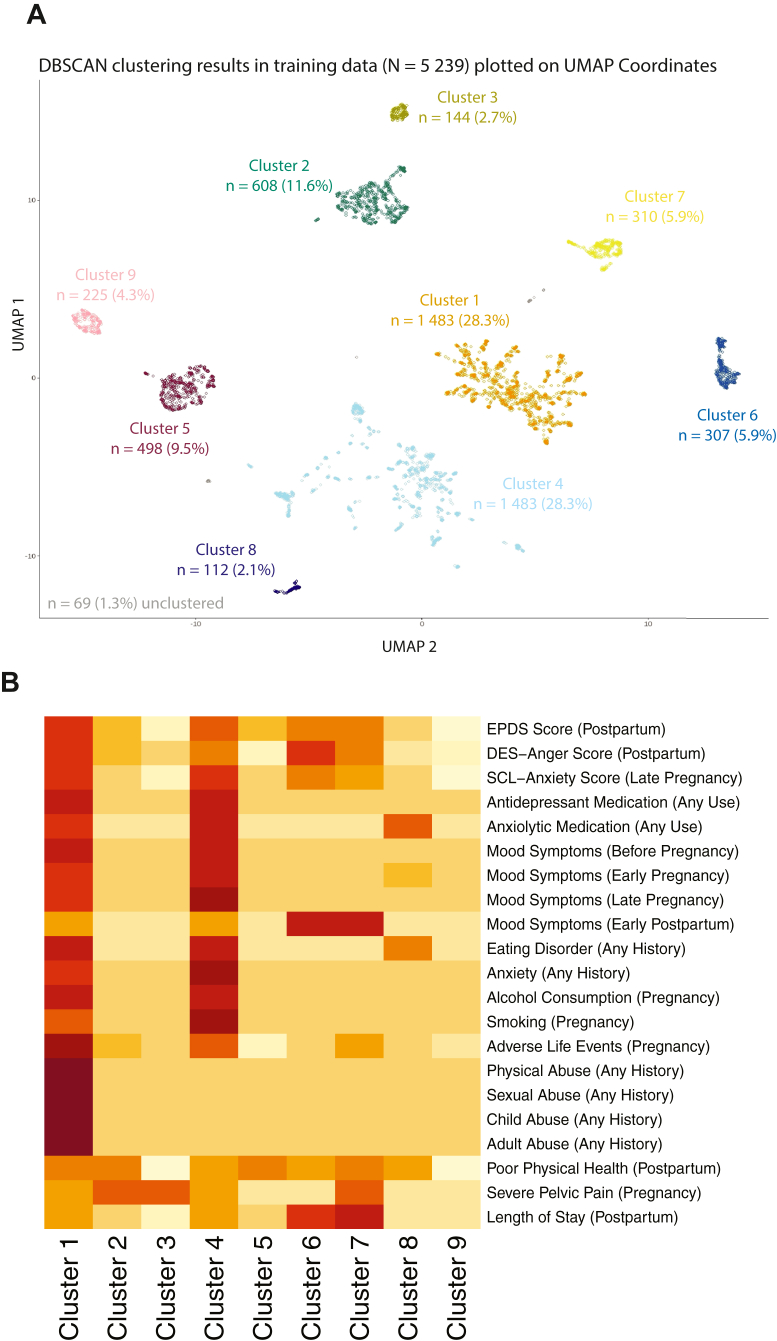
**Clustering output and differentiating features in the training data. (A)** Two-dimensional Uniform Manifold Approximation and Projection (UMAP) of the 5239 PPD cases in the training set. Each point is a mother with PPD in the MoBa cohort; dot colors correspond to the nine clusters identified by the DBSCAN algorithm (Cluster 1—orange, 2—green, 3—olive, 4—sky blue, 5—maroon, 6—blue, 7—yellow, 8—violet, 9—pink). **(B)** Heat map of the scaled input features (rows) by cluster (columns); darker red indicates higher relative values, lighter shades lower values. Together, the panels visualize how women group in symptom space and which features primarily drive membership in each subtype. Comparable plots for the independent test set appear in Figure S1. *Abbreviations:* UMAP, Uniform Manifold Approximation and Projection; DBSCAN, Density-Based Spatial Clustering of Applications with Noise; PPD, postpartum depression; EPDS, Edinburgh Postnatal Depression Scale; DES, Differential Emotions Scale; SCL, Hopkins Symptom Checklist.

Nine clusters were identified in the training sample (N = 5239), ranging in size from 2.1% (n = 110) to 28.3% (n = 1483) of the dataset with 1.3% of individuals (n = 69) remaining unclustered (Fig. 2A). Fig. 2B displays a heatmap of the magnitude of the input variables by cluster. Clusters were mainly distinguished by symptom severity, psychiatric history, history of trauma and abuse, symptom onset and pelvic pain.

In the test set, seven of the nine training clusters were similar in size and character. The two smallest clusters (3 and 8) were not reproduced, but unclustered observations were in the same spatial area (Figure S1A and B). The clusters in the training and testing set had moderate to high concordance (ARI = 0.76 [95% CI 0.74, 0.77], FMI = 0.79 [95% CI 0.77, 0.81]; 1 = perfect concordance and 0 = random for ARI/complete discordance for FMI).

### Cluster characterization

**Fig. 3:**
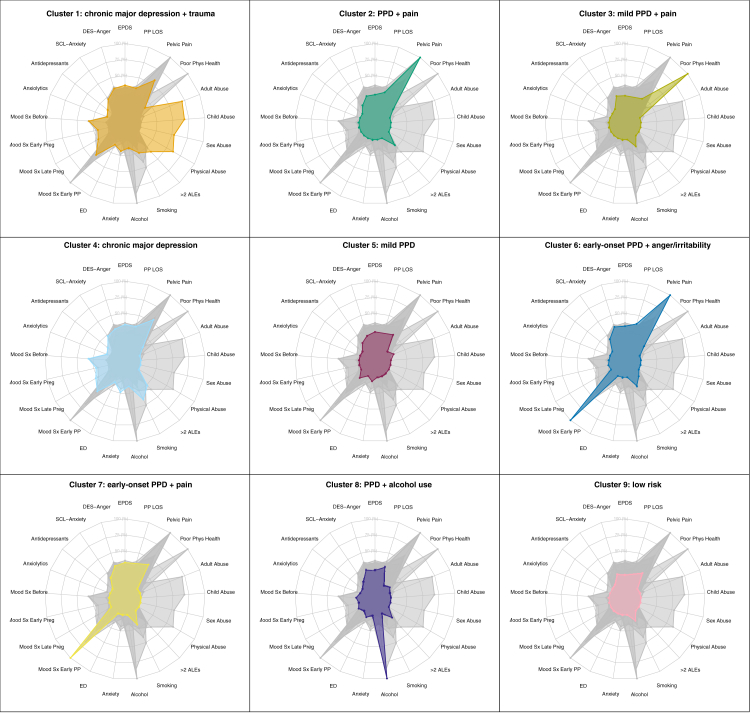
**Radar plots summarizing clinical, psychosocial, and somatic profiles of the nine postpartum depression (PPD) clusters.** Each radar plot displays the profile of each cluster by the feature variables used for clustering. Each spoke on the plot represents the proportion of people in the cluster that have that particular feature, when defined as a binary variable. Clusters are color coded with the same colors as in Fig. 2. Profiles for all clusters are shown in gray in the background for comparison. Higher values denote greater symptom burden or risk-factor prevalence. The visual format highlights how subtypes differ: e.g., Cluster 1 shows pronounced trauma exposure and depressive severity, whereas Cluster 7 is notable for somatic pain and mood symptoms early postpartum. *Abbreviation:* PPD, postpartum depression.

Radar plots in Fig. 3 illustrate the nine training data clusters (test data in Figure S2). Each plot ray represents the percentage of each (binary) input variable within a cluster. Values for each input variable by cluster are presented in Table S7. Cluster 1 and 4 are characterized with more severe PPD symptoms and indicators of psychiatric risk such as medication use, prior psychiatric history, comorbid anxiety, depressive symptoms and substance use during pregnancy, and poorer physical health. They primarily differ in that Cluster 1 exhibits history of abuse, while cluster 4 does not, but is characterized with more depressive symptoms during pregnancy. These clusters will be referred to as Cluster 1 (*depression + trauma*) and Cluster 4 (*depression*).

Individuals in clusters 6 and 7 generally exhibit moderate depressive and anxious symptoms and are distinguished from the other clusters by their experience of PPD symptoms in the early postpartum period, with symptom onset in the first days after childbirth. Individuals in Cluster 6 experienced more anger/irritability (*early-onset + anger*), while those in Cluster 7 experienced severe pain during pregnancy (*early-onset + pain*).

Clusters 2, 3, 5, 8, and 9 consist of individuals with milder symptoms overall. Among these, Cluster 2 was characterized by moderate symptoms while Cluster 9 had fewest symptoms across all groups. Individuals in Clusters 2 and 5 additionally reported poorer physical health, and in clusters 2 and 3 they also reported severe pelvic pain during pregnancy. Cluster 8 was primarily differentiated by alcohol consumption during pregnancy, and more history of eating disorders. These clusters will be referred to as Cluster 2 (*PPD + pain*), Cluster 3 (*mild PPD + pain*), Cluster 5 (*mild PPD*), Cluster 8 (*alcohol*), and Cluster 9 (*low risk*).

### Cluster associations with auxiliary variables

Of the 22 auxiliary variables, 16 significantly differed by cluster in the training set. Summary statistics of auxiliary variables by cluster are presented in Table 2 (training), and Table S8 (test).

**Table 2:** Summary statistics in the training set of auxiliary variables, by cluster.

	Full training set	Unclustered	Cluster 1: chronic major depression + trauma	Cluster 2: PPD + pain	Cluster 3: mild PPD + pain	Cluster 4: chronic major depression	Cluster 5: mild PPD	Cluster 6: early-onset PPD + anger/irritability	Cluster 7: early-onset PPD + pain	Cluster 8: PPD + alcohol use	Cluster 9: low risk	q-value^d^
Total N	5239	69	1483	608	144	1483	498	307	310	112	225	
Maternal age (years), mean (SD)^a^	29.69 (5.11)	29.45 (5.32)	29.50 (5.45)	29.96 (4.43)	29.07 (4.55)	29.44 (5.31)	30.41 (5.01)	29.67 (4.63)	29.55 (4.71)	31.45 (4.59)	30.12 (4.71)	<0.0001
Region, n (%)^c^												0.20
South-East	2786 (53.2%)	45 (65.2%)	795 (53.6%)	300 (49.3%)	76 (52.8%)	796 (53.7%)	257 (51.6%)	161 (52.4%)	160 (51.6%)	73 (65.2%)	123 (54.7%)	
West	1390 (26.5%)	13 (18.8%)	376 (25.4%)	174 (28.6%)	39 (27.1%)	381 (25.7%)	145 (29.1%)	93 (30.3%)	78 (25.2%)	25 (22.3%)	66 (29.3%)	
Central	759 (14.5%)	8 (11.6%)	216 (14.6%)	96 (15.8%)	18 (12.5%)	227 (15.3%)	69 (13.9%)	33 (10.7%)	56 (18.1%)	8 (7.1%)	28 (12.4%)	
North	304 (5.8%)	≤5 (≤7.2%)	96 (6.5%)	38 (6.2%)	11 (7.6%)	79 (5.3%)	27 (5.4%)	20 (6.5%)	16 (5.2%)	6 (5.4%)	8 (3.6%)	
Married/cohabitating, n (%)^b^	4809 (91.8%)	60 (87.0%)	1323 (89.2%)	588 (96.7%)	139 (96.5%)	1322 (89.1%)	474 (95.2%)	286 (93.2%)	302 (97.4%)	105 (93.8%)	210 (93.3%)	<0.0001
Maternal education, n (%)^b^												<0.0001
Less than high school	755 (14.4%)	11 (15.9%)	291 (19.6%)	57 (9.4%)	14 (9.7%)	257 (17.3%)	39 (7.8%)	24 (7.8%)	31 (10.0%)	≤5 (≤4.5%)	26 (11.6%)	
High school diploma or some college	1863 (35.6%)	18 (26.1%)	599 (40.4%)	220 (36.2%)	53 (36.8%)	547 (36.9%)	139 (27.9%)	105 (34.2%)	105 (33.9%)	24 (21.4%)	53 (23.6%)	
College or university degree or more	2621 (50.0%)	40 (58.0%)	593 (40.0%)	331 (54.4%)	77 (53.5%)	679 (45.8%)	320 (64.3%)	178 (58.0%)	174 (56.1%)	83 (74.1%)	146 (64.9%)	
Birth year, n (%)^c^												0.20
1999–2000	118 (2.3%)	0 (0.0%)	20 (1.3%)	20 (3.3%)	≤5 (≤3.5%)	40 (2.7%)	11 (2.2%)	7 (2.3%)	9 (2.9%)	0 (0.0%)	7 (3.1%)	
2001	252 (4.8%)	≤5 (≤7.2%)	68 (4.6%)	29 (4.8%)	≤5 (≤3.5%)	81 (5.5%)	22 (4.4%)	12 (3.9%)	19 (6.1%)	≤5 (≤4.5%)	12 (5.3%)	
2002	421 (8.0%)	6 (8.7%)	133 (9.0%)	45 (7.4%)	10 (6.9%)	121 (8.2%)	39 (7.8%)	24 (7.8%)	25 (8.1%)	8 (7.1%)	10 (4.4%)	
2003	661 (12.6%)	8 (11.6%)	187 (12.6%)	68 (11.2%)	24 (16.7%)	213 (14.4%)	57 (11.4%)	32 (10.4%)	29 (9.4%)	22 (19.6%)	21 (9.3%)	
2004	669 (12.8%)	11 (15.9%)	182 (12.3%)	86 (14.1%)	17 (11.8%)	182 (12.3%)	57 (11.4%)	44 (14.3%)	48 (15.5%)	15 (13.4%)	27 (12.0%)	
2005	728 (13.9%)	7 (10.1%)	204 (13.8%)	77 (12.7%)	15 (10.4%)	228 (15.4%)	71 (14.3%)	44 (14.3%)	42 (13.5%)	15 (13.4%)	25 (11.1%)	
2006	849 (16.2%)	10 (14.5%)	243 (16.4%)	108 (17.8%)	29 (20.1%)	230 (15.5%)	76 (15.3%)	56 (18.2%)	43 (13.9%)	14 (12.5%)	40 (17.8%)	
2007	783 (14.9%)	14 (20.3%)	217 (14.6%)	86 (14.1%)	23 (16.0%)	208 (14.0%)	78 (15.7%)	48 (15.6%)	51 (16.5%)	13 (11.6%)	45 (20.0%)	
2008	610 (11.6%)	10 (14.5%)	183 (12.3%)	73 (12.0%)	13 (9.0%)	148 (10.0%)	71 (14.3%)	34 (11.1%)	31 (10.0%)	16 (14.3%)	31 (13.8%)	
2009	148 (2.8%)	0 (0.0%)	46 (3.1%)	16 (2.6%)	6 (4.2%)	32 (2.2%)	16 (3.2%)	6 (2.0%)	13 (4.2%)	6 (5.4%)	7 (3.1%)	
Relationship satisfaction, mean (SD)^a^	36.10 (7.97)	33.55 (10.68)	34.77 (10.30)	37.02 (8.95)	38.37 (9.50)	35.88 (9.70)	37.21 (9.42)	36.96 (9.60)	36.52 (9.17)	37.39 (9.48)	38.34 (9.23)	<0.0001
Someone for support or advice, n (%)^b^	4701 (89.7%)	67 (97.1%)	1342 (90.5%)	531 (87.3%)	132 (91.7%)	1333 (89.9%)	452 (90.8%)	268 (87.3%)	271 (87.4%)	105 (93.8%)	200 (88.9%)	0.080
Body mass index (kg/m^2^), mean (SD)^a^	24.10 (4.14)	23.27 (4.06)	24.35 (4.35)	24.89 (4.16)	23.64 (3.70)	24.05 (4.11)	23.57 (3.62)	23.46 (4.04)	24.00 (4.71)	23.03 (3.41)	22.69 (2.96)	<0.0001
Anemia, n (%)^c^	313 (6.0%)	6 (8.7%)	116 (7.8%)	39 (6.4%)	9 (6.2%)	76 (5.1%)	28 (5.6%)	12 (3.9%)	14 (4.5%)	≤5 (≤4.5%)	8 (3.6%)	0.043
Ovarian cyst, n (%)^b^	459 (8.8%)	≤5 (≤7.2%)	177 (11.9%)	41 (6.7%)	6 (4.2%)	132 (8.9%)	31 (6.2%)	19 (6.2%)	31 (10.0%)	10 (8.9%)	10 (4.4%)	<0.0001
Migraine, n (%)^b^	773 (14.8%)	7 (10.1%)	270 (18.2%)	80 (13.2%)	14 (9.7%)	227 (15.3%)	43 (8.6%)	39 (12.7%)	58 (18.7%)	14 (12.5%)	21 (9.3%)	<0.0001
Pregnancy was unplanned, n (%)^b^	1495 (28.5%)	20 (29.0%)	521 (35.1%)	125 (20.6%)	29 (20.1%)	469 (31.6%)	120 (24.1%)	59 (19.2%)	67 (21.6%)	30 (26.8%)	55 (24.4%)	<0.0001
Nausea and vomiting of pregnancy, n (%)^b^	2218 (42.3%)	23 (33.3%)	693 (46.7%)	253 (41.6%)	57 (39.6%)	615 (41.5%)	193 (38.8%)	119 (38.8%)	144 (46.5%)	31 (27.7%)	90 (40.0%)	<0.0001
Prenatal hospitalization, n (%)^b^	1980 (37.8%)	25 (36.2%)	626 (42.2%)	188 (30.9%)	36 (25.0%)	611 (41.2%)	152 (30.5%)	110 (35.8%)	128 (41.3%)	32 (28.6%)	72 (32.0%)	<0.0001
Initiation of labor, n (%)^b^												0.013
Spontaneous	4079 (77.9%)	53 (76.8%)	1120 (75.5%)	490 (80.6%)	127 (88.2%)	1142 (77.0%)	394 (79.1%)	249 (81.1%)	229 (73.9%)	87 (77.7%)	188 (83.6%)	
Induction	772 (14.7%)	13 (18.8%)	234 (15.8%)	81 (13.3%)	11 (7.6%)	230 (15.5%)	78 (15.7%)	38 (12.4%)	50 (16.1%)	17 (15.2%)	20 (8.9%)	
Caesarean section	388 (7.4%)	≤5 (≤7.2%)	129 (8.7%)	37 (6.1%)	6 (4.2%)	111 (7.5%)	26 (5.2%)	20 (6.5%)	31 (10.0%)	8 (7.1%)	17 (7.6%)	
Birth felt very unsafe, n (%)^c^	229 (4.4%)	9 (13.0%)	89 (6.0%)	15 (2.5%)	≤5 (≤3.5%)	59 (4.0%)	15 (3.0%)	11 (3.6%)	19 (6.1%)	≤5 (≤4.5%)	≤5 (≤2.2%)	<0.0001
Birth complications, n (%)^b^	1067 (20.4%)	19 (27.5%)	362 (24.4%)	101 (16.6%)	19 (13.2%)	299 (20.2%)	65 (13.1%)	66 (21.5%)	85 (27.4%)	19 (17.0%)	32 (14.2%)	<0.0001
Birthweight (g), mean (SD)^a^	3611 (525)	3616 (607)	3597 (529)	3693 (515)	3636 (490)	3598 (524)	3630 (503)	3581 (535)	3576 (582)	3625 (481.8)	3597 (499)	0.015
Apgar (1 min), mean (SD)^a^	8.7 (1.2)	8.7 (1.1)	8.7 (1.2)	8.7 (1.1)	8.7 (1.0)	8.7 (1.2)	8.6 (1.2)	8.62 (1.37)	8.6 (1.2)	8.7 (1.1)	8.80 (0.89)	0.91
Admitted to NICU, n (%)^b^	702 (13.4%)	17 (24.6%)	217 (14.6%)	69 (11.3%)	14 (9.7%)	190 (12.8%)	60 (12.0%)	47 (15.3%)	45 (14.5%)	14 (12.5%)	29 (12.9%)	0.080
Age at menarche (years), mean (SD)^a^	13.0 (1.5)	13.3 (1.5)	12.8 (1.4)	12.9 (1.4)	13.0 (1.4)	12.9 (1.5)	13.0 (1.4)	13.2 (1.9)	13.1 (1.6)	13.3 (1.6)	13.1 (1.3)	<0.0001
Feels very depressed or irritable before period, n (%)^b^	2098 (40.0%)	29 (42.0%)	702 (47.3%)	221 (36.3%)	42 (29.2%)	637 (43.0%)	139 (27.9%)	114 (37.1%)	118 (38.1%)	34 (30.4%)	62 (27.6%)	<0.0001

*Abbreviations:* SD, standard deviation; EPDS, Edinburgh Postnatal Depression Scale; FDR, False Discovery Rate.

a Kruskal–Wallis rank sum test used to assess differences between clusters.

b Pearson's Chi-squared test used to assess difference between clusters.

c Fisher's Exact Test for count data with simulated p-value (based on 2000 replicates) used to assess difference between clusters.

d q-values reflect False Discovery Rate (FDR) adjusted p-values, calculated using the Benjamini–Hochberg procedure. A q-value of <0.05 indicates statistical significance after accounting for multiple testing.

Clusters 1 (*depression + trauma*) and 4 (*depression*) were associated with putative risk factors and poorer outcomes, including unmarried/partnered status, low education, low relationship satisfaction, more adverse life events during pregnancy, unplanned pregnancy, birth complications, and severe premenstrual mood symptoms (PMS). In general, risk factors and outcomes were worse for Cluster 1. Conversely, clusters 5 (*mild PPD*) and 9 (*low risk*) had the lowest proportion of risk factors and overall better outcomes (Table 2).

Clusters 6 (*early-onset + anger*) and 7 (*early-onset + pain*), although similar in symptom severity and input features, differed in associations with auxiliary variables, particularly those related to physical health. Cluster 7 (N = 310) held the highest or second-highest proportion among all the clusters of health-related variables, including ovarian cyst (n = 31; 10.0%), migraine (n = 58; 18.7%), nausea in pregnancy (n = 144; 46.5%), prenatal hospitalization (n = 128; 41.3%), birth initiation by induction (n = 50; 16.1%) or c-section (n = 31; 10.0%), and birth complications (85; 27.4%). Although maternal pregnancy and birth complications were highest in Cluster 7, infant-related variables were similar across all clusters. Cluster status also corresponded with feelings of safety during labor and delivery. Cluster 7 with more perinatal risks, and Cluster 1 (*depression + trauma*), reported the greatest proportion of people feeling very unsafe during the birth (6.1% [19/310] and 6.0% [89/1483], respectively) (Table 2).

Of the reproductive history variables, mean age at menarche differed slightly between clusters, but median age was 13 years for all clusters. Frequency of severe PMS differed across clusters, with clusters 1 (*depression + trauma*) and 4 (*depression*) being most affected (47.3% [702/1483] and 43.0% [637/1483], respectively), and clusters 6 (*early-onset + anger*) and 7 (*early-onset + pain*) also more affected than the remaining groups (37.1% [114/307] and 38.1% [118/310], respectively) (Table 2).

### PGS analysis

**Fig. 4:**
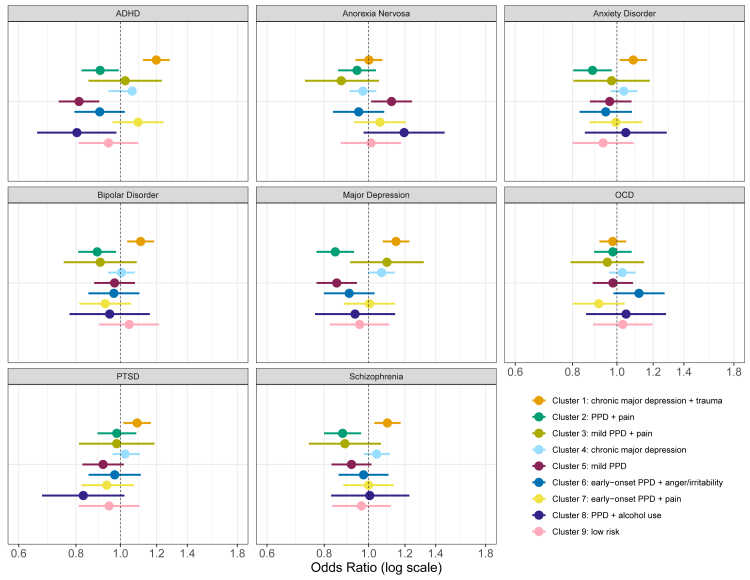
**Associations of polygenic scores (PGS) for neuropsychiatric conditions with cluster membership.** Forest plots of odds ratios (ORs) and 95% confidence intervals for the association between maximized PGS for each neuropsychiatric condition (rows) and the odds of belonging to a given cluster versus all other clusters (columns). Point estimates are shown as filled circles; horizontal lines denote 95% CIs; the vertical dashed line marks OR = 1. The same color scheme used in Figs. 2 and 3 is used to designate each cluster. ORs > 1 indicate higher genetic liability for that trait in the focal cluster; ORs < 1 indicate lower liability. *Abbreviations:* PGS, polygenic scores; OR, odds ratio; ADHD, attention deficit hyperactivity disorder; OCD, obsessive compulsive disorder; PTSD, post-traumatic stress disorder; PPD, postpartum depression.

In the training set, there were differences in mean PGS of ADHD, MDD, and schizophrenia across clusters (Table S10). PGS for all conditions except anorexia nervosa and OCD were associated with higher odds of being in Cluster 1 (*depression + trauma*) with odds ratios (OR) and 95% Confidence intervals (CI) for the PGS of ADHD (1.19, 1.12–1.28), anxiety disorder (1.09, 1.01–1.16), bipolar disorder (1.11, 1.03–1.19), MDD (1.15, 1.07–1.23), PTSD (1.09, 1.02–1.17), and schizophrenia (1.10, 1.03–1.17) (see Fig. 4; and Figure S3 for test data). MDD PGS also increased odds of being in Cluster 4 (*depression*; 1.07, 1.00–1.14).

Conversely, odds of being in Cluster 2 (*PPD + pain*) compared with other clusters was associated with lower PGS for all the same conditions except PTSD (ADHD: 0.90, 0.82–0.99; anxiety disorder: 0.88, 0.80–0.97; bipolar disorder: 0.89, 0.80–0.98; MDD: 0.85, 0.77–0.93; schizophrenia: 0.88, 0.80–0.96). Cluster 5 (*mild PPD*) was also inversely associated with ADHD (0.81, 0.82–0.99) and MDD PGS (0.85, 0.77–0.96), though positively associated with anorexia nervosa PGS (1.12, 1.01–1.24), the only cluster to have a significant association with anorexia PGS.

The association of ADHD PGS with lower odds of being in cluster 8 (alcohol: 0.80, 0.66–0.98) was the only significant PGS association with any of the three smallest clusters (8, 3, and 9). Given the exploratory nature and numerous associations examined, we primarily present findings for associations with p < 0.05, however, full PGS model output is provided in Table S11 and we emphasize interpretation based on odds ratios and corresponding compatibility intervals (95% confidence intervals)—alongside statistical significance—to provide balanced clarity regarding clinical relevance.

### Replication of auxiliary variable and PGS results

Of the 16 auxiliary variables that differed by cluster in the training sample, ten also differed in our test sample: marital status, education, relationship satisfaction, body mass index, ovarian cyst, migraine, unplanned pregnancy, nausea in pregnancy, prenatal hospitalization, and severe PMS. The distribution patterns by cluster were similar in both samples, with cluster percentage of binary/categorical auxiliary variables meeting FDR significance in both the training and test sets (Figure S4).

Mean PGS differences for ADHD and MDD replicated in the test set, but not for schizophrenia. Table 3 presents the cluster-PGS associations in the training and test sets. Positive associations between ADHD, bipolar disorder, and PTSD PGS with Cluster 1 (*depression + trauma*) replicated, but associations between anxiety disorder and MDD PGS did not. The positive association between MDD PGS and Cluster 4 (*depression*), replicated. Of the negative associations between PGS and cluster, those for cluster 5 (*mild PPD*) replicated, but those for cluster 2 (*PPD + pain*) did not.

**Table 3:** Direction of associations in the training sample between clusters and polygenic scores for neuropsychiatric conditions, with significant associations highlighted in light gray.

Significant associations that further replicated in the projected test set are highlighted in dark gray.

*Abbreviations:* PPD, postpartum depression; ADHD, attention deficit hyperactivity disorder; AN, anorexia nervosa; ANX, anxiety; BIP, bipolar disorder; MDD, major depressive disorder; OCD, obsessive compulsive disorder; PTSD, post-traumatic stress disorder; SCZ, schizophrenia.

### Summary of cluster differences

To summarize the key cluster differences and associations: Cluster 1 (n = 1483; 28.3% of sample) was characterized by chronic depression with trauma history and had the strongest genetic associations with psychiatric conditions. Cluster 4 (n = 1483; 28.3%) showed similar depression severity but without significant trauma history. Clusters 6 and 7 (n = 307; 5.9% and n = 310; 5.9% respectively) both exhibited early-onset symptoms but were differentiated by anger/irritability (Cluster 6) versus pain and physical health issues (Cluster 7). The remaining clusters represented milder symptom profiles, with Cluster 9 (n = 225; 4.3%) showing the fewest symptoms across all domains.

In analysis of cluster relationships with auxiliary variables, clusters 1 and 4 (severe chronic depression with and without trauma) were distinct in terms of low relationship satisfaction, high physical and mental health burden, lower education, and reduced social and economic capital. Cluster 7 (early-onset PPD + pain) is notable for both physiological sensitivity (e.g., migraine, nausea, ovarian cysts) and higher obstetric risk, suggesting hormonal or somatic vulnerability. Cluster 9 (low risk) had the most protective profile across nearly all domains.

## Discussion

Our study applied data-driven unsupervised clustering approaches on extensive longitudinal phenotypic and genotypic data to identify PPD subtypes. We identified nine clusters with high reproducibility (seven were highly concordant in the test set). The clusters confirmed previously identified PPD subgroups and identified several novel subgroup characteristics. The PPD clusters were differently associated with obstetrical factors, reproductive factors, and PGS for psychiatric and neurodevelopmental conditions, providing evidence that they correspond with clinically relevant subtypes of PPD.

As supported by prior studies that applied LCA to postpartum women,^11^ we found that symptom severity and timing of onset distinguished subgroups. History of abuse and trauma is a well-known risk factor for PPD,^16^ but has not previously been explored as a differentiator of subtypes. We found exacerbated negative outcomes in subgroups of women with PPD with a history of trauma and abuse, also the sole factor differentiating the two clusters with most severe and chronic symptoms (clusters 1 and 4). Another novel finding suggests severe pain during pregnancy as a distinguishing feature, particularly among groups with early-onset or less severe PPD symptoms (clusters 2, 3, and 7). These findings highlight the importance of trauma-informed care in the treatment of perinatal mood disorders, and importance of pain treatment during pregnancy for subgroups.

Consistent with studies of risk of PPD generally, not surprisingly, the most severe clusters were associated with socioeconomic risk factors. For example, clusters 1 and 4 had the largest percentage of those who did not complete high school (19.6% [291/1483] and 17.3% [257/1483], respectively). This association between lower education and the most severe PPD clusters highlights the importance of social determinants of health in perinatal mental health outcomes. Future research should further explore how socioeconomic disparities contribute to different PPD presentations and whether interventions addressing these social factors might be particularly beneficial for women in these higher-risk clusters.

Our findings indicate the important contribution of obstetrical and reproductive factors to PPD, which have implications for treatment, particularly in conjunction with findings about pain. Although PPD is considered a *psychiatric* health condition, we found that factors affecting *physical* health differentiated PPD subgroups. Pregnancy alters nearly every system in the human body to protect a growing fetus,^47^ many of which cause dysregulation or pain for the pregnant mother.^47^ Despite well-studied associations between pelvic pain and mood symptoms,^48^ which can be severe and functionally debilitating, these problems are often dismissed as mild discomforts of pregnancy. Severe pelvic pain that kept women awake at night was experienced by over half of our sample and one of the most important factors defining PPD subgroups. Both maternal and infant birth complications are known risk factors for PPD, but interestingly, only maternal complications differentiated subgroups. This finding is somewhat surprising given the established relationship between neonatal intensive care unit (NICU) admission and increased PPD risk. One possible explanation is that our clustering approach identified subgroups based primarily on intrinsic maternal factors rather than infant-related stressors, suggesting that infant complications might exacerbate PPD symptoms across all subtypes rather than defining a specific subtype. The prenatal period can be an opportunity for setting up structures of assessing and supporting the mother before the baby is born, as pregnancy is a time of frequent and regular interaction with the health care system.

Cluster 7 (*early-onset PPD + pain*) was strongly associated with several hormonal and reproductive conditions, such as nausea during pregnancy, ovarian cysts, migraine, and severe PMS. The rapid hormonal changes during labor and delivery may particularly affect women who are sensitive to such fluctuations.^15^ While overall hormone levels are not associated with PPD in observational studies, our findings align with results from small experimental studies suggesting that a subset of women are sensitive to hormonal changes that influence mood.^15^^,^^49^ Cluster 7 might suggest potential population-level identifiers of hormone sensitivity that could be used for further observational research on the efficacy of hormone-related treatments.

The genetic profiles of the clusters, as measured by polygenic scores (PGS, which quantify genetic liability by summing weighted genetic variants associated with a condition), support our identified subtypes and suggest new directions for PPD research. The most consistent associations were between clusters with severe PPD symptoms and MDD PGS, an expected association due to high genetic correlation between MDD and PPD.^22^ Also as expected, PGS for most psychiatric and neurodevelopmental conditions were positively correlated with clusters with the most severe symptoms, and negatively correlated with clusters with the least severe symptoms. Interestingly, our findings support an underlying genetic signature for trauma, which has been demonstrated broadly, but only recently examined in women with PPD.^50^ The two depression clusters differed in trauma history. The cluster with more trauma history was significantly associated with PTSD PGS but not with MD PGS; conversely, the pattern was reversed in the other group.

A less investigated but growing area of research is ADHD in women during pregnancy and postpartum.^51^ The ADHD PGS had the greatest number of replicated PGS relationships with clusters. Although the importance of these findings is unclear, they suggest that further investigation of symptomology more consistent with ADHD than mood disorders might be informative for disentangling heterogeneity.

It is important to note that many of the odds ratios for PGS associations with clusters were relatively modest (OR < 1.2), reflecting small effect sizes. This finding aligns with the known polygenic nature of psychiatric conditions, where multiple genetic variants each contribute incrementally. Additionally, our study design intentionally included only postpartum depression (PPD) cases to specifically explore nuances in heterogeneity among affected individuals. Consequently, effect sizes for comparisons between clusters were modest, as we used the affected individuals not in the subgroup of investigation as the reference rather than asymptomatic controls. Had we instead used asymptomatic participants as the reference group, we likely would have observed larger overall effect sizes but reduced differentiation among clinically relevant PPD subtypes. Therefore, the modest odds ratios observed highlight that genetic factors explain only a portion of the variance distinguishing these clusters, underscoring the substantial role of environmental and other non-genetic contributors in PPD etiology.

Overall, these findings reveal important clinical implications regarding how different psychiatric symptom profiles relate to PPD subtypes. Cluster 1 (depression + trauma) exhibited the strongest genetic associations with multiple psychiatric conditions, suggesting that this subtype may represent a more biologically-driven form of PPD with higher psychiatric vulnerability overall. The clear distinction between Clusters 1 and 4, which differed primarily in trauma history despite similar depression severity, supports the need for trauma-specific assessment and intervention in PPD care. The association between ADHD polygenic scores and Cluster 1 is particularly notable, as attention and executive function difficulties could interfere with treatment adherence and self-care routines during the postpartum period, potentially requiring additional support strategies.

It is necessary to consider limitations of the study when interpreting these results. All the PPD outcome data in this study are self-reported. Data from the Norwegian Patient Registry began is available from 2008, the final year of MoBa recruitment, therefore we do not have medical records to validate self-reported responses. Self-reported outcomes, however, are arguably appropriate for mental health conditions such as PPD, since suffering from depression and anxiety symptoms and functional impairment are defined mainly based on subjective descriptive criteria. There are also specific limitations to specific variables within the survey data. Our measure of pain was based on a single binary question about being awakened at night by pelvic pain. While this captures severe pain with functional impact, it does not assess pain intensity, duration, or quality. Our data on caesarean deliveries did not distinguish between planned and emergency procedures, which have been shown to have different associations with PPD risk.^52^^,^^53^ This limitation prevents us from examining the potentially important relationship between birth mode urgency and PPD subtypes. The MoBa surveys also did not inquire about suicidal ideation or self-harm, previously shown to be important factors in differentiating PPD subgroups.^19^^,^^20^

Unmeasured confounding is a potential concern, particularly regarding socioeconomic factors beyond education that might influence both cluster membership and outcomes. Model misspecification bias is also possible, as our clustering approach necessarily involves dimensional reduction that may not fully capture all relevant aspects of PPD heterogeneity.

Although our dataset was large enough to enable data splitting for validation, some cluster replication was likely due to the similarity of the training set and the test set. Norway is also ancestrally homogeneous, and the current quality-controlled genetic data only includes individuals of European ancestry, which limits generalizability to diverse ancestries. Lastly, sample sizes for GWAS of some conditions (e.g., anorexia, OCD) are still underpowered and limit our ability to detect genetic effects.

Moving forward, these findings should be replicated in external and more diverse samples to investigate whether the PPD subtypes and associations partition in broader social environments and hold true in non-European samples. More research is also needed on the interactions between trauma, pain, and perinatal risks with PPD. Future studies should also include detailed assessment of pain management approaches and medication use, as these might moderate the relationship between pain and PPD symptoms. Our study is a step on the path toward precision psychiatry, and genetic research in this area should focus on gene by environment interactions and more nuanced genetic analyses such as inclusion of a PGS from the first large PPD GWAS,^22^ and non-psychiatric PGS (e.g., migraine, pain, etc.). Future work should also cross-validate observational and experimental results among overlapping samples to identify non-invasive indicators of hormonal sensitivity. With the availability of the first US Food and Drug Administration (FDA) approved pharmacological treatments indicated specifically for PPD,^54^^,^^55^ a population-level indicator of hormone sensitivity could establish whether these PPD-indicated treatments provide a better response than traditional treatment for depressive symptoms in certain subgroups. In addition to biological inquiries, researchers should evaluate specific interventions *during pregnancy* focused on addressing trauma, managing pain, and enhancing emotional support.

Delivering the right treatment to the right patient at the right time—the essence of precision medicine—can vastly improve patient outcomes.^56^ While precision medicine has advanced in many fields, psychiatry has lagged behind.^57^ The complexity of the brain, its interrelationship with other systems, and diverse treatment options make precision psychiatry challenging, yet present tremendous opportunities. Our study advances precision psychiatry by identifying distinct PPD subtypes through self-reported data, validated by clinical outcomes and genetic correlates. These data-driven subgroups—differentiated by severity, onset, trauma history, and pain profiles—reveal new insights into the complex interplay between psychological, social, obstetric, hormonal, and genetic factors in PPD. Our findings demonstrate the need for integrated care approaches beyond traditional psychiatric treatments, emphasizing the importance of trauma-informed obstetric care and pain management. The identification of potential non-invasive hormonal and genetic indicators also opens new avenues for targeted therapeutic development and patient stratification in clinical trials. The frequency and consistency of medical care throughout pregnancy provides an ideal setting to apply subtype-specific insights and develop tailored PPD interventions. As precision medicine gains traction in psychiatry, this study represents a crucial step toward optimizing personalized treatment protocols to improve patient outcomes and reduce the devastating impact of PPD on mothers, children, and families.

## Contributors

AEB conceptualized the study, designed the analysis plan, performed statistical analyses, and led manuscript writing. PJ, LJH, MT, and HA contributed to statistical analysis and provided analytical feedback. YLu, JG, LJH, ECC, and HA assisted with data preparation and quality control. OAA, YLi, TR-K, and HA oversaw data collection, analytical planning, and manuscript development. PFS, SM-B, AH, and HA contributed to study conceptualization, analytical planning, field-specific consultation, and manuscript writing. AEB, LJH, ECC, AH, and HA have access to and verify the underlying study data. All authors reviewed and approved the final manuscript.

## Data sharing statement

Data from the Norwegian Mother, Father and Child Cohort Study and the Medical Birth Registry of Norway used in this study are managed by the national health register holders in Norway (Norwegian Institute of Public Health) and can be made available to researchers, provided approval from the Regional Committees for Medical and Health Research Ethics (REC), compliance with the EU General Data Protection Regulation (GDPR) and approval from the data owners. The consent given by the participants does not open for storage of data on an individual level in repositories or journals. Researchers who want access to data sets for replication should apply through helsedata.no. Access to data sets requires approval from The Regional Committee for Medical and Health Research Ethics in Norway and an agreement with MoBa. R scripts used for analysis are available at https://github.com/Annabetsy/ppd_subtypes.

## Declaration of interests

All authors declare no competing interests.
