# Supplementary material for: Identification and validation of postpartum depression subtypes: a population-based cohort study
Source: eClinicalMedicine. 2025 Oct 9;89:103540. doi: 10.1016/j.eclinm.2025.103540 (PMC12546979; doi:10.1016/j.eclinm.2025.103540)
Supplement: Supplementary Materials [file mmc1.pdf]

SUPPLEMENTARY MATERIALS

CONTENTS

Supplementary Figures
Figure S1. Clustering output and differentiating features in the projected independent test data
Figure S2: Radar plots and characterization of each of the nine clusters in the projected independent test data
Figure S3. Forest plots of odds ratios for the association between polygenic score for each neuropsychiatric trait and cluster membership in the projected independent test data
Figure S4. Percentages of each variable by cluster for auxiliary variables that had replicated significant associations in both the training and projected independent test sets

Supplementary Tables
Table S1. Variables used as features in clustering algorithm
Table S2. Auxiliary variables used for characterization of clusters
Table S3. Parameters used in PRSice2
Table S4. Number of SNPs to calculate PGS at each p-value threshold and proportion of variance explained by PC1 of each trait
Table S5. Filtered SNPs in PGS Generation
Table S6. GWAS Summary Statistics Sources for Psychiatric Disorders
Table S7. Summary statistics of feature variables used in clustering algorithm by cluster
    Table S7a. Summary of feature variables in training set
    Table S7b. Summary of feature variables in replication set
Table S8: Summary statistics of auxiliary variables by cluster, projected independent test set
Table S9. Box-Tidwell tests of linearity for logistic PGS models
Table S10. Mean polygenic score for each neuropsychiatric condition, by cluster
    Table S10a. Mean (SD) PGS for each condition by cluster, training set
    Table S10b. Mean (SD) PGS for each condition by cluster, projected independent test set
Table S11. Associations in logistic model between PGS for each condition and cluster (binary, 1=in the specific cluster, 0=in any other cluster)
    Table S11a. PGS for each condition by cluster (binary), training set
    Table S11b. PGS for each condition by cluster (binary), projected independent test set
Table S12. Multinomial model with cluster as variable with 9 levels; cluster 9 (low risk) as reference category
    Table S12a. Cluster 9 (low risk) as reference category
    Table S12b. Cluster 1 (chronic major depression + trauma) as reference category

Supplementary Methods
Methods
Plots - Diagnostics, Parameter Turning, and Sensitivity Analyses
Research in Context - Full List of Terms Searched in NHGRI-EBI GWAS Catalog

SUPPLEMENTARY FIGURES

Figure S1. Clustering output and differentiating features in the projected independent test data. (**A)** Independent test data were projected onto the same UMAP manifold as the training data and the DBSCAN algorithm was run separately on the projected test data with the same parameters as in the training data. The algorithm identified seven clusters in the projected independent test data that were similar to clusters from the nine identified in the testing data. Colored dots represent the seven clusters identified by the DBSCAN clustering algorithm. (**B)** A heat map shows the relative values of input feature variables for each of the seven clusters in the test data to visualize the primary features driving cluster membership. Darker red indicates higher relative values.

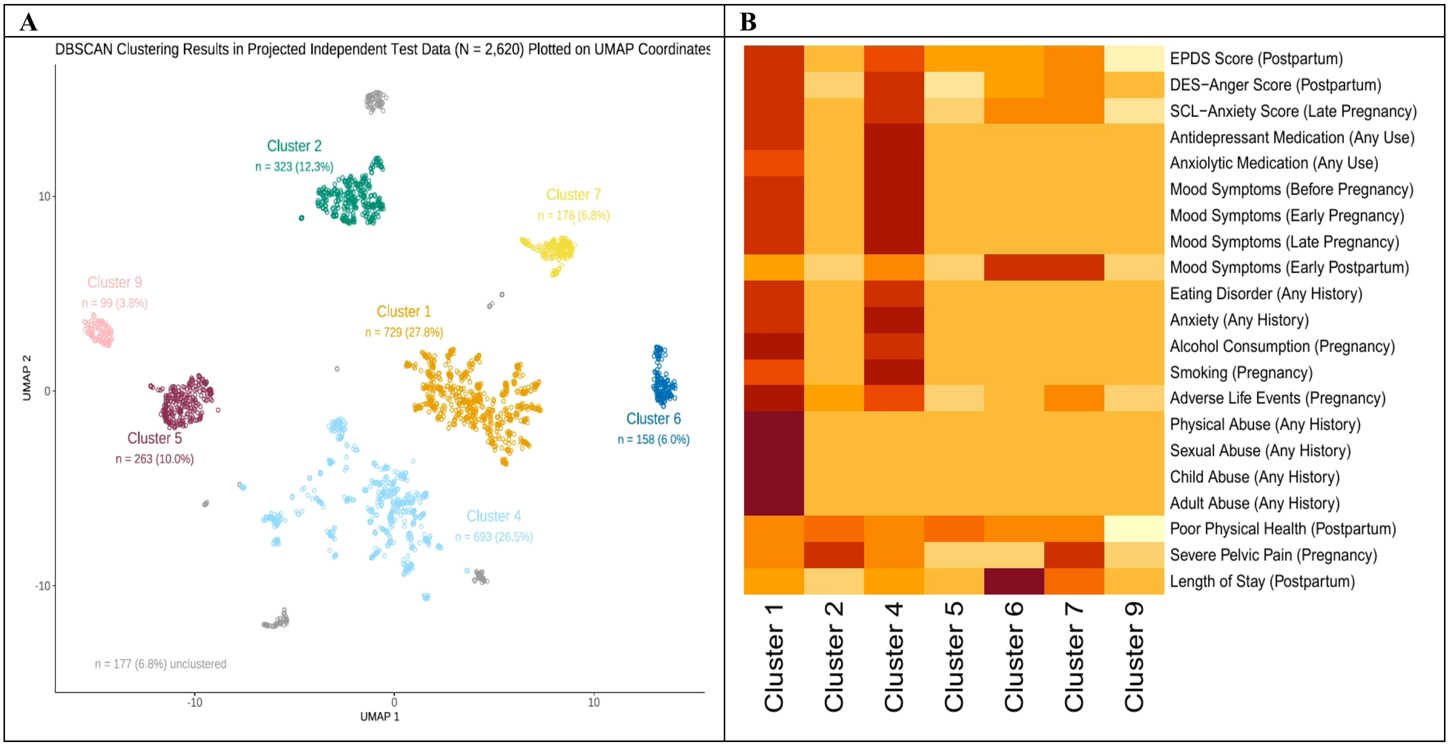

Figure S2: Radar plots and characterization of each of the nine clusters in the projected independent test data

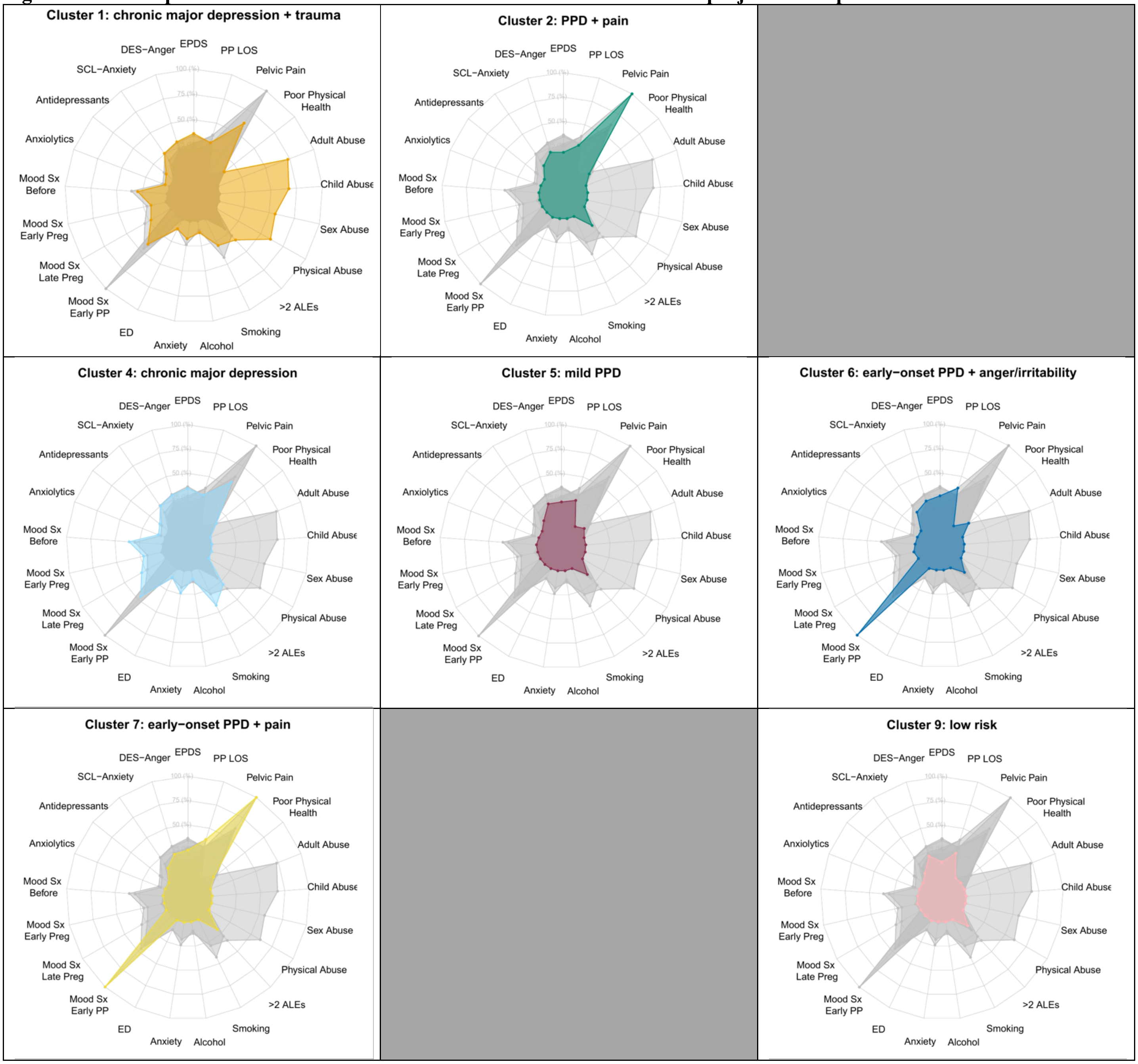

Figure S3. Forest plots of odds ratios for the association between polygenic score for each neuropsychiatric trait and cluster membership in the projected independent test data

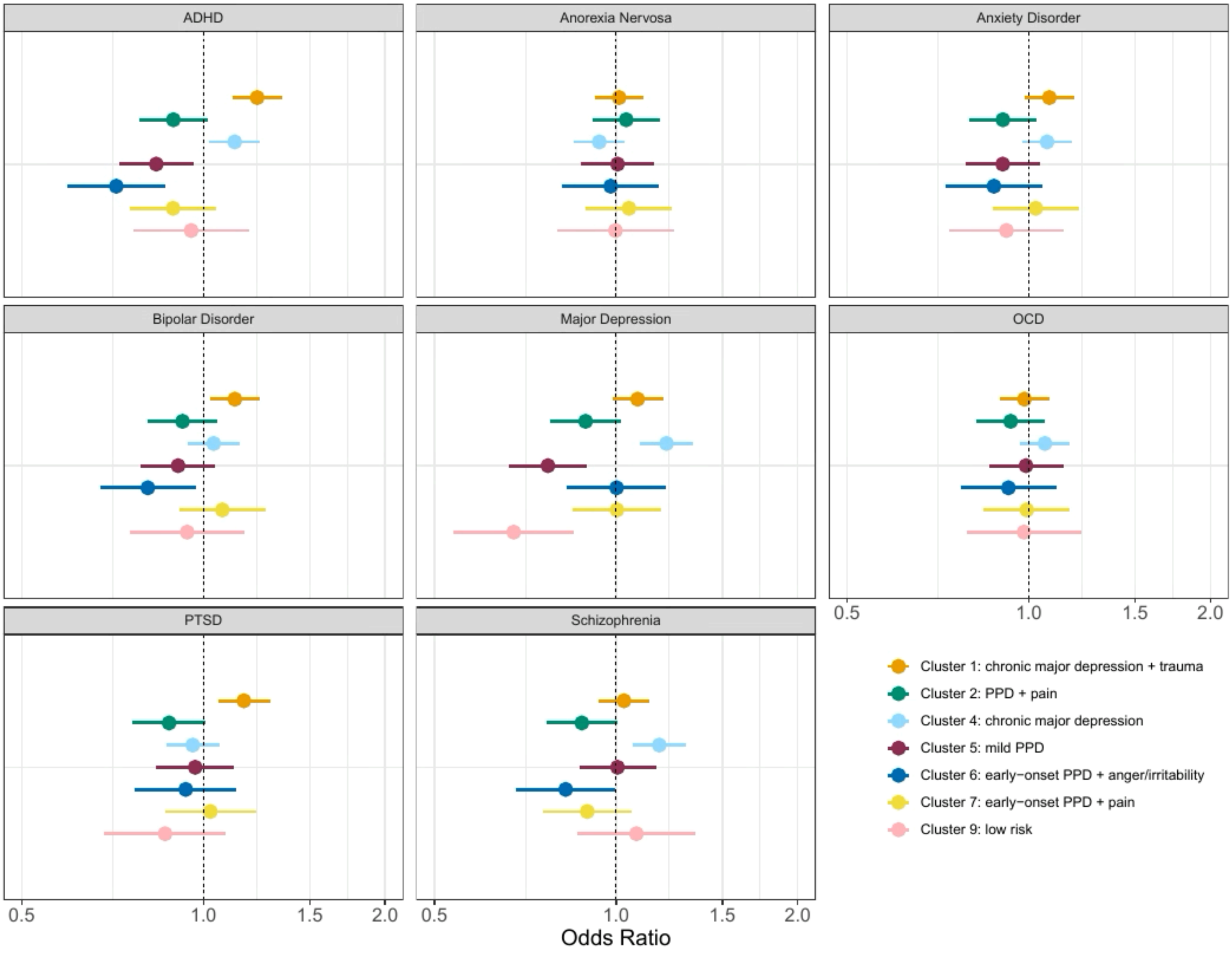

Figure S4. Percentages of each variable by cluster for auxiliary variables that had replicated significant associations in both the training and projected independent test sets

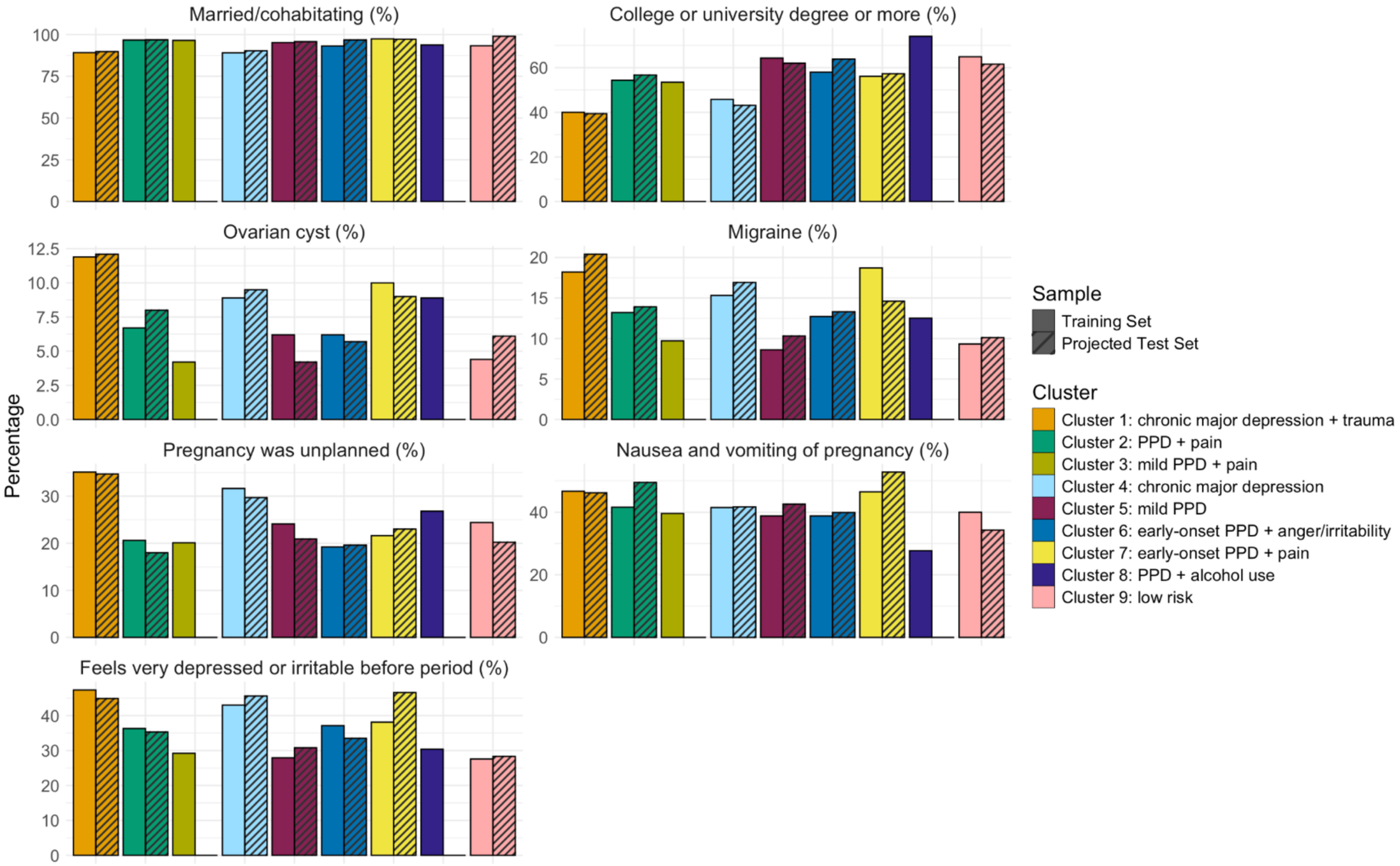

SUPPLEMENTARY TABLES

Supplementary Table S1: Variables used as features in clustering algorithm

Variable Name	Description	Definition	Coding	Type	Rationale
Severity					
N.epds	EPDS sum score, 6m postpartum	Edinburgh Postnatal Depression Scale sum score, Q6m	Score value	Numeric	EPDS score is a measure of symptom severity
N.des.anger	Anger subscale symptom score, 6m postpartum	Differential Emotions Scale - anger subscale score	Score value	Numeric	Depression and anxiety sometimes present as anger and irritability
N.scl.anx.pp.div	Anxiety subscale symptom score, 3rd trimester	Hopkins Symptoms Checklist - anxiety subscale score; scaled to clinical categories	Score value	Numeric	Comorbid anxiety is particularly prevalent during the perinatal period
I.meds.dep	Use of medication for depression	Reported taking medication for depression, any timepoint	Yes/No	Binary	Medication use may indicate more severe depression
I.meds.anx	Use of medication for anxiety	Reported taking medication for anxiety, any timepoint	Yes/No	Binary	Medication use may indicate more severe psychopathology
Psychiatric History					
I.hx.ed	History of eating disorder before pregnancy	Reported history of anorexia nervosa, bulimia, other eating disorder, Q1	Yes/No	Binary	History of psychopathology is a risk factor for PPD
I.hx.anx	History of anxiety before pregnancy	Reported history of anxiety, Q1	Yes/No	Binary	History of psychopathology is a risk factor for PPD
I.sx.bef.preg	History of depression before pregnancy	Reported previous history of depression or medication use before pregnancy	Yes/No	Binary	PPD may differ in new-onset vs people with history of depression
Symptom Timing					
I.sx.early.preg	Depressive symptoms, first trimester	Q1 SCL-5item score >=2.0 or reported depression on Q1	Yes/No	Binary	PPD may differ in new-onset vs people with history of depression
I.sx.late.preg	Depressive symptoms, third trimester	Q3 SCL-8item score >=1.85 or reported depression on Q3	Yes/No	Binary	PPD may differ in new-onset vs people with history of depression
F.sx.early.pp	Depressive symptoms, early postpartum	Reported feeling depressed in the time just after birth, Q6m	Yes/No	Binary	PPD may differ in new-onset vs people with history of depression
Substance Use					
I.alc.preg	Alcohol use during pregnancy	Any reported alcohol use during pregnancy, Q1 or Q3	Yes/No	Binary	Alcohol use during pregnancy may indicate addiction or prior psychopathology
I.smoke.preg	Smoking during pregnancy	Any reported smoking during pregnancy, Q1 or Q3	Yes/No	Binary	Smoking during pregnancy may indicate addiction or prior psychopathology
Trauma History					
N.ale.preg	Adverse life events, early pregnancy and prior year	Adverse Life Events score in last year, Q1	Number of events	Numeric	Adverse life events are associated with PPD
I.ph.abuse	Lifetime history of physical abuse	Any reported lifetime physical abuse, Q3	Yes/No	Binary	Trauma history is a risk factor for PPD
I.sex.abuse	Lifetime history of sexual abuse	Any reported lifetime sexual abuse, Q3	Yes/No	Binary	Trauma history is a risk factor for PPD; sexual trauma may distinguish different trajectory
I.ch.abuse	History of child abuse	Any reported physical or sexual abuse prior to age 18, Q3	Yes/No	Binary	Trauma history is a risk factor for PPD; early trauma may distinguish different trajectory
I.ad.abuse	History of adult abuse	Any reported physical or sexual abuse at age 18 or older, Q3	Yes/No	Binary	Trauma history is a risk factor for PPD
Physical Impairment					
F.phys.health	Self-rated physical health, 6m postpartum	Overall, how would you describe your physical health at the moment?, Q6m	Very good, Good, Poor, Very Poor	Ordered Factor	Physical well-being may suggest differences in functional ability
F.pain	Severe pelvic pain, 3rd trimester	Reported waking at night due to pelvic pain during pregnancy, Q3	Yes/No	Binary	Pregnancy complications are a risk factor for PPD
N.los.m.pp	Postpartum maternal length of stay	Reported maternal length of hospital stay after delivery, Q6m	Number of days	Numeric	Birth complications are associated with PPD; Longer length of stay may indicate more serious complications

Supplementary Table S2: Auxiliary variables used for characterization of clusters

Variable Name	Description	Definition	Coding	Type	Rationale
Demographic Factors					
N.age	Maternal age at delivery	Maternal age at delivery	Age in years	Numeric	Age is a risk factor for PPD; lower and higher ages higher risk
F.region	Region	Region of residence at enrollment	Southeast, Central, Wes	Categorical	PPD may differ by region
F.marstat	Marital or partner status	Indicated married or cohabitating	Yes/No	Binary	Partner status is protective for PPD
F.edu	Maternal education	Mother's highest level of education attained	Less than high school, Hi	Ordered Factor	Low education is a risk factor for PPD
Social Support					
N.rel.satis	Relationship satisfaction sum score, 6m postpartum	Relationship Satisfaction Scale sum score, Q6m	Score value	Numeric	Positive relationship is protective for PPD
I.sup.advice	Have someone for support	Do you have anyone other than husband/partner you can ask for advice?, Q1	Yes/No	Binary	Social support is protective for PPD
Medical Comorbidities					
N.bmi	Pre-pregnancy Body Mass Index	Body Mass Index in height/weight in meters/kg^2, Q1	Continuous BMI in m/kg	Numeric	Overweight/obesity is a risk factor for PPD
I.anemia	Anemia	Reported low hemoglobin or anemia, Q1	Yes/No	Binary	Anemia is associated with mood changes
I.ovcyst	Ovarian cyst	Reported ovarian cyst, Q1	Yes/No	Binary	Reproductive illnesses and pain may share mechanisms with PPD
I.migraine	Migraine	Reported migraine, Q1	Yes/No	Binary	Migraine may share mechanisms with depression
I.diabetes	Pre-pregnancy diabetes	Reported diabetes before pregnancy, Q1	Yes/No	Binary	Poor physical health is a common comorbidity of PPD
I.heartdis	Heart disease	Reported heart disease, Q1	Yes/No	Binary	Poor physical health is a common comorbidity of PPD
I.htn	Pre-pregnancy hypertension	Reported hypertension before pregnancy, Q1	Yes/No	Binary	Poor physical health is a common comorbidity of PPD
I.thyroid	Thyroid problems	Reported hypo/hyperthyroidism, Q1	Yes/No	Binary	Thyroid/endocrine problems are associated with mood changes
I.autoimmune	Autoimmune disease	Reported autoimmune disease (Crohns, colitis, celiac, rheumatoid arthritis, Lupus), Q1	Yes/No	Binary	Autoimmune diseases may share mechanisms with depression
I.endo	Endometriosis	Reported endometriosis, Q1	Yes/No	Binary	Reproductive illnesses and pain may share mechanisms with PPD
I.cancer	Cancer	Reported cancer, Q1	Yes/No	Binary	Cancer is a medical trauma and trauma history is a risk factor for PPD
Pregnancy Complications					
F.pregplan	Pregnancy was unplanned	Response to - Was this pregnancy planned?, Q1	Yes/No	Binary	Unintended pregnancy is a risk factor for PPD
I.nvp	Nausea and vomiting of pregnancy	Reported problems with nausea and/or vomiting during pregnancy, Q1 or Q3	Yes/No	Binary	Pregnancy complications are a risk factor for PPD
F.m.prenat.hosp	Maternal prenatal hospital admission	Mother admitted to hospital during pregnancy, Q3	Yes/No	Binary	Pregnancy complications are a risk factor for PPD
F.infertility	Infertility	Ever received treatment for infertility, Q1	Yes/No	Binary	Infertility is a risk factor for PPD
I.gest.diab	Gestational diabetes	Gestational diabetes indicated in Medical Birth Registry	Yes/No	Binary	Pregnancy complications are a risk factor for PPD
I.pe.hellp.ecl	Preeclampsia/HELLP/Eclampsia	Preeclampsia/HELLP/Eclampsia indicated in Medical Birth Registry	Yes/No	Binary	Pregnancy complications are a risk factor for PPD
Delivery and Birth Factors					
F.deliv.start	Initiation of labor	Initiation of labor indicated in Medical Birth Registry	Sponteneous, Induction,	Ordered Factor	Cesearean birth is associated with PPD
F.bir.feltsafe	Felt safe during labor and childbirth	Response to - I felt safe and in good hands during the birth, Q6m	Fairly true, Partially true	Ordered Factor	Childbirth experience is associated with PPD
F.birth.comp	Birth complications	Reported complications during the birth	Yes/No	Binary	Birth complications are associated with PPD
Infant Complications					
N.birthweight	Birthweight	Birthweight reported in Medical Birth Registry	Weight in grams	Numeric	Neonatal complications are a risk factor for PPD
N.apgar.1	1 min Apgar score	Apgar score at 1 minute after delivery reported in Medical Birth Registry	Score value	Numeric	Neonatal complications are a risk factor for PPD
F.c.admit	Infant NICU admission	Mother reported baby was admitted or transferred to another hospital, Q6m	Yes/No	Binary	Neonatal complications are a risk factor for PPD
I.resp.dist	Infant respiratory distress	Infant repiratory distress indicated in Medical Birth Registry	Yes/No	Binary	Neonatal complications are a risk factor for PPD
I.antibio	Infant given antibiotics after birth	Systemic antibiotics given to child indicated in Medical Birth Registry	Yes/No	Binary	Neonatal complications are a risk factor for PPD
I.congen.ab	Congenital anomalies	Congenital anomalies indicated in Medical Birth Registry	Yes/No	Binary	Neonatal complications are a risk factor for PPD
Reproductive Factors					
N.age.menar	Age at menarche	Reported age of first menstrual period, Q1	Age in years (restricted t	Numeric	Age at menarche may suggest reproductive differences
F.pms	Premenstrual Syndrome symptoms	Reported response to - Are you usually depressed or irritable before your period?, Q1	No, A little, A lot	Ordered Factor	Premenstrual Syndrome is a risk factor for PPD and may suggest hormonal sensitivity

Supplementary Table S3. Parameters used in PRSice2

**Software reference**
PRSice 2.3.3
https://github.com/choishingwan/PRSice
Choi SW, O'Reilly PF. PRSice-2: Polygenic Risk Score Software for Biobank-Scale Data. Gigascience 8(7). 2019 July 01.

**Parameters used:**										
**bar-levels**	5.00E-08	5.00E-07	5.00E-06	5.00E-05	0.001	0.005	0.01	0.1	0.5	1
**base-info**	INFO:0.9									
**clump-kb**	250kb									
**clump-p**	1									
**clump-r2**	0.1									
**lower**	5.00E-08									
**maf**	0.01									
**x-range**	chr6:25000000-34000000									

Scores were further processed by extracting the first principal component for use in analyses.
This approach, outlined in Coombes et al. (2020) Genet. Epidemiol. (https://doi.org/10.1002/gepi.22339) is recommended to reduce overfitting.

Supplementary Table S4. Number of SNPs to calculate PGS at each p-value threshold and proportion of variance explained by PC1 of each trait

		Polygenic Score							
**ALL_SCORE heading**	**p threshold**	**ADHD**	**AN**	**ANX**	**BIP**	**MDD**	**OCD**	**PTSD**	**SCZ**
**Pt_5e.08**	5.00E-08	12	1	7	15	66	0	0	315
**Pt_5e.07**	5.00E-07	31	3	12	47	132	0	2	504
**Pt_5e.06**	5.00E-06	77	9	32	125	306	9	22	892
**Pt_5e.05**	5.00E-05	264	81	151	331	738	72	147	1773
**Pt_0.001**	0.001	1683	943	1652	1885	3647	848	1866	5191
**Pt_0.005**	0.005	4890	3511	6197	5167		3182	6799	10584
**Pt_0.01**	0.01	7937	6169	10651	7968	15391	5501	11776	14610
**Pt_0.1**	0.1	38695	36723	63939	35432	72188	33618	64507	47456
**Pt_0.5**	0.5	105919	109397	193764	92654	198965	99037	170418	106366
**Pt_1**	1	141175	147745	266667	121104	268424	131816	223134	134128
**% R^2 PC1**		**0.495**	**0.401**	**0.463**	**0.493**	**0.545**	**0.506**	**0.461**	**0.671**

Supplementary Table S5. Filtered SNPs in PGS Generation

		Base GWAS Summary Statistics File			Target Sample								
**Polygenic Score**	**Phenotype**	**Variants Observed**	**Variants Excluded**	**Variants Included**	**N**	**Males**	**Females**	**Founders**	**Variants not found**	**Variants with mismatch**	**Varinats outside of x-range**	**Variants included**	**Variants included after clumping**
ADHD	Attention Deficit Hyperactivity Disorder	6774224	1124401	5649823	207115	92317	114779	134278	1588137	0		5393611	201588
AN	Anorexia Nervosa	8219102	1267841	6951261	207409	92228	114734	134468	1644621	4		5337123	195469
ANX	Anxiety Disorder	1926782	1347856	6578926	207409	92228	114734	134468	1219161	0		5762587	266667
BIP	Bipolar Disorder	13413232	7089220	6324012	207409	92228	114734	134468	2236771	40		4744937	121104
MDD	Major Depression	8483301	1297781	7185520	207409	92468	114922	134468	1244561	1	12196	5724990	268424
OCD	Obsessive Compulsive Disoder	8409516	3061663	5347853	207409	92228	114734	134468	2207084	6		4774658	131816
PTSD	Post Traumatic Stress Disorder	13206891	13206891	5832286	207409	92228	114734	134468	4038255	100	13075	2930318	225835
SCZ	Schizophrenia	7585077	7585077	5177137	207409	92228	114734	134468	1997597	0		4984151	134128

Supplementary Table S6: GWAS Summary Statistics Sources for Psychiatric Disorders

Polygenic Score	Phenotype	Outcome Type	MAF	Clump	Reference PMID	Year	Journal	Title	Authors
ADHD	Attention Deficit Hyperactivity Disorder	binary	0.01	250_1_0.1	30478444	2019	Nature Genetics	Discovery of the first genome-wide significant risk loci for attention deficit/hyperactivity disorder	Demontis D, Walters RK, Martin J, Mattheisen M, Als TD, Agerbo E, Baldursson G, Belliveau R, Bybjerg-Grauholm J, Bækvad-Hansen M, Cerrato F, Chambert K, Churchhouse C, Dumont A, Eriksson N, Gandal M, Goldstein JI, Grasby KL, Grove J, Gudmundsson OO, Hansen CS, Hauberg ME, Hollegaard MV, Howrigan DP, Huang H, Maller JB, Martin AR, Martin NG, Moran J, Pallesen J, Palmer DS, Pedersen CB, Pedersen MG, Poterba T, Poulsen JB, Ripke S, Robinson EB, Satterstrom FK, Stefansson H, Stevens C, Turley P, Walters GB, Won H, Wright MJ; ADHD Working Group of the Psychiatric Genomics Consortium (PGC); Early Lifecourse & Genetic Epidemiology (EAGLE) Consortium; 23andMe Research Team; Andreassen OA, Asherson P, Burton CL, Boomsma DI, Cormand B, Dalsgaard S, Franke B, Gelernter J, Geschwind D, Hakonarson H, Haavik J, Kranzler HR, Kuntsi J, Langley K, Lesch KP, Middeldorp C, Reif A, Rohde LA, Roussos P, Schachar R, Sklar P, Sonuga-Barke EJS, Sullivan PF, Thapar A, Tung JY, Waldman ID, Medland SE, Stefansson K, Nordentoft M, Hougaard DM, Werge T, Mors O, Mortensen PB, Daly MJ, Faraone SV, Børglum AD, Neale BM
AN	Anorexia Nervosa	binary	0.01	250_1_0.1	31308545	2019	Nature Genetics	Genome-wide association study identifies eight risk loci and implicates metabo-psychiatric origins for anorexia nervosa	Watson HJ, Yilmaz Z, Thornton LM, Hübel C, Coleman JRI, Gaspar HA, Bryois J, Hinney A, Leppä VM, Mattheisen M, Medland SE, Ripke S, Yao S, Giusti-Rodríguez P; Anorexia Nervosa Genetics Initiative; Hanscombe KB, Purves KL; Eating Disorders Working Group of the Psychiatric Genomics Consortium; Adan RAH, Alfredsson L, Ando T, Andreassen OA, Baker JH, Berrettini WH, Boehm I, Boni C, Perica VB, Buehren K, Burghardt R, Cassina M, Cichon S, Clementi M, Cone RD, Courtet P, Crow S, Crowley JJ, Danner UN, Davis OSP, de Zwaan M, Dedoussis G, Degortes D, DeSocio JE, Dick DM, Dikeos D, Dina C, Dmitrzak-Weglarz M, Docampo E, Duncan LE, Egberts K, Ehrlich S, Escaramís G, Esko T, Estivill X, Farmer A, Favaro A, Fernández-Aranda F, Fichter MM, Fischer K, Föcker M, Foretova L, Forstner AJ, Forzan M, Franklin CS, Gallinger S, Giegling I, Giuranna J, Gonidakis F, Gorwood P, Mayora MG, Guillaume S, Guo Y, Hakonarson H, Hatzikotoulas K, Hauser J, Hebebrand J, Helder SG, Herms S, Herpertz-Dahlmann B, Herzog W, Huckins LM, Hudson JI, Imgart H, Inoko H, Janout V, Jiménez-Murcia S, Julià A, Kalsi G, Kaminská D, Kaprio J, Karhunen L, Karwautz A, Kas MJH, Kennedy JL, Keski-Rahkonen A, Kiezebrink K, Kim YR, Klareskog L, Klump KL, Knudsen GPS, La Via MC, Le Hellard S, Levitan RD, Li D, Lilenfeld L, Lin BD, Lissowska J, Luykx J, Magistretti PJ, Maj M, Mannik K, Marsal S, Marshall CR, Mattingsdal M, McDevitt S, McGuffin P, Metspalu A, Meulenbelt I, Micali N, Mitchell K, Monteleone AM, Monteleone P, Munn-Chernoff MA, Nacmias B, Navratilova M, Ntalla I, O'Toole JK, Ophoff RA, Padyukov L, Palotie A, Pantel J, Papezova H, Pinto D, Rabionet R, Raevuori A, Ramoz N, Reichborn-Kjennerud T, Ricca V, Ripatti S, Ritschel F, Roberts M, Rotondo A, Rujescu D, Rybakowski F, Santonastaso P, Scherag A, Scherer SW, Schmidt U, Schork NJ, Schosser A, Seitz J, Slachtova L, Slagboom PE, Slof-Op 't Landt MCT, Slopien A, Sorbi S, Świątkowska B, Szatkiewicz JP, Tachmazidou I, Tenconi E, Tortorella A, Tozzi F, Treasure J, Tsitsika A, Tyszkiewicz-Nwafor M, Tziouvas K, van Elburg AA, van Furth EF, Wagner G, Walton E, Widen E, Zeggini E, Zerwas S, Zipfel S, Bergen AW, Boden JM, Brandt H, Crawford S, Halmi KA, Horwood LJ, Johnson C, Kaplan AS, Kaye WH, Mitchell JE, Olsen CM, Pearson JF, Pedersen NL, Strober M, Werge T, Whiteman DC, Woodside DB, Stuber GD, Gordon S, Grove J, Henders AK, Juréus A, Kirk KM, Larsen JT, Parker R, Petersen L, Jordan J, Kennedy M, Montgomery GW, Wade TD, Birgegård A, Lichtenstein P, Norring C, Landén M, Martin NG, Mortensen PB, Sullivan PF, Breen G, Bulik CM
ANX	Anxiety Disorder	binary	0.01	250_1_0.1	31748690	2020	Molecular Psychiatry	A major role for common genetic variation in anxiety disorders	Purves KL, Coleman JRI, Meier SM, Rayner C, Davis KAS, Cheesman R, Bækvad-Hansen M, Børglum AD, Wan Cho S, Jürgen Deckert J, Gaspar HA, Bybjerg-Grauholm J, Hettema JM, Hotopf M, Hougaard D, Hübel C, Kan C, McIntosh AM, Mors O, Bo Mortensen P, Nordentoft M, Werge T, Nicodemus KK, Mattheisen M, Breen G, Eley TC
BIP	Bipolar Disorder	binary	0.01	250_1_0.1	34002096	2021	Nature Genetics	Genome-wide association study of more than 40,000 bipolar disorder cases provides new insights into the underlying biology	Mullins N, Forstner AJ, O'Connell KS, Coombes B, Coleman JRI, Qiao Z, Als TD, Bigdeli TB, Børte S, Bryois J, Charney AW, Drange OK, Gandal MJ, Hagenaars SP, Ikeda M, Kamitaki N, Kim M, Krebs K, Panagiotaropoulou G, Schilder BM, Sloofman LG, Steinberg S, Trubetskoy V, Winsvold BS, Won HH, Abramova L, Adorjan K, Agerbo E, Al Eissa M, Albani D, Alliey-Rodriguez N, Anjorin A, Antilla V, Antoniou A, Awasthi S, Baek JH, Bækvad-Hansen M, Bass N, Bauer M, Beins EC, Bergen SE, Birner A, Bøcker Pedersen C, Bøen E, Boks MP, Bosch R, Brum M, Brumpton BM, Brunkhorst-Kanaan N, Budde M, Bybjerg-Grauholm J, Byerley W, Cairns M, Casas M, Cervantes P, Clarke TK, Cruceanu C, Cuellar-Barboza A, Cunningham J, Curtis D, Czerski PM, Dale AM, Dalkner N, David FS, Degenhardt F, Djurovic S, Dobbyn AL, Douzenis A, Elvsåshagen T, Escott-Price V, Ferrier IN, Fiorentino A, Foroud TM, Forty L, Frank J, Frei O, Freimer NB, Frisén L, Gade K, Garnham J, Gelernter J, Giørtz Pedersen M, Gizer IR, Gordon SD, Gordon-Smith K, Greenwood TA, Grove J, Guzman-Parra J, Ha K, Haraldsson M, Hautzinger M, Heilbronner U, Hellgren D, Herms S, Hoffmann P, Holmans PA, Huckins L, Jamain S, Johnson JS, Kalman JL, Kamatani Y, Kennedy JL, Kittel-Schneider S, Knowles JA, Kogevinas M, Koromina M, Kranz TM, Kranzler HR, Kubo M, Kupka R, Kushner SA, Lavebratt C, Lawrence J, Leber M, Lee HJ, Lee PH, Levy SE, Lewis C, Liao C, Lucae S, Lundberg M, MacIntyre DJ, Magnusson SH, Maier W, Maihofer A, Malaspina D, Maratou E, Martinsson L, Mattheisen M, McCarroll SA, McGregor NW, McGuffin P, McKay JD, Medeiros H, Medland SE, Millischer V, Montgomery GW, Moran JL, Morris DW, Mühleisen TW, O'Brien N, O'Donovan C, Olde Loohuis LM, Oruc L, Papiol S, Pardiñas AF, Perry A, Pfennig A, Porichi E, Potash JB, Quested D, Raj T, Rapaport MH, DePaulo JR, Regeer EJ, Rice JP, Rivas F, Rivera M, Roth J, Roussos P, Ruderfer DM, Sánchez-Mora C, Schulte EC, Senner F, Sharp S, Shilling PD, Sigurdsson E, Sirignano L, Slaney C, Smeland OB, Smith DJ, Sobell JL, Søholm Hansen C, Soler Artigas M, Spijker AT, Stein DJ, Strauss JS, Świątkowska B, Terao C, Thorgeirsson TE, Toma C, Tooney P, Tsermpini EE, Vawter MP, Vedder H, Walters JTR, Witt SH, Xi S, Xu W, Yang JMK, Young AH, Young H, Zandi PP, Zhou H, Zillich L; HUNT All-In Psychiatry; Adolfsson R, Agartz I, Alda M, Alfredsson L, Babadjanova G, Backlund L, Baune BT, Bellivier F, Bengesser S, Berrettini WH, Blackwood DHR, Boehnke M, Børglum AD, Breen G, Carr VJ, Catts S, Corvin A, Craddock N, Dannlowski U, Dikeos D, Esko T, Etain B, Ferentinos P, Frye M, Fullerton JM, Gawlik M, Gershon ES, Goes FS, Green MJ, Grigoroiu-Serbanescu M, Hauser J, Henskens F, Hillert J, Hong KS, Hougaard DM, Hultman CM, Hveem K, Iwata N, Jablensky AV, Jones I, Jones LA, Kahn RS, Kelsoe JR, Kirov G, Landén M, Leboyer M, Lewis CM, Li QS, Lissowska J, Lochner C, Loughland C, Martin NG, Mathews CA, Mayoral F, McElroy SL, McIntosh AM, McMahon FJ, Melle I, Michie P, Milani L, Mitchell PB, Morken G, Mors O, Mortensen PB, Mowry B, Müller-Myhsok B, Myers RM, Neale BM, Nievergelt CM, Nordentoft M, Nöthen MM, O'Donovan MC, Oedegaard KJ, Olsson T, Owen MJ, Paciga SA, Pantelis C, Pato C, Pato MT, Patrinos GP, Perlis RH, Posthuma D, Ramos-Quiroga JA, Reif A, Reininghaus EZ, Ribasés M, Rietschel M, Ripke S, Rouleau GA, Saito T, Schall U, Schalling M, Schofield PR, Schulze TG, Scott LJ, Scott RJ, Serretti A, Shannon Weickert C, Smoller JW, Stefansson H, Stefansson K, Stordal E, Streit F, Sullivan PF, Turecki G, Vaaler AE, Vieta E, Vincent JB, Waldman ID, Weickert TW, Werge T, Wray NR

MDD	Major Depression	binary	0.01	250_1_0.1	30718901	2019	Nature Neuroscience	Genome-wide meta-analysis of depression identifies 102 independent variants and highlights the importance of the prefrontal brain regions	Howard DM, Adams MJ, Clarke TK, Hafferty JD, Gibson J, Shirali M, Coleman JRI, Hagenaars SP, Ward J, Wigmore EM, Alloza C, Shen X, Barbu MC, Xu EY, Whalley HC, Marioni RE, Porteous DJ, Davies G, Deary IJ, Hemani G, Berger K, Teismann H, Rawal R, Arolt V, Baune BT, Dannlowski U, Domschke K, Tian C, Hinds DA; 23andMe Research Team; Major Depressive Disorder Working Group of the Psychiatric Genomics Consortium; Trzaskowski M, Byrne EM, Ripke S, Smith DJ, Sullivan PF, Wray NR, Breen G, Lewis CM, McIntosh AM
OCD	Obsessive Compulsive Disoder	binary	0.01	250_1_0.1	28761083	2018	Molecular Psychiatry	Revealing the complex genetic architecture of obsessive-compulsive disorder using meta-analysis	International Obsessive Compulsive Disorder Foundation Genetics Collaborative (IOCDF-GC) and OCD Collaborative Genetics Association Studies (OCGAS)
PTSD	Post Traumatic Stress Disorder	binary	0.01	250_1_0.1	31594949	2019	Nature Communications	International meta-analysis of PTSD genome-wide association studies identifies sex- and ancestry-specific genetic risk loci	Nievergelt CM, Maihofer AX, Klengel T, Atkinson EG, Chen CY, Choi KW, Coleman JRI, Dalvie S, Duncan LE, Gelernter J, Levey DF, Logue MW, Polimanti R, Provost AC, Ratanatharathorn A, Stein MB, Torres K, Aiello AE, Almli LM, Amstadter AB, Andersen SB, Andreassen OA, Arbisi PA, Ashley-Koch AE, Austin SB, Avdibegovic E, Babić D, Bækvad-Hansen M, Baker DG, Beckham JC, Bierut LJ, Bisson JI, Boks MP, Bolger EA, Børglum AD, Bradley B, Brashear M, Breen G, Bryant RA, Bustamante AC, Bybjerg-Grauholm J, Calabrese JR, Caldas-de-Almeida JM, Dale AM, Daly MJ, Daskalakis NP, Deckert J, Delahanty DL, Dennis MF, Disner SG, Domschke K, Dzubur-Kulenovic A, Erbes CR, Evans A, Farrer LA, Feeny NC, Flory JD, Forbes D, Franz CE, Galea S, Garrett ME, Gelaye B, Geuze E, Gillespie C, Uka AG, Gordon SD, Guffanti G, Hammamieh R, Harnal S, Hauser MA, Heath AC, Hemmings SMJ, Hougaard DM, Jakovljevic M, Jett M, Johnson EO, Jones I, Jovanovic T, Qin XJ, Junglen AG, Karstoft KI, Kaufman ML, Kessler RC, Khan A, Kimbrel NA, King AP, Koen N, Kranzler HR, Kremen WS, Lawford BR, Lebois LAM, Lewis CE, Linnstaedt SD, Lori A, Lugonja B, Luykx JJ, Lyons MJ, Maples-Keller J, Marmar C, Martin AR, Martin NG, Maurer D, Mavissakalian MR, McFarlane A, McGlinchey RE, McLaughlin KA, McLean SA, McLeay S, Mehta D, Milberg WP, Miller MW, Morey RA, Morris CP, Mors O, Mortensen PB, Neale BM, Nelson EC, Nordentoft M, Norman SB, O'Donnell M, Orcutt HK, Panizzon MS, Peters ES, Peterson AL, Peverill M, Pietrzak RH, Polusny MA, Rice JP, Ripke S, Risbrough VB, Roberts AL, Rothbaum AO, Rothbaum BO, Roy-Byrne P, Ruggiero K, Rung A, Rutten BPF, Saccone NL, Sanchez SE, Schijven D, Seedat S, Seligowski AV, Seng JS, Sheerin CM, Silove D, Smith AK, Smoller JW, Sponheim SR, Stein DJ, Stevens JS, Sumner JA, Teicher MH, Thompson WK, Trapido E, Uddin M, Ursano RJ, van den Heuvel LL, Van Hooff M, Vermetten E, Vinkers CH, Voisey J, Wang Y, Wang Z, Werge T, Williams MA, Williamson DE, Winternitz S, Wolf C, Wolf EJ, Wolff JD, Yehuda R, Young RM, Young KA, Zhao H, Zoellner LA, Liberzon I, Ressler KJ, Haas M, Koenen KC
SCZ	Schizophrenia	binary	0.01	250_1_0.1	35396580	2022	Nature	Mapping genomic loci implicates genes and synaptic biology in schizophrenia	Trubetskoy V, Pardiñas AF, Qi T, Panagiotaropoulou G, Awasthi S, Bigdeli TB, Bryois J, Chen CY, Dennison CA, Hall LS, Lam M, Watanabe K, Frei O, Ge T, Harwood JC, Koopmans F, Magnusson S, Richards AL, Sidorenko J, Wu Y, Zeng J, Grove J, Kim M, Li Z, Voloudakis G, Zhang W, Adams M, Agartz I, Atkinson EG, Agerbo E, Al Eissa M, Albus M, Alexander M, Alizadeh BZ, Alptekin K, Als TD, Amin F, Arolt V, Arrojo M, Athanasiu L, Azevedo MH, Bacanu SA, Bass NJ, Begemann M, Belliveau RA, Bene J, Benyamin B, Bergen SE, Blasi G, Bobes J, Bonassi S, Braun A, Bressan RA, Bromet EJ, Bruggeman R, Buckley PF, Buckner RL, Bybjerg-Grauholm J, Cahn W, Cairns MJ, Calkins ME, Carr VJ, Castle D, Catts SV, Chambert KD, Chan RCK, Chaumette B, Cheng W, Cheung EFC, Chong SA, Cohen D, Consoli A, Cordeiro Q, Costas J, Curtis C, Davidson M, Davis KL, de Haan L, Degenhardt F, DeLisi LE, Demontis D, Dickerson F, Dikeos D, Dinan T, Djurovic S, Duan J, Ducci G, Dudbridge F, Eriksson JG, Fañanás L, Faraone SV, Fiorentino A, Forstner A, Frank J, Freimer NB, Fromer M, Frustaci A, Gadelha A, Genovese G, Gershon ES, Giannitelli M, Giegling I, Giusti-Rodríguez P, Godard S, Goldstein JI, González Peñas J, González-Pinto A, Gopal S, Gratten J, Green MF, Greenwood TA, Guillin O, Gülöksüz S, Gur RE, Gur RC, Gutiérrez B, Hahn E, Hakonarson H, Haroutunian V, Hartmann AM, Harvey C, Hayward C, Henskens FA, Herms S, Hoffmann P, Howrigan DP, Ikeda M, Iyegbe C, Joa I, Julià A, Kähler AK, Kam-Thong T, Kamatani Y, Karachanak-Yankova S, Kebir O, Keller MC, Kelly BJ, Khrunin A, Kim SW, Klovins J, Kondratiev N, Konte B, Kraft J, Kubo M, Kučinskas V, Kučinskiene ZA, Kusumawardhani A, Kuzelova-Ptackova H, Landi S, Lazzeroni LC, Lee PH, Legge SE, Lehrer DS, Lencer R, Lerer B, Li M, Lieberman J, Light GA, Limborska S, Liu CM, Lönnqvist J, Loughland CM, Lubinski J, Luykx JJ, Lynham A, Macek M Jr, Mackinnon A, Magnusson PKE, Maher BS, Maier W, Malaspina D, Mallet J, Marder SR, Marsal S, Martin AR, Martorell L, Mattheisen M, McCarley RW, McDonald C, McGrath JJ, Medeiros H, Meier S, Melegh B, Melle I, Mesholam-Gately RI, Metspalu A, Michie PT, Milani L, Milanova V, Mitjans M, Molden E, Molina E, Molto MD, Mondelli V, Moreno C, Morley CP, Muntané G, Murphy KC, Myin-Germeys I, Nenadić I, Nestadt G, Nikitina-Zake L, Noto C, Nuechterlein KH, O'Brien NL, O'Neill FA, Oh SY, Olincy A, Ota VK, Pantelis C, Papadimitriou GN, Parellada M, Paunio T, Pellegrino R, Periyasamy S, Perkins DO, Pfuhlmann B, Pietiläinen O, Pimm J, Porteous D, Powell J, Quattrone D, Quested D, Radant AD, Rampino A, Rapaport MH, Rautanen A, Reichenberg A, Roe C, Roffman JL, Roth J, Rothermundt M, Rutten BPF, Saker-Delye S, Salomaa V, Sanjuan J, Santoro ML, Savitz A, Schall U, Scott RJ, Seidman LJ, Sharp SI, Shi J, Siever LJ, Sigurdsson E, Sim K, Skarabis N, Slominsky P, So HC, Sobell JL, Söderman E, Stain HJ, Steen NE, Steixner-Kumar AA, Stögmann E, Stone WS, Straub RE, Streit F, Strengman E, Stroup TS, Subramaniam M, Sugar CA, Suvisaari J, Svrakic DM, Swerdlow NR, Szatkiewicz JP, Ta TMT, Takahashi A, Terao C, Thibaut F, Toncheva D, Tooney PA, Torretta S, Tosato S, Tura GB, Turetsky BI, Üçok A, Vaaler A, van Amelsvoort T, van Winkel R, Veijola J, Waddington J, Walter H, Waterreus A, Webb BT, Weiser M, Williams NM, Witt SH, Wormley BK, Wu JQ, Xu Z, Yolken R, Zai CC, Zhou W, Zhu F, Zimprich F, Atbaşoğlu EC, Ayub M, Benner C, Bertolino A, Black DW, Bray NJ, Breen G, Buccola NG, Byerley WF, Chen WJ, Cloninger CR, Crespo-Facorro B, Donohoe G, Freedman R, Galletly C, Gandal MJ, Gennarelli M, Hougaard DM, Hwu HG, Jablensky AV, McCarroll SA, Moran JL, Mors O, Mortensen PB, Müller-Myhsok B, Neil AL, Nordentoft M, Pato MT, Petryshen TL, Pirinen M, Pulver AE, Schulze TG, Silverman JM, Smoller JW, Stahl EA, Tsuang DW, Vilella E, Wang SH, Xu S; Indonesia Schizophrenia Consortium; PsychENCODE; Psychosis Endophenotypes International Consortium; SynGO Consortium; Adolfsson R, Arango C, Baune BT, Belangero SI, Børglum AD, Braff D, Bramon E, Buxbaum JD, Campion D, Cervilla JA, Cichon S, Collier DA, Corvin A, Curtis D, Forti MD, Domenici E, Ehrenreich H, Escott-Price V, Esko T, Fanous AH, Gareeva A, Gawlik M, Gejman PV, Gill M, Glatt SJ, Golimbet V, Hong KS, Hultman CM, Hyman SE, Iwata N, Jönsson EG, Kahn RS, Kennedy JL, Khusnutdinova E, Kirov G, Knowles JA, Krebs MO, Laurent-Levinson C, Lee J, Lencz T, Levinson DF, Li QS, Liu J, Malhotra AK, Malhotra D, McIntosh A, McQuillin A, Menezes PR, Morgan VA, Morris DW, Mowry BJ, Murray RM, Nimgaonkar V, Nöthen MM, Ophoff RA, Paciga SA, Palotie A, Pato CN, Qin S, Rietschel M, Riley BP, Rivera M, Rujescu D, Saka MC, Sanders AR, Schwab SG, Serretti A, Sham PC, Shi Y, St Clair D, Stefánsson H, Stefansson K, Tsuang MT, van Os J, Vawter MP, Weinberger DR, Werge T, Wildenauer DB, Yu X, Yue W, Holmans PA,

Supplementary Table S7: Summary statistics of feature variables used in clustering algorithm by cluster

Table S7a. Summary of feature variables in training set

Characteristic	Overall, N =	0, N = 69	1, N = 1,483	2, N = 608	3, N = 144	4, N = 1,483	5, N = 498	6, N = 307	7, N = 310	8, N = 112	9, N = 225	p-value	q-value
EPDS score, mean (SD)	9.91 (2.12)	9.93 (1.77)	10.21 (2.21)	9.58 (2.07)	9.19 (1.47)	10.07 (2.17)	9.61 (2.11)	10.00 (2.08)	10.01 (2.11)	9.40 (1.84)	8.93 (1.21)	<0.0001	<0.0001
DES-anger score, mean (SD)	5.35 (2.27)	6.08 (2.35)	5.67 (2.36)	5.09 (2.08)	4.92 (2.26)	5.40 (2.23)	4.76 (2.21)	5.72 (2.09)	5.50 (2.26)	4.82 (2.37)	4.64 (2.05)	<0.0001	<0.0001
Hopkins-anxiety score, median (IQR)	0.50 (0.25, 1.00)	0.50 (0.25, 1.00)	0.75 (0.25, 1.00)	0.50 (0.25, 0.75)	0.25 (0.00, 0.75)	0.75 (0.25, 1.00)	0.50 (0.25, 0.75)	0.50 (0.25, 1.00)	0.50 (0.25, 0.75)	0.50 (0.25, 0.75)	0.25 (0.00, 0.50)	<0.0001	<0.0001
Medication-depression, n (%)	278 (5.3%)	0 (0.0%)	144 (9.7%)	0 (0.0%)	0 (0.0%)	133 (9.0%)	0 (0.0%)	≤5 (≤2.0%)	0 (0.0%)	0 (0.0%)	0 (0.0%)	0.00050	<0.0001
Medication-anxiety, n (%)	154 (2.9%)	0 (0.0%)	70 (4.7%)	0 (0.0%)	0 (0.0%)	84 (5.7%)	0 (0.0%)	0 (0.0%)	0 (0.0%)	0 (0.0%)	0 (0.0%)	0.00050	<0.0001
Depression hx before pregnancy, n (%)	945 (18%)	≤5 (≤8.0%)	463 (31.2%)	0 (0.0%)	0 (0.0%)	476 (32.1%)	0 (0.0%)	0 (0.0%)	0 (0.0%)	5 (4.5%)	0 (0.0%)	<0.0001	<0.0001
Depressive symptoms, 1st trimester, n (%)	595 (11.4%)	≤5 (≤8.0%)	273 (18.4%)	0 (0.0%)	0 (0.0%)	315 (21.2%)	0 (0.0%)	≤5 (≤2.0%)	0 (0.0%)	≤5 (≤5.0%)	0 (0.0%)	<0.0001	<0.0001
Depressive symptoms, 3rd trimester, n (%)	760 (14.5%)	≤5 (≤8.0%)	343 (23.1%)	0 (0.0%)	0 (0.0%)	409 (27.6%)	0 (0.0%)	0 (0.0%)	0 (0.0%)	≤5 (≤5.0%)	0 (0.0%)	<0.0001	<0.0001
Depressive symptoms, early postpartum, n (%)	1,845 (35.2%)	45 (65.2%)	612 (41.3%)	0 (0.0%)	0 (0.0%)	571 (38.5%)	0 (0.0%)	307 (100.0%)	310 (100.0%)	0 (0.0%)	0 (0.0%)	<0.0001	<0.0001
History of eating disorder, n (%)	346 (6.6%)	11 (15.9%)	165 (11.1%)	0 (0.0%)	0 (0.0%)	163 (11.0%)	0 (0.0%)	0 (0.0%)	0 (0.0%)	7 (6.2%)	0 (0.0%)	0.00050	<0.0001
History of anxiety, n (%)	620 (11.8%)	≤5 (≤8.0%)	268 (18.1%)	0 (0.0%)	0 (0.0%)	347 (23.4%)	0 (0.0%)	0 (0.0%)	0 (0.0%)	≤5 (≤5.0%)	0 (0.0%)	<0.0001	<0.0001
Consumed alcohol in pregnancy, n (%)	574 (11%)	60 (87.0%)	194 (13.1%)	0 (0.0%)	0 (0.0%)	208 (14.0%)	0 (0.0%)	0 (0.0%)	0 (0.0%)	112 (100.0%)	0 (0.0%)	<0.0001	<0.0001
Smoked in pregnancy, n (%)	1,016 (19.4%)	35 (50.7%)	376 (25.4%)	0 (0.0%)	0 (0.0%)	605 (40.8%)	0 (0.0%)	0 (0.0%)	0 (0.0%)	0 (0.0%)	0 (0.0%)	<0.0001	<0.0001
Adverse Life Events, 1st trimester and year prior, median	1.00 (0.00, 2.00)	1.00 (0.00, 2.00)	2.00 (1.00, 3.00)	1.00 (0.00, 2.00)	1.00 (0.00, 2.00)	2.00 (1.00, 2.40)	1.00 (0.00, 2.00)	1.00 (0.00, 2.00)	1.00 (0.00, 2.00)	1.00 (0.00, 2.00)	1.00 (0.00, 2.00)	<0.0001	<0.0001
History of physical abuse, n (%)	913 (17.4%)	0 (0.0%)	913 (61.6%)	0 (0.0%)	0 (0.0%)	0 (0.0%)	0 (0.0%)	0 (0.0%)	0 (0.0%)	0 (0.0%)	0 (0.0%)	<0.0001	<0.0001
History of sexual abuse, n (%)	793 (15.1%)	0 (0.0%)	793 (53.5%)	0 (0.0%)	0 (0.0%)	0 (0.0%)	0 (0.0%)	0 (0.0%)	0 (0.0%)	0 (0.0%)	0 (0.0%)	<0.0001	<0.0001
History of abuse in childhood, n (%)	1,007 (19.2%)	0 (0.0%)	1,007 (67.9%)	0 (0.0%)	0 (0.0%)	0 (0.0%)	0 (0.0%)	0 (0.0%)	0 (0.0%)	0 (0.0%)	0 (0.0%)	<0.0001	<0.0001
History of abuse in adulthood, n (%)	1,046 (20.0%)	0 (0.0%)	1,046 (70.5%)	0 (0.0%)	0 (0.0%)	0 (0.0%)	0 (0.0%)	0 (0.0%)	0 (0.0%)	0 (0.0%)	0 (0.0%)	<0.0001	<0.0001
Physical health (self-reported), n (%)												0.00050	<0.0001
Very good	1,123 (21.4%)	16 (23.2%)	269 (18.1%)	11 (1.8%)	144 (100%)	290 (19.6%)	10 (2%)	86 (28%)	44 (14.2%)	28 (25%)	225 (100%)		
Good	3,564 (68%)	46 (66.7%)	989 (66.7%)	530 (87.2%)	0 (0.0%)	1,037 (69.9%)	457 (91.8%)	201 (65.5%)	225 (72.6%)	79 (70.5%)	0 (0.0%)		
Poor	503 (9.6%)	7 (10.1%)	204 (13.8%)	62 (10.2%)	0 (0.0%)	142 (9.6%)	28 (5.6%)	19 (6.2%)	36 (11.6%)	≤5 (≤5.0%)	0 (0.0%)		
Very Poor	49 (0.9%)	0 (0.0%)	21 (1.4%)	≤5 (≤1%)	0 (0.0%)	14 (0.9%)	≤5 (≤1%)	≤5 (≤2%)	≤5 (≤2.0%)	0 (0.0%)	0 (0.0%)		
Severe pain in pregnancy, n (%)	2,773 (52.9%)	69 (100%)	846 (57%)	608 (100%)	144 (100%)	796 (53.7%)	0 (0.0%)	0 (0.0%)	310 (100.0%)	0 (0.0%)	0 (0.0%)	<0.0001	<0.0001
Maternal length of hospital stay after birth, median (IQR)	4.00 (1.00, 5.00)	3.00 (3.00, 4.40)	4.00 (3.00, 5.00)	3.00 (3.00, 4.40)	4.00 (3.00, 5.00)	4.00 (3.00, 5.00)	3.00 (3.00, 4.18)	4.00 (3.00, 5.00)	4.00 (3.00, 5.00)	3.00 (3.00, 4.50)	3.00 (3.00, 4.00)	<0.0001	<0.0001

Supplementary Table S7: Summary statistics of feature variables used in clustering algorithm by cluster

Table S7b. Summary of feature variables in replication set

Characteristic	Overall, N =	0, N = 177	1, N = 729	2, N = 323	3, NULL	4, N = 693	5, N = 263	6, N = 158	7, N = 178	8, NULL	9, N = 99	p-value	q-value
EPDS score, mean (SD)	9.88 (2.07)	9.60 (1.84)	10.20 (2.19)	9.50 (2.07)		10.13 (2.12)	9.57 (1.96)	9.69 (1.86)	9.75 (1.91)		9.00 (1.32)	<0.0001	<0.0001
DES-anger score, mean (SD)	5.36 (2.27)	5.02 (2.39)	5.61 (2.34)	5.05 (2.07)		5.58 (2.22)	4.90 (2.20)	5.29 (2.28)	5.31 (2.32)		5.11 (2.15)	<0.0001	<0.0001
Hopkins-anxiety score, median (IQR)	0.50 (0.25, 1.00)	0.50 (0.25, 0.75)	0.75 (0.25, 1.25)	0.50 (0.25, 0.75)		0.75 (0.25, 1.25)	0.50 (0.25, 0.75)	0.50 (0.25, 1.00)	0.50 (0.25, 1.00)		0.50 (0.25, 0.63)	<0.0001	<0.0001
Medication-depression, n (%)	150 (5.7%)	≤5 (≤3.0%)	67 (9.2%)	0 (0.0%)		81 (11.7%)	≤5 (≤1.9%)	0 (0.0%)	0 (0.0%)		0 (0.0%)	<0.0001	<0.0001
Medication-anxiety, n (%)	85 (3.2%)	0 (0.0%)	35 (4.8%)	0 (0.0%)		50 (7.2%)	0 (0.0%)	0 (0.0%)	0 (0.0%)		0 (0.0%)	0.00050	<0.0001
Depression hx before pregnancy, n (%)	471 (18.0%)	6 (3.4%)	215 (29.5%)	≤5 (≤2.0%)		245 (35.4%)	≤5 (≤1.9%)	≤5 (≤3.2%)	≤5 (≤2.8%)		0 (0.0%)	<0.0001	<0.0001
Depressive symptoms, 1st trimester, n (%)	287 (11.0%)	≤5 (≤3.0%)	128 (187.6%)	0 (0.0%)		149 (21.5%)	0 (0.0%)	≤5 (≤3.2%)	0 (0.0%)		≤5 (≤5.0%)	<0.0001	<0.0001
Depressive symptoms, 3rd trimester, n (%)	377 (14.4%)	≤5 (≤3.0%)	167 (22.9%)	0 (0.0%)		206 (29.7%)	0 (0.0%)	0 (0.0%)	≤5 (≤2.8%)		0 (0.0%)	<0.0001	<0.0001
Depressive symptoms, early postpartum, n (%)	970 (37.0%)	21 (11.9%)	294 (40.3%)	0 (0.0%)		319 (46.0%)	0 (0.0%)	158 (100.0%)	178 (100.0%)		0 (0.0%)	<0.0001	<0.0001
History of eating disorder, n (%)	178 (6.8%)	12 (6.8%)	83 (114%)	≤5 (≤2.0%)		77 (11.1%)	0 (0.0%)	≤5 (≤3.2%)	≤5 (≤2.8%)		≤5 (≤5.0%)	<0.0001	<0.0001
History of anxiety, n (%)	301 (11.5%)	≤5 (≤3.0%)	131 (18.0%)	0 (0.0%)		165 (23.8%)	0 (0.0%)	0 (0.0%)	≤5 (≤2.8%)		0 (0.0%)	<0.0001	<0.0001
Consumed alcohol in pregnancy, n (%)	261 (10.0%)	109 (61.6%)	87 (11.9%)	0 (0.0%)		65 (9.4%)	0 (0.0%)	0 (0.0%)	0 (0.0%)		0 (0.0%)	<0.0001	<0.0001
Smoked in pregnancy, n (%)	526 (20.1%)	16 (9.0%)	215 (29.5%)	0 (0.0%)		295 (42.6%)	0 (0.0%)	0 (0.0%)	0 (0.0%)		0 (0.0%)	<0.0001	<0.0001
Adverse Life Events, 1st trimester and year prior, median	1.00 (0.50, 2.00)	1.00 (0.00, 2.00)	2.00 (1.00, 3.00)	1.00 (0.00, 2.00)		1.60 (1.00, 3.00)	1.00 (0.00, 2.00)	1.00 (0.00, 2.00)	1.00 (0.93, 2.00)		1.00 (0.00, 2.00)	<0.0001	<0.0001
History of physical abuse, n (%)	445 (17.0%)	0 (0.0%)	444 (60.9%)	0 (0.0%)		≤5 (≤1.0%)	0 (0.0%)	0 (0.0%)	0 (0.0%)		0 (0.0%)	<0.0001	<0.0001
History of sexual abuse, n (%)	411 (15.7%)	≤5 (≤3.0%)	408 (56.0%)	0 (0.0%)		≤5 (≤1.0%)	0 (0.0%)	0 (0.0%)	0 (0.0%)		0 (0.0%)	<0.0001	<0.0001
History of abuse in childhood, n (%)	493 (18.8%)	0 (0.0%)	492 (67.5%)	0 (0.0%)		≤5 (≤1.0%)	0 (0.0%)	0 (0.0%)	0 (0.0%)		0 (0.0%)	<0.0001	<0.0001
History of abuse in adulthood, n (%)	535 (20.4%)	≤5 (≤3.0%)	532 (73.0%)	0 (0.0%)		≤5 (≤1.0%)	0 (0.0%)	0 (0.0%)	0 (0.0%)		0 (0.0%)	<0.0001	<0.0001
Physical health (self-reported), n (%)												0.00050	0.0010
Very good	515 (19.7%)	92 (52.0%)	121 (16.6%)	≤5 (≤2.0%)		131 (18.9%)	6 (2.3%)	33 (20.9%)	29 (16.3%)		99 (100.0%)		
Good	1,836 (70.1%)	76 (42.9%)	515 (70.6%)	290 (89.8%)		473 (68.3%)	244 (92.8%)	105 (66.5%)	133 (74.7%)		0 (0.0%)		
Poor	254 (9.7%)	8 (4.5%)	88 (12.1%)	27 (8.4%)		83 (12.0%)	12 (4.6%)	20 (12.7%)	16 (9.0%)		0 (0.0%)		
Very Poor	15 (0.6%)	≤5 (≤3.0%)	≤5 (≤1%)	≤5 (≤2.0%)		6 (0.9%)	≤5 (≤1.9%)	0 (0.0%)	0 (0.0%)		0 (0.0%)		
Severe pain in pregnancy, n (%)	1,455 (55.5%)	127 (71.8%)	449 (61.6%)	323 (100.0%)		378 (54.5%)	0 (0.0%)	0 (0.0%)	178 (100.0%)		0 (0.0%)	<0.0001	<0.0001
Maternal length of hospital stay after birth, median (IQR)	3.99 (3.00, 4.90)	3.00 (3.00, 5.00)	3.00 (3.00, 5.00)	3.00 (2.80, 4.50)		4.00 (3.00, 4.70)	3.00 (3.00, 4.00)	4.00 (3.00, 5.00)	4.00 (3.00, 5.00)		3.00 (2.85, 4.00)	0.0020	0.0030

Characteristic	Full test set	Unclustered	Cluster 1: chronic major depression + trauma	Cluster 2: PPD + pain	Cluster 3: mild PPD + pain	Cluster 4: chronic major depression	Cluster 5: mild PPD	Cluster 6: early-onset PPD + anger/irritability	Cluster 7: early-onset PPD + pain	Cluster 8: PPD + alcohol use	Cluster 9: low risk	q-value[4]
Total N	2,620	177	729	323		693	263	158	178		99	
Maternal age (years), mean (SD)[1]	29.65 (5.07)	30.33 (4.99)	29.48 (5.34)	30.17 (4.64)		29.32 (5.31)	29.83 (4.82)	29.42 (4.56)	29.71 (4.48)		30.09 (5.11)	0.30
Region, n (%)[3]												0.50
South-East	1355 (51.7%)	99 (55.9%)	376 (51.6%)	152 (47.1%)		349 (50.4%)	138 (52.5%)	89 (56.3%)	97 (54.5%)		55 (55.6%)	
West	697 (26.6%)	43 (24.3%)	177 (24.3%)	101 (31.3%)		194 (28.0%)	68 (25.9%)	38 (24.1%)	51 (28.7%)		25 (25.3%)	
Central	383 (14.6%)	20 (11.3%)	118 (16.2%)	47 (14.6%)		98 (14.1%)	37 (14.1%)	22 (13.9%)	25 (14.0%)		16 (16.2%)	
North	185 (7.1%)	15 (8.5%)	58 (8.0%)	23 (7.1%)		52 (7.5%)	20 (7.6%)	9 (5.7%)	≤5 (≤3.0%)		≤5 (≤5.0%)	
Married/cohabitating, n (%)[2]	2440 (93.1%)	170 (96%)	655 (89.8%)	313 (96.9%)		626 (90.3%)	252 (95.8%)	153 (96.8%)	173 (97.2%)		98 (99.0%)	<0.0001
Maternal education, n (%)[2]												<0.0001
Less than high school	410 (15.6%)	10 (5.6%)	171 (23.5%)	33 (10.2%)		139 (20.1%)	23 (8.7%)	13 (8.2%)	14 (7.9%)		7 (7.1%)	
High school diploma or some college	891 (34.0%)	44 (24.9%)	271 (37.2%)	107 (33.1%)		255 (36.8%)	77 (29.3%)	44 (27.8%)	62 (34.8%)		31 (31.3%)	
College or university degree or more	1319 (50.3%)	123 (69.5%)	287 (39.4%)	183 (56.7%)		299 (43.1%)	163 (62.0%)	101 (63.9%)	102 (57.3%)		61 (61.6%)	
Birth year, n (%)[3]												0.40
1999-2000	51 (1.9%)	≤5 (≤2.8%)	7 (1.0%)	11 (3.4%)		19 (2.7%)	≤5 (≤2.0%)	≤5 (≤4.0%)	≤5 (≤3.0%)		≤5 (≤5.0%)	
2001	100 (3.8%)	10 (5.6%)	27 (3.7%)	11 (3.4%)		29 (4.2%)	12 (4.6%)	≤5 (≤4.0%)	≤5 (≤3.0%)		≤5 (≤5.0%)	
2002	224 (8.5%)	17 (9.6%)	55 (7.5%)	38 (11.8%)		60 (8.7%)	18 (6.8%)	≤5 (≤4.0%)	14 (7.9%)		≤5 (≤5.0%)	
2003	292 (11.1%)	23 (13.0%)	73 (10.0%)	34 (10.5%)		90 (13.0%)	27 (10.3%)	16 (10.1%)	21 (11.8%)		8 (8.1%)	
2004	346 (13.2%)	17 (9.6%)	111 (15.2%)	38 (11.8%)		86 (12.4%)	38 (14.4%)	13 (8.2%)	22 (12.4%)		21 (21.2%)	
2005	406 (15.5%)	33 (18.6%)	121 (16.6%)	42 (13.0%)		101 (14.6%)	43 (16.3%)	22 (13.9%)	27 (15.2%)		17 (17.2%)	
2006	444 (16.9%)	31 (17.5%)	131 (18.0%)	54 (16.7%)		120 (17.3%)	40 (15.2%)	32 (20.3%)	26 (14.6%)		10 (10.1%)	
2007	386 (14.7%)	25 (14.1%)	102 (14.0%)	51 (15.8%)		95 (13.7%)	40 (15.2%)	29 (18.4%)	28 (15.7%)		16 (16.2%)	
2008	297 (11.3%)	15 (8.5%)	86 (11.8%)	36 (11.1%)		76 (11.0%)	29 (11.0%)	18 (11.4%)	22 (12.4%)		15 (15.2%)	
2009	74 (2.8%)	≤5 (≤2.8%)	16 (2.2%)	8 (2.5%)		17 (2.5%)	12 (4.6%)	≤5 (≤4.0%)	8 (4.5%)		≤5 (≤5.0%)	
Relationship satisfaction, mean (SD)[1]	36.28 (9.57)	37.86 (8.94)	34.59 (9.99)	37.09 (8.77)		35.98 (10.01)	37.39 (8.44)	38.29 (8.65)	37.97 (8.85)		36.07 (10.46)	<0.0001
Someone for support or advice, n (%)[2]	2365 (90.3%)	163 (92.1%)	667 (91.5%)	287 (88.9%)		613 (88.5%)	240 (91.3%)	143 (90.5%)	163 (91.6%)		89 (89.9%)	0.60
Body Mass Index (kg/m^2), mean (SD)[1]	24.11 (4.06)	23.85 (3.89)	24.48 (4.34)	24.59 (4.33)		23.96 (3.96)	23.52 (3.63)	23.59 (3.78)	24.47 (3.91)		22.96 (3.09)	0.0010
Anemia, n (%)[3]	159 (6.1%)	10 (5.6%)	54 (7.4%)	23 (7.1%)		42 (6.1%)	12 (4.6%)	8 (5.1%)	8 (4.5%)		≤5 (≤5.0%)	0.40
Ovarian cyst, n (%)[2]	234 (8.9%)	12 (6.8%)	88 (12.1%)	26 (8.0%)		66 (9.5%)	11 (4.2%)	9 (5.7%)	16 (9.0%)		6 (6.1%)	0.011
Migraine, n (%)[2]	414 (15.8%)	19 (10.7%)	149 (20.4%)	45 (13.9%)		117 (16.9%)	27 (10.3%)	21 (13.3%)	26 (14.6%)		10 (10.1%)	0.0020
Pregnancy was unplanned, n (%)[2]	712 (27.2%)	48 (27.1%)	253 (34.7%)	58 (18.0%)		206 (29.7%)	55 (20.9%)	31 (19.6%)	41 (23.0%)		20 (20.2%)	<0.0001
Nausea and vomiting of pregnancy, n (%)[2]	1165 (44.5%)	76 (42.9%)	337 (46.2%)	160 (49.5%)		289 (41.7%)	112 (42.6%)	63 (39.9%)	94 (52.8%)		34 (34.3%)	0.036
Prenatal hospitalization, n (%)[2]	963 (36.8%)	49 (27.7%)	287 (39.4%)	113 (35.0%)		276 (39.8%)	83 (31.6%)	59 (37.3%)	62 (34.8%)		34 (34.3%)	0.072
Initiation of labor, n (%)[2]												0.70
Spontaneous	2036 (77.7%)	144 (81.4%)	546 (74.9%)	248 (76.8%)		551 (79.5%)	210 (79.8%)	126 (79.7%)	135 (75.8%)		76 (76.8%()	
Induction	368 (14.0%)	19 (10.7%)	110 (15.1%)	61 (18.9%)		94 (13.6%)	33 (12.5%)	19 (12.0%)	29 (16.3%)		13 (13.1%)	
Cesarean section	216 (8.2%)	14 (7.9%)	73 (10.0%)	14 (4.3%)		48 (6.9%)	20 (7.6%)	13 (8.2%)	14 (7.9%)		10 (10.1%)	
Birth felt very unsafe, n (%)[3]	127 (4.8%)	7 (4.0%)	42 (5.8%)	14 (4.3%)		33 (4.8%)	7 (2.7%)	15 (9.5%)	7 (3.9%)		≤5 (≤5.0%)	0.088
Birth complications, n (%)[2]	562 (21.5%)	31 (17.5%)	173 (23.7%)	64 (19.8%)		131 (18.9%)	55 (20.9%)	39 (24.7%)	51 (28.7%)		18 (18.2%)	0.088
Birthweight (g), mean (SD)[1]	3623.47 (522.57)	3684.86 (512.52)	3637.58 (553.47)	3662.44 (477.10)		3583.82 (496.76)	3599 (536.53)	3626.89 (504.55)	3642.96 (588.13)		3584.01 (479.36)	0.40
Apgar (1 min), mean (SD)[1]	8.68 (1.18)	8.59 (1.35)	8.72 (1.05)	8.68 (1.18)		8.72 (1.19)	8.69 (1.10)	8.49 (1.39)	8.57 (1.38)		8.88 (0.88)	0.40
Admitted to NICU, n (%)[2]	363 (13.9%)	21 (11.9%)	96 (13.2%)	35 (10.8%)		95 (13.7%)	49 (18.6%)	24 (15.2%)	28.0 (15.7%)		15 (15.2%)	0.30
Age at menarche (years), mean (SD)[1]	13.00 (1.45)	13.07 (1.34)	12.84 (1.44)	13.02 (1.45)		13.03 (1.37)	13.14 (1.65)	12.94 (1.28)	13.02 (1.45)		13.41 (1.81)	0.11
Feels very depressed or irritable before period, n (%)[2]	1060 (40.5%)	58 (32.8%)	327 (44.9%)	114 (35.3%)		316 (45.6%)	81 (30.8%)	53 (33.5%)	83 (46.6%)		28 (28.3%)	<0.0001

[1] Kruskal-Wallis rank sum test used to assess differences between clusters.

[2] Pearson's Chi-squared test used to assess difference between clusters.

[3] Fisher's Exact Test for count data with simulated p-value (based on 2000 replicates) used to assess difference between clusters.

[4] q-values reflect False Discovery Rate (FDR) adjusted p-values, calculated using the Benjamini-Hochberg procedure. A q-value of <0.05 indicates statistical significance after accounting for multiple testing.

Supplementary Table S9: Box-Tidwell tests of linearity for logistic PGS models					
PRS	Cluster	Estimate	Std.error	Statistic	p.value
ADHD	1	-0.13	0.17	-0.75	0.46
ADHD	2	-0.15	0.27	-0.57	0.57
ADHD	3	0.34	0.45	0.78	0.44
ADHD	4	-0.04	0.17	-0.22	0.83
ADHD	5	-0.26	0.31	-0.85	0.39
ADHD	6	0.76	0.31	2.47	0.01
ADHD	7	0.34	0.31	1.09	0.28
ADHD	8	-0.01	0.61	-0.02	0.98
ADHD	9	0.06	0.40	0.16	0.87
AN	1	-0.13	0.17	-0.75	0.46
AN	2	-0.15	0.27	-0.57	0.57
AN	3	0.34	0.45	0.78	0.44
AN	4	-0.04	0.17	-0.22	0.83
AN	5	-0.26	0.31	-0.85	0.39
AN	6	0.76	0.31	2.47	0.01
AN	7	0.34	0.31	1.09	0.28
AN	8	-0.01	0.61	-0.02	0.98
AN	9	0.06	0.40	0.16	0.87
ANX	1	-0.13	0.17	-0.75	0.46
ANX	2	-0.15	0.27	-0.57	0.57
ANX	3	0.34	0.45	0.78	0.44
ANX	4	-0.04	0.17	-0.22	0.83
ANX	5	-0.26	0.31	-0.85	0.39
ANX	6	0.76	0.31	2.47	0.01
ANX	7	0.34	0.31	1.09	0.28
ANX	8	-0.01	0.61	-0.02	0.98
ANX	9	0.06	0.40	0.16	0.87
BIP	1	-0.13	0.17	-0.75	0.46
BIP	2	-0.15	0.27	-0.57	0.57
BIP	3	0.34	0.45	0.78	0.44
BIP	4	-0.04	0.17	-0.22	0.83
BIP	5	-0.26	0.31	-0.85	0.39
BIP	6	0.76	0.31	2.47	0.01
BIP	7	0.34	0.31	1.09	0.28
BIP	8	-0.01	0.61	-0.02	0.98
BIP	9	0.06	0.40	0.16	0.87
MDD	1	-0.13	0.17	-0.75	0.46
MDD	2	-0.15	0.27	-0.57	0.57
MDD	3	0.34	0.45	0.78	0.44
MDD	4	-0.04	0.17	-0.22	0.83
MDD	5	-0.26	0.31	-0.85	0.39
MDD	6	0.76	0.31	2.47	0.01
MDD	7	0.34	0.31	1.09	0.28
MDD	8	-0.01	0.61	-0.02	0.98
MDD	9	0.06	0.40	0.16	0.87
OCD	1	-0.13	0.17	-0.75	0.46
OCD	2	-0.15	0.27	-0.57	0.57
OCD	3	0.34	0.45	0.78	0.44
OCD	4	-0.04	0.17	-0.22	0.83
OCD	5	-0.26	0.31	-0.85	0.39
OCD	6	0.76	0.31	2.47	0.01
OCD	7	0.34	0.31	1.09	0.28
OCD	8	-0.01	0.61	-0.02	0.98
OCD	9	0.06	0.40	0.16	0.87
PTSD	1	-0.13	0.17	-0.75	0.46
PTSD	2	-0.15	0.27	-0.57	0.57
PTSD	3	0.34	0.45	0.78	0.44
PTSD	4	-0.04	0.17	-0.22	0.83
PTSD	5	-0.26	0.31	-0.85	0.39
PTSD	6	0.76	0.31	2.47	0.01
PTSD	7	0.34	0.31	1.09	0.28
PTSD	8	-0.01	0.61	-0.02	0.98
PTSD	9	0.06	0.40	0.16	0.87
SCZ	1	-0.13	0.17	-0.75	0.46
SCZ	2	-0.15	0.27	-0.57	0.57
SCZ	3	0.34	0.45	0.78	0.44
SCZ	4	-0.04	0.17	-0.22	0.83
SCZ	5	-0.26	0.31	-0.85	0.39
SCZ	6	0.76	0.31	2.47	0.01
SCZ	7	0.34	0.31	1.09	0.28
SCZ	8	-0.01	0.61	-0.02	0.98
SCZ	9	0.06	0.40	0.16	0.87

Supplementary Table S10: Mean polygenic score for each neuropsychiatric condition, by cluster

Table S10a: Mean (SD) PGS for each condition by cluster, training set

Polygenic Score	Overall, N	0, N = 56	1, N = 1,204	2, N = 492	3, N = 118	4, N = 1,214	5, N = 416	6, N = 252	7, N = 246	8, N = 98	9, N = 180	p-value	q-value
**ADHD**	0.08 (1.01)	0.01 (1.01)	0.20 (1.01)	-0.02 (0.98)	0.10 (1.02)	0.08 (1.00)	-0.12 (1.00)	-0.03 (1.02)	0.16 (1.07)	-0.14 (1.00)	0.02 (0.98)	<0.0001	<0.0001
**AN**	0.01 (1.00)	0.05 (0.87)	0.01 (1.01)	-0.11 (1.02)	-0.03 (1.02)	-0.01 (1.00)	-0.11 (1.02)	-0.03 (0.93)	0.06 (0.94)	0.04 (1.04)	0.04 (1.06)	0.11	0.20
**ANX**	0.12 (0.98)	-0.05 (0.91)	0.17 (0.98)	0.01 (1.00)	0.10 (0.91)	0.14 (0.96)	0.08 (1.00)	0.07 (0.98)	0.11 (0.99)	0.17 (1.10)	0.05 (0.98)	0.13	0.20
**BIP**	0.04 (1.00)	0.14 (0.96)	0.11 (1.03)	-0.07 (1.03)	-0.04 (1.10)	0.05 (1.00)	0.01 (0.97)	0.00 (0.93)	-0.03 (0.96)	-0.01 (1.01)	0.08 (1.01)	0.04	0.07
**MDD**	0.18 (1.01)	-0.07 (0.91)	0.27 (1.01)	-0.11 (1.07)	-0.05 (1.01)	0.22 (0.99)	0.03 (1.06)	0.08 (0.88)	0.18 (1.01)	0.11 (0.97)	0.12 (0.96)	<0.0001	<0.0001
**OCD**	0.03 (1.00)	0.03 (0.86)	0.01 (0.99)	-0.01 (0.95)	0.05 (1.01)	0.05 (1.01)	0.01 (0.98)	0.14 (0.99)	-0.06 (1.03)	0.07 (0.91)	0.07 (1.06)	0.70	0.70
**PTSD**	0.07 (0.98)	-0.03 (1.04)	0.12 (0.97)	0.06 (0.96)	0.07 (1.12)	0.09 (0.98)	0.00 (1.01)	0.05 (0.93)	0.02 (0.93)	-0.11 (0.94)	0.02 (0.99)	0.30	0.40
**SCZ**	0.08 (1.02)	0.09 (0.90)	0.15 (1.03)	-0.04 (1.03)	-0.01 (0.96)	0.12 (1.03)	0.00 (1.03)	0.04 (1.06)	0.09 (0.90)	0.10 (1.03)	0.04 (1.03)	0.03	0.07

Table S10b: Mean (SD) PGS for each condition by cluster, projected independent test set

Polygenic Score	Overall, N	0, N = 147	1, N = 624	2, N = 258	3, NULL	4, N = 562	5, N = 214	6, N = 120	7, N = 153	8, NULL	9, N = 82	p-value	q-value
**ADHD**	0.12 (1.02)	0.01 (0.89)	0.27 (1.02)	-0.01 (1.02)		0.20 (1.02)	-0.06 (1.02)	-0.19 (0.97)	0.00 (1.06)		0.08 (0.96)	<0.0001	<0.0001
**AN**	-0.02 (1.02)	0.03 (1.02)	0.00 (1.04)	0.00 (1.01)		-0.07 (1.05)	-0.01 (0.98)	-0.06 (1.09)	0.03 (0.95)		-0.03 (0.94)	0.90	0.90
**ANX**	0.14 (1.01)	0.12 (0.97)	0.19 (1.02)	0.04 (1.03)		0.19 (1.02)	0.05 (1.01)	0.02 (1.02)	0.15 (0.95)		0.04 (1.07)	0.30	0.40
**BIP**	0.04 (1.02)	-0.05 (1.02)	0.11 (1.02)	-0.02 (1.06)		0.07 (1.02)	-0.05 (0.92)	-0.17 (1.06)	0.11 (0.97)		-0.02 (1.04)	0.20	0.20
**MDD**	0.19 (0.99)	0.13 (0.93)	0.25 (0.98)	0.08 (1.03)		0.32 (0.99)	-0.03 (0.96)	0.20 (0.92)	0.21 (1.05)		-0.18 (0.89)	<0.0001	<0.0001
**OCD**	0.01 (1.03)	0.09 (1.05)	0.01 (1.01)	-0.06 (1.05)		0.06 (1.02)	0.00 (1.01)	-0.06 (1.05)	0.00 (0.96)		-0.01 (1.16)	0.70	0.80
**PTSD**	0.08 (0.97)	0.11 (0.90)	0.18 (1.01)	-0.03 (0.98)		0.05 (0.94)	0.03 (0.96)	0.03 (1.08)	0.11 (0.95)		-0.07 (0.93)	0.11	0.20
**SCZ**	0.06 (0.98)	-0.07 (0.99)	0.08 (0.99)	-0.03 (0.93)		0.18 (0.96)	0.07 (1.04)	-0.11 (1.08)	-0.02 (0.99)		0.13 (0.89)	0.029	0.076

Supplementary Table S11: Associations in logistic model between PGS for each condition and cluster (binary, 1=in the specific cluster, 0=in any other cluster)

Table S11a: PGS for each condition by cluster (binary), training set							
**PGS**	**Cluster**	**OR**	**Lower CI**	**Upper CI**	**Std Error**	**z**	**p-value**
ADHD	Cluster 1	1.20	1.12	1.28	0.03	5.28	<0.0001
ADHD	Cluster 2	0.90	0.82	0.99	0.05	-2.13	0.034
ADHD	Cluster 3	1.02	0.85	1.23	0.09	0.26	0.80
ADHD	Cluster 4	1.01	0.94	1.07	0.04	0.18	0.86
ADHD	Cluster 5	0.81	0.73	0.90	0.05	-3.99	<0.0001
ADHD	Cluster 6	0.90	0.79	1.02	0.06	-1.61	0.11
ADHD	Cluster 7	1.09	0.96	1.24	0.07	1.36	0.17
ADHD	Cluster 8	0.80	0.66	0.98	0.10	-2.15	0.031
ADHD	Cluster 9	0.94	0.81	1.09	0.08	-0.78	0.43
ANX	Cluster 1	1.09	1.01	1.16	0.04	2.34	0.019
ANX	Cluster 2	0.88	0.80	0.97	0.05	-2.48	0.013
ANX	Cluster 3	0.97	0.80	1.18	0.10	-0.27	0.78
ANX	Cluster 4	1.04	0.97	1.11	0.03	0.99	0.32
ANX	Cluster 5	0.97	0.87	1.08	0.05	-0.58	0.56
ANX	Cluster 6	0.95	0.83	1.08	0.07	-0.84	0.40
ANX	Cluster 7	0.99	0.87	1.14	0.07	-0.08	0.94
ANX	Cluster 8	1.05	0.85	1.29	0.11	0.42	0.67
ANX	Cluster 9	0.93	0.80	1.09	0.08	-0.88	0.38
BIP	Cluster 1	1.11	1.03	1.19	0.03	2.95	0.0032
BIP	Cluster 2	0.89	0.81	0.98	0.05	-2.40	0.016
BIP	Cluster 3	0.90	0.75	1.09	0.09	-1.08	0.28
BIP	Cluster 4	1.01	0.94	1.08	0.03	0.16	0.87
BIP	Cluster 5	0.97	0.88	1.08	0.05	-0.55	0.58
BIP	Cluster 6	0.97	0.85	1.10	0.07	-0.50	0.62
BIP	Cluster 7	0.93	0.81	1.06	0.07	-1.15	0.25
BIP	Cluster 8	0.95	0.77	1.16	0.10	-0.52	0.60
BIP	Cluster 9	1.05	0.90	1.22	0.08	0.58	0.56
AN	Cluster 1	1.00	0.94	1.07	0.03	0.04	0.97
AN	Cluster 2	0.94	0.86	1.04	0.05	-1.20	0.23
AN	Cluster 3	0.87	0.72	1.05	0.10	-1.42	0.15
AN	Cluster 4	0.97	0.91	1.04	0.03	-0.84	0.40
AN	Cluster 5	1.12	1.01	1.24	0.05	2.19	0.028
AN	Cluster 6	0.95	0.84	1.08	0.07	-0.76	0.44
AN	Cluster 7	1.06	0.93	1.21	0.07	0.86	0.39
AN	Cluster 8	1.20	0.98	1.47	0.10	1.72	0.086
AN	Cluster 9	1.01	0.87	1.18	0.08	0.15	0.88
MDD	Cluster 1	1.15	1.07	1.23	0.03	4.02	<0.0001
MDD	Cluster 2	0.85	0.77	0.93	0.05	-3.48	0.00050
MDD	Cluster 3	1.10	0.91	1.32	0.09	0.97	0.33
MDD	Cluster 4	1.07	1.00	1.14	0.03	1.90	0.057
MDD	Cluster 5	0.85	0.77	0.94	0.05	-3.10	0.0020
MDD	Cluster 6	0.91	0.80	1.03	0.06	-1.50	0.13
MDD	Cluster 7	1.00	0.88	1.14	0.07	0.07	0.95
MDD	Cluster 8	0.93	0.76	1.14	0.10	-0.67	0.51
MDD	Cluster 9	0.96	0.82	1.11	0.08	-0.59	0.55
OCD	Cluster 1	0.98	0.92	1.05	0.03	-0.60	0.55
OCD	Cluster 2	0.98	0.89	1.08	0.05	-0.42	0.67
OCD	Cluster 3	0.95	0.79	1.15	0.09	-0.51	0.61
OCD	Cluster 4	1.03	0.96	1.10	0.03	0.79	0.43
OCD	Cluster 5	0.98	0.89	1.09	0.05	-0.39	0.70
OCD	Cluster 6	1.12	0.98	1.27	0.07	1.69	0.090
OCD	Cluster 7	0.91	0.80	1.04	0.07	-1.38	0.17
OCD	Cluster 8	1.05	0.86	1.28	0.10	0.45	0.65
OCD	Cluster 9	1.03	0.89	1.20	0.08	0.38	0.70
PTSD	Cluster 1	1.09	1.02	1.17	0.04	2.39	0.017
PTSD	Cluster 2	0.98	0.89	1.08	0.05	-0.37	0.71
PTSD	Cluster 3	0.98	0.81	1.19	0.10	-0.19	0.85
PTSD	Cluster 4	1.03	0.96	1.10	0.03	0.83	0.41
PTSD	Cluster 5	0.92	0.83	1.02	0.05	-1.64	0.10
PTSD	Cluster 6	0.97	0.85	1.11	0.07	-0.42	0.67
PTSD	Cluster 7	0.94	0.82	1.07	0.07	-0.94	0.35
PTSD	Cluster 8	0.83	0.67	1.02	0.11	-1.77	0.077
PTSD	Cluster 9	0.94	0.81	1.10	0.08	-0.73	0.47
SCZ	Cluster 1	1.10	1.03	1.17	0.03	2.79	0.0052
SCZ	Cluster 2	0.88	0.80	0.96	0.05	-2.76	0.0058
SCZ	Cluster 3	0.89	0.74	1.06	0.09	-1.29	0.20
SCZ	Cluster 4	1.04	0.98	1.11	0.03	1.21	0.23
SCZ	Cluster 5	0.92	0.83	1.01	0.05	-1.68	0.092
SCZ	Cluster 6	0.97	0.86	1.10	0.07	-0.40	0.69
SCZ	Cluster 7	1.00	0.88	1.13	0.06	-0.01	0.99
SCZ	Cluster 8	1.01	0.82	1.23	0.10	0.05	0.96
SCZ	Cluster 9	0.96	0.83	1.12	0.08	-0.48	0.63

Supplementary Table S11: Associations in logistic model between PGS for each condition and cluster (binary, 1=in the specific cluster, 0=in any other cluster)

Table S11b: PGS for each condition by cluster (binary), projected independent test set

PGS	Cluster	OR	Lower CI	Upper CI	Std Error	z	p-value
ADHD	Cluster 1	1.23	1.12	1.35	0.05	4.23	<0.0001
ADHD	Cluster 2	0.89	0.78	1.02	0.07	-1.73	0.084
ADHD	Cluster 3	NULL					
ADHD	Cluster 4	1.13	1.02	1.24	0.05	2.42	0.015
ADHD	Cluster 5	0.84	0.72	0.96	0.07	-2.49	0.013
ADHD	Cluster 6	0.72	0.59	0.86	0.09	-3.50	0.00047
ADHD	Cluster 7	0.89	0.76	1.05	0.08	-1.39	0.16
ADHD	Cluster 8	NULL					
ADHD	Cluster 9	0.95	0.76	1.19	0.11	-0.41	0.68
ANX	Cluster 1	1.08	0.98	1.19	0.05	1.64	0.10
ANX	Cluster 2	0.91	0.80	1.03	0.07	-1.49	0.14
ANX	Cluster 3	NULL					
ANX	Cluster 4	1.07	0.97	1.18	0.05	1.43	0.15
ANX	Cluster 5	0.91	0.79	1.04	0.07	-1.37	0.17
ANX	Cluster 6	0.88	0.73	1.05	0.09	-1.40	0.16
ANX	Cluster 7	1.03	0.87	1.21	0.08	0.32	0.75
ANX	Cluster 8	NULL					
ANX	Cluster 9	0.92	0.74	1.14	0.11	-0.76	0.44
BIP	Cluster 1	1.13	1.03	1.24	0.05	2.49	0.013
BIP	Cluster 2	0.92	0.81	1.05	0.07	-1.18	0.24
BIP	Cluster 3	NULL					
BIP	Cluster 4	1.04	0.94	1.15	0.05	0.79	0.43
BIP	Cluster 5	0.91	0.79	1.05	0.07	-1.33	0.18
BIP	Cluster 6	0.81	0.67	0.97	0.09	-2.28	0.023
BIP	Cluster 7	1.08	0.91	1.27	0.08	0.86	0.39
BIP	Cluster 8	NULL					
BIP	Cluster 9	0.94	0.75	1.17	0.11	-0.55	0.58
AN	Cluster 1	1.01	0.92	1.11	0.05	0.25	0.81
AN	Cluster 2	1.04	0.91	1.18	0.07	0.59	0.56
AN	Cluster 3						
AN	Cluster 4	0.94	0.85	1.03	0.05	-1.31	0.19
AN	Cluster 5	1.01	0.87	1.16	0.07	0.08	0.94
AN	Cluster 6	0.98	0.81	1.18	0.09	-0.21	0.83
AN	Cluster 7	1.05	0.89	1.24	0.08	0.59	0.56
AN	Cluster 8	NULL					
AN	Cluster 9	1.00	0.80	1.25	0.11	-0.02	0.98
MDD	Cluster 1	1.09	0.99	1.20	0.05	1.66	0.097
MDD	Cluster 2	0.89	0.78	1.02	0.07	-1.69	0.091
MDD	Cluster 3						
MDD	Cluster 4	1.21	1.10	1.34	0.05	3.76	0.00017
MDD	Cluster 5	0.77	0.67	0.89	0.07	-3.48	0.00051
MDD	Cluster 6	1.00	0.83	1.21	0.10	0.02	0.98
MDD	Cluster 7	1.00	0.85	1.19	0.09	0.04	0.97
MDD	Cluster 8	NULL					
MDD	Cluster 9	0.68	0.54	0.85	0.12	-3.34	0.00085
OCD	Cluster 1	0.98	0.90	1.08	0.05	-0.34	0.74
OCD	Cluster 2	0.93	0.82	1.06	0.07	-1.03	0.30
OCD	Cluster 3						
OCD	Cluster 4	1.06	0.97	1.17	0.05	1.28	0.20
OCD	Cluster 5	0.99	0.86	1.14	0.07	-0.14	0.89
OCD	Cluster 6	0.93	0.77	1.11	0.09	-0.83	0.41
OCD	Cluster 7	0.99	0.84	1.17	0.08	-0.08	0.93
OCD	Cluster 8	NULL					
OCD	Cluster 9	0.98	0.79	1.22	0.11	-0.16	0.87
PTSD	Cluster 1	1.17	1.06	1.29	0.05	3.06	0.0022
PTSD	Cluster 2	0.88	0.76	1.01	0.07	-1.86	0.063
PTSD	Cluster 3						
PTSD	Cluster 4	0.96	0.87	1.06	0.05	-0.79	0.43
PTSD	Cluster 5	0.97	0.84	1.12	0.08	-0.42	0.68
PTSD	Cluster 6	0.93	0.77	1.13	0.10	-0.69	0.49
PTSD	Cluster 7	1.03	0.86	1.22	0.09	0.30	0.76
PTSD	Cluster 8	NULL					
PTSD	Cluster 9	0.86	0.68	1.09	0.12	-1.23	0.22
SCZ	Cluster 1	1.03	0.94	1.14	0.05	0.61	0.54
SCZ	Cluster 2	0.88	0.77	1.01	0.07	-1.88	0.060
SCZ	Cluster 3						
SCZ	Cluster 4	1.18	1.07	1.30	0.05	3.23	0.0012
SCZ	Cluster 5	1.01	0.87	1.16	0.07	0.07	0.94
SCZ	Cluster 6	0.83	0.68	1.00	0.10	-1.98	0.048
SCZ	Cluster 7	0.90	0.76	1.06	0.09	-1.28	0.20
SCZ	Cluster 8	NULL					
SCZ	Cluster 9	1.08	0.86	1.36	0.12	0.67	0.50

Supplementary Table S12: Multinomial model with cluster as variable with 9 levels; cluster 9 (low risk) as reference category

Table S12a: Cluster 9 (low risk) as reference category

PRS	Cluster	OR	Lower CI	Upper CI	Std Error	p-value
ADHD	1	1.22	1.04	1.43	0.08	0.01
ADHD	2	0.98	0.82	1.16	0.09	0.79
ADHD	3	1.09	0.89	1.38	0.12	0.45
ADHD	4	1.08	0.92	1.26	0.08	0.37
ADHD	5	0.88	0.74	1.05	0.09	0.17
ADHD	6	0.97	0.80	1.18	0.10	0.76
ADHD	7	1.16	0.96	1.41	0.10	0.13
ADHD	8	0.86	0.67	1.10	0.13	0.24
ADHD	9	Ref	Ref	Ref	Ref	Ref
AN	1	0.98	0.84	1.15	0.08	0.83
AN	2	0.93	0.78	1.11	0.09	0.43
AN	3	0.85	0.67	1.08	0.12	0.19
AN	4	0.96	0.82	1.13	0.08	0.64
AN	5	1.09	0.91	1.30	0.09	0.34
AN	6	0.94	0.77	1.14	0.10	0.51
AN	7	1.03	0.85	1.26	0.10	0.73
AN	8	1.17	0.91	1.50	0.13	0.21
AN	9	Ref	Ref	Ref	Ref	Ref
ANX	1	1.15	0.98	1.35	0.08	0.09
ANX	2	0.97	0.81	1.16	0.09	0.74
ANX	3	1.06	0.83	1.35	0.12	0.63
ANX	4	1.11	0.94	1.30	0.08	0.21
ANX	5	1.05	0.88	1.26	0.09	0.58
ANX	6	1.03	0.84	1.25	0.10	0.79
ANX	7	1.08	0.89	1.31	0.10	0.46
ANX	8	1.14	0.89	1.46	0.13	0.32
ANX	9	Ref	Ref	Ref	Ref	Ref
BIP	1	1.05	0.89	1.22	0.08	0.58
BIP	2	0.88	0.74	1.04	0.09	0.13
BIP	3	0.89	0.70	1.12	0.12	0.30
BIP	4	0.98	0.83	1.14	0.08	0.76
BIP	5	0.95	0.79	1.13	0.09	0.55
BIP	6	0.94	0.78	1.14	0.10	0.55
BIP	7	0.90	0.75	1.10	0.10	0.31
BIP	8	0.92	0.72	1.19	0.13	0.54
BIP	9	Ref	Ref	Ref	Ref	Ref
MDD	1	1.17	1.00	1.37	0.08	0.054
MDD	2	0.91	0.77	1.08	0.09	0.29
MDD	3	1.16	0.92	1.47	0.12	0.21
MDD	4	1.11	0.95	1.30	0.08	0.20
MDD	5	0.91	0.77	1.09	0.09	0.31
MDD	6	0.96	0.80	1.17	0.10	0.70
MDD	7	1.06	0.88	1.29	0.10	0.54
MDD	8	0.99	0.77	1.27	0.13	0.94
MDD	9	Ref	Ref	Ref	Ref	Ref
OCD	1	0.95	0.82	1.12	0.08	0.56
OCD	2	0.95	0.80	1.13	0.09	0.57
OCD	3	0.93	0.73	1.17	0.12	0.52
OCD	4	0.99	0.84	1.16	0.08	0.88
OCD	5	0.95	0.80	1.14	0.09	0.59
OCD	6	1.08	0.89	1.30	0.10	0.46
OCD	7	0.89	0.73	1.08	0.10	0.24
OCD	8	1.01	0.79	1.30	0.13	0.92
OCD	9	Ref	Ref	Ref	Ref	Ref
PTSD	1	1.13	0.97	1.33	0.08	0.12
PTSD	2	1.05	0.88	1.25	0.09	0.58
PTSD	3	1.05	0.83	1.34	0.12	0.69
PTSD	4	1.09	0.93	1.28	0.08	0.29
PTSD	5	0.99	0.83	1.18	0.09	0.89
PTSD	6	1.04	0.86	1.27	0.10	0.68
PTSD	7	1.00	0.82	1.22	0.10	0.96
PTSD	8	0.89	0.69	1.14	0.13	0.36
PTSD	9	Ref	Ref	Ref	Ref	Ref
SCZ	1	1.12	0.96	1.30	0.08	0.17
SCZ	2	0.93	0.78	1.10	0.09	0.39
SCZ	3	0.93	0.74	1.17	0.12	0.55
SCZ	4	1.07	0.92	1.25	0.08	0.38
SCZ	5	0.96	0.81	1.14	0.09	0.67
SCZ	6	1.02	0.84	1.23	0.10	0.87
SCZ	7	1.04	0.86	1.26	0.10	0.69
SCZ	8	1.05	0.82	1.34	0.13	0.72
SCZ	9	Ref	Ref	Ref	Ref	Ref

Supplementary Table S12: Multinomial model with cluster as variable with 9 levels; cluster 9 (low risk) as reference category

Table S12b: Cluster 1 (chronic major depression + trauma) as reference category

PRS	Cluster	OR	Lower CI	Upper CI	Std Error	p-value
ADHD	1	Ref	Ref	Ref	Ref	Ref
ADHD	2	0.80	0.72	0.89	0.05	0.000040
ADHD	3	0.90	0.74	1.09	0.10	0.27
ADHD	4	0.88	0.81	0.96	0.04	0.0021
ADHD	5	0.73	0.65	0.81	0.06	#########
ADHD	6	0.80	0.69	0.91	0.07	0.0010
ADHD	7	0.95	0.83	1.09	0.07	0.48
ADHD	8	0.71	0.57	0.87	0.11	0.00092
ADHD	9	0.83	0.71	0.97	0.08	0.019
AN	1	Ref	Ref	Ref	Ref	Ref
AN	2	0.95	0.85	1.06	0.05	0.34
AN	3	0.87	0.72	1.06	0.10	0.17
AN	4	0.98	0.90	1.06	0.04	0.61
AN	5	1.11	0.99	1.24	0.06	0.074
AN	6	0.95	0.83	1.09	0.07	0.50
AN	7	1.05	0.92	1.21	0.07	0.46
AN	8	1.19	0.97	1.47	0.11	0.10
AN	9	1.01	0.86	1.19	0.08	0.89
ANX	1	Ref	Ref	Ref	Ref	Ref
ANX	2	0.85	0.76	0.94	0.06	0.0024
ANX	3	0.92	0.75	1.12	0.10	0.40
ANX	4	0.97	0.89	1.05	0.04	0.41
ANX	5	0.92	0.82	1.03	0.06	0.14
ANX	6	0.89	0.78	1.03	0.07	0.12
ANX	7	0.94	0.81	1.08	0.07	0.37
ANX	8	0.99	0.80	1.22	0.11	0.90
ANX	9	0.88	0.75	1.04	0.08	0.13
BIP	1	Ref	Ref	Ref	Ref	Ref
BIP	2	0.84	0.75	0.93	0.05	0.0013
BIP	3	0.85	0.70	1.03	0.10	0.092
BIP	4	0.93	0.86	1.01	0.04	0.10
BIP	5	0.91	0.81	1.02	0.06	0.089
BIP	6	0.90	0.79	1.04	0.07	0.14
BIP	7	0.87	0.75	0.99	0.07	0.042
BIP	8	0.88	0.72	1.09	0.11	0.24
BIP	9	0.97	0.83	1.14	0.08	0.72
MDD	1	Ref	Ref	Ref	Ref	Ref
MDD	2	0.78	0.70	0.87	0.05	0.0000047
MDD	3	1.00	0.82	1.21	0.10	0.96
MDD	4	0.95	0.88	1.03	0.04	0.21
MDD	5	0.78	0.70	0.88	0.06	0.000019
MDD	6	0.82	0.72	0.94	0.07	0.0054
MDD	7	0.91	0.79	1.04	0.07	0.18
MDD	8	0.85	0.69	1.04	0.11	0.12
MDD	9	0.87	0.74	1.01	0.08	0.074
OCD	1	Ref	Ref	Ref	Ref	Ref
OCD	2	1.00	0.90	1.11	0.05	0.96
OCD	3	0.97	0.80	1.18	0.10	0.78
OCD	4	1.04	0.96	1.12	0.04	0.39
OCD	5	0.99	0.89	1.12	0.06	0.98
OCD	6	1.13	0.98	1.29	0.07	0.088
OCD	7	0.93	0.81	1.07	0.07	0.33
OCD	8	1.06	0.86	1.31	0.11	0.57
OCD	9	1.04	0.89	1.22	0.08	0.60
PTSD	1	Ref	Ref	Ref	Ref	Ref
PTSD	2	0.93	0.83	1.03	0.06	0.16
PTSD	3	0.93	0.76	1.13	0.10	0.45
PTSD	4	0.96	0.88	1.04	0.04	0.34
PTSD	5	0.87	0.78	0.98	0.06	0.018
PTSD	6	0.92	0.80	1.05	0.07	0.22
PTSD	7	0.89	0.77	1.02	0.07	0.091
PTSD	8	0.78	0.63	0.97	0.11	0.024
PTSD	9	0.89	0.76	1.05	0.08	0.15
SCZ	1	Ref	Ref	Ref	Ref	Ref
SCZ	2	0.83	0.75	0.92	0.05	0.00055
SCZ	3	0.84	0.69	1.01	0.10	0.063
SCZ	4	0.96	0.89	1.04	0.04	0.35
SCZ	5	0.86	0.77	0.97	0.06	0.010
SCZ	6	0.91	0.80	1.04	0.07	0.18
SCZ	7	0.93	0.82	1.07	0.07	0.32
SCZ	8	0.94	0.77	1.16	0.11	0.58
SCZ	9	0.90	0.77	1.05	0.08	0.20

SUPPLEMENTARY METHODS

Norwegian Mother, Father, and Child Cohort Study

The Norwegian Mother, Father, and Child Cohort Study (MoBa) is a population-based study comprising approximately 114,500 children, 95,200 mothers, and 75,200 fathers, linked to data from the Medical Birth Registry of Norway (MBRN), a national registry of all births. From 1999-2008, participants were recruited at their first ultrasound (gestational week 17-19) across Norway.[1,2] All pregnant women in Norway were eligible and 41% consented to participation. Participants completed questionnaires at three pregnancy timepoints and 6-months postpartum, with follow-up ongoing. Blood samples were collected from both parents during pregnancy, and maternal and umbilical cord blood were collected at childbirth.[3] Full details of MoBa genotyping, quality-control, and imputation have previously been described.[4]

Study Sample

The study sample includes women from the MoBa cohort with PPD who completed the Edinburgh Postnatal Depression Scale (EPDS) on the 6-month postpartum questionnaire. PPD was defined as a score of ≥8 on the 6-item short version of the EPDS, equivalent to ≥11 on the full EPDS,[5,6] a definition widely used in prior MoBa papers,[7,8] and the field.[9-12] If a participant had PPD in multiple pregnancies, only the first recorded pregnancy was included. Participants were excluded if they did not complete the baseline questionnaire at recruitment; had a multiple (e.g., twins), preterm (<34 weeks gestation), or stillbirth; or their infant died prior to the 6-month assessment. The final study sample included 7,859 women with PPD.

Our sample size of 7,859 women with PPD was determined by the available data in the MoBa cohort meeting our inclusion criteria. This sample size is substantially larger than many previous studies of PPD heterogeneity, which have typically included fewer than 1,000 participants unless they also included individuals without depressive symptoms in their samples.[13] For the unsupervised clustering analysis, our sample provided sufficient power to detect clusters that represent at least 2% of the full cohort (approximately 160 individuals), which is adequate for identifying clinically meaningful subtypes.

Clustering Input Variables

We selected features as input variables in the unsupervised clustering algorithm based on their ability to discriminate PPD etiologies and clinical trajectories in prior studies, as well as their ability to be evaluated in pre- and postnatal clinical care settings. These included measures of PPD symptom severity[14-17] [*EPDS score, self-reported medication use for depression*]; comorbid anxiety and anger during pregnancy and the postpartum period[14,18] [*self-reported medication use for anxiety, Hopkins Symptoms Checklist anxiety subscale score, Differential Emotions Scale anger subscale score*]; psychiatric history[19-22] [*self-reported history or symptoms of depression, anxiety, or eating disorder before pregnancy*]; timing of symptom onset[22-24] [*depressive symptoms reported in early pregnancy, late pregnancy, or early postpartum*]; substance use during pregnancy[13,25] [*self-reported alcohol consumption or smoking*]; trauma history[19-21] [*number of adverse life events experienced in the year prior to pregnancy, lifetime history of childhood and adulthood physical and sexual abuse*]; and physical impairment in late pregnancy and the early postpartum[13,15,18,20,25] [*severe pelvic pain during the third trimester, maternal postpartum length of hospital stay, self-rated physical health at 6 months postpartum*]. Further details are provided in Table S1.

Auxiliary Characterization Variables

After clustering, we evaluated relationships between clusters and auxiliary variables relevant to perinatal mental health, including demographic factors, social support, medical comorbidities, pregnancy complications, delivery and birth factors, infant complications, and reproductive factors. Unlike clustering inputs, these auxiliary variables were not psychiatric measures nor known to distinguish PPD subtypes; rather, they were chosen for their relevance to the perinatal period and previous associations with PPD. A full list of auxiliary characterization variables is available in Table S2.

Polygenic Scores (PGS) for neuropsychiatric conditions

We calculated polygenic scores (PGS) for neuropsychiatric conditions using PRSice2,[26] with details in Tables S3 and S4. Scores were weighted sums of risk alleles per individual, based on effect sizes from the latest published GWAS of anorexia nervosa,[27] attention deficit hyperactivity disorder (ADHD),[28] anxiety disorder,[29] bipolar disorder,[30] MDD,[31] obsessive compulsive disorder (OCD),[32] post-traumatic stress disorder (PTSD),[33] and schizophrenia[34] (Table S5). Due to sample overlap with the PPD GWAS,[8] a PPD PGS could not be effectively calculated. For each of the eight conditions, ten separate PGS were generated using sets of SNPs at increasing p-value thresholds of association: $p < 5.0 \times 10^{-8}$ (the field standard for genome-wide significance[35]), 5.0×10^{-7}, 5.0×10^{-6}, 5.0×10^{-5}, 0.001, 0.005, 0.01, 0.1, 0.5, 1 (all SNPS). As a preprocessing step to account for potential confounding due to population stratification and technical batch effects, we residualised the PGS by regressing them on genotype batch and the first 10 genetic principal components. We used linear regression models with normally distributed random errors and identity link function at all thresholds for each of the eight conditions. We verified the distributional and structural assumptions of our regression models through diagnostic plots of residuals (to assess normality) and plots of residuals versus fitted values (to assess linearity and homoscedasticity). Model assumptions were adequately met across all analyses. Examples of our diagnostic plots for one of the traits (ADHD) are shown below in the section entitled *Plots - Diagnostics, Parameter Tuning, and Sensitivity Analysis*. Following Coombes et al.[36], we derived a

single polygenic predictor for each condition by extracting the first principal component across all thresholds, which explained 40-67% of the variance across the different PGS thresholds for each condition, maximizing predictive power while minimizing overfitting, as previously applied to genetic risk studies in MoBa.[37] For the principal component analysis, we used unrotated principal components, as our goal was to maximize the variance explained and create one composite polygenic score that captures the maximum shared genetic signal across thresholds. Thus, we compute eight different composite polygenic scores, one for each trait. Plots of proportion of variance explained by each of the 10 PCs and of PC loadings by p-value threshold for each of the eight conditions are presented in the *Plots - Diagnostics, Parameter Tuning, and Sensitivity Analysis* section.

Statistical Analysis
We performed all analyses in R (v4.2.1). Exploratory data analysis was conducted at the start of data processing and diagnostics were performed at each new data engineering or modeling step within the analytic process. We broadly apply the tools in the R package *DataExplorer (https://cran.r-project.org/web/packages/DataExplorer/)* for assessment of data missingness, distribution, and correlation to test statistical assumptions. Raw survey data from the full MoBa cohort were processed using the *phenotools*[38] package to ensure systematic coding of survey instruments and reproducibility. Implausible values of age of menarche <6 years or >35 years were set to missing, and missing data for all variables were imputed using random forest nonparametric missing value imputation (R package: *missForest*).[39] The imputation model was applied to all first pregnancies in the full MoBa cohort (n=69,755) and included all clustering input variables and auxiliary characterization variables (Tables S1 and S2). As our proportion of missing data was relatively low (<5%), we used the default missForest implementation with 10 trees and a maximum of 10 iterations, parallelized across 8 CPU cores. The algorithm imputes missing values iteratively by fitting a random forest for each variable with missingness, using all other variables as predictors. Continuous and categorical variables were handled simultaneously. Final imputed values were extracted as a complete dataset using mf_imputation$ximp. After data cleaning, eligible PPD cases were then identified, and following standard machine learning practices for model development and validation[40], we randomly split eligible PPD cases into a training set (2/3, n = 5,239) and an independent test set reserved for projection and validation (1/3, n = 2,620).

A 2/3 : 1/3 split was chosen to maintain a balance of statistical power with validation; as our clustering aim is exploratory, it requires larger training samples, but the test set must also be sufficiently large to have enough independent examples to check stability. Both sets were derived from the same overall MoBa cohort but included different individuals, with random assignment to either the training or test set.

While k-fold cross-validation is often more efficient for model performance estimation, we opted for a split-sample validation approach to enable a true independent validation of our clustering solution. This approach allowed us to test whether the identified clusters truly represent stable, reproducible subtypes that would be found in a new sample, rather than potentially overfitting to the specific characteristics of our training data. Additionally, having a separate test set allowed for a more straightforward assessment of the reproducibility of specific cluster-variable associations, which is central to our research aims.

For unsupervised clustering, we used the Uniform Manifold Approximation and Projection (UMAP, R package: *umap*[41]) to reduce dimensionality and project in a two-dimensional layout. Data were scaled from zero to one before UMAP application with default parameters and Euclidean distance matrix. This approach accommodates zero inflation and mixed data types and enables projection of an independent test set onto the same manifold as the training set. We then applied the Density-Based Spatial Clustering of Applications with Noise (DBSCAN, R package: *dbscan*[42]) that identifies clusters of varying shape and size and handles outliers well.[43] Optimal parameters were determined using kNN plots and average silhouette distance for a range of epsilon and minimum point values, resulting in *eps = 2.0* and *MinPts = 75*. To identify features that differentiate clusters, we visualized clusters with radar plots (R package: *fmsb*).[44] and computed summary statistics. Statistical comparisons were conducted using the R package *gtsummary*. For continuous variables, the non-parametric Kruskal-Wallis rank-sum test was used by default, providing robustness to non-normal distributions when comparing across categorical groups. For categorical variables, Pearson's Chi-square tests were used unless expected cell counts fell below five, in which case Fisher's exact tests were automatically applied.

For PGS, we aimed to estimate differences in genetic liability to neuropsychiatric conditions across PPD subtypes. Mean PGS scores for anorexia nervosa, ADHD, anxiety disorder, bipolar disorder, MDD, OCD, PTSD, and schizophrenia were calculated for each PPD cluster as described above. Next, logistic regressions were performed including each PGS as exposures in eight separate models with each specific cluster as outcome (1=in the specific cluster, 0=in any other cluster). Models were adjusted for the first 10 genetic principal components, genotyping batch, and imputation batch. Given that this was a study of perinatal women who were similar for age and sex, these were not included as adjustment variables in the model.

In all statistical analyses, corrections for multiple comparisons were implemented using the False Discovery Rate (FDR) method.[45] This approach is particularly suitable for our study where test statistics are not independent, as it allows for a more powerful and less conservative error rate control compared to traditional methods like Bonferroni. We applied FDR correction to maintain the familywise error rate at a significance level of $p < 0.05$ for each group of related tests (i.e., associations between cluster and 1) input variables, 2) auxiliary variables, and 3) PGS were considered three different groups of related tests for FDR correction).

Independent Test Set Projection and Validation

After clustering, characterizing, and conducting genetic analyses on the training set, we projected the reserved independent test data onto the same manifold (i.e., high-dimensional space) used for the training data. We applied DBSCAN with identical parameters to the test set,[46] noting associations replicated if they met an FDR-corrected q-value <0.05 in both sets. Nearest neighbor linking between sets allowed us to compute the Adjusted Rand Index (AMI)[47] and Fowlkes-Mallows Index (FMI)[48] to empirically evaluate concordance between training and projected independent test set clusters.

Plots - Diagnostics, Parameter Turning, and Sensitivity Analyses

Percentage of missing data by feature

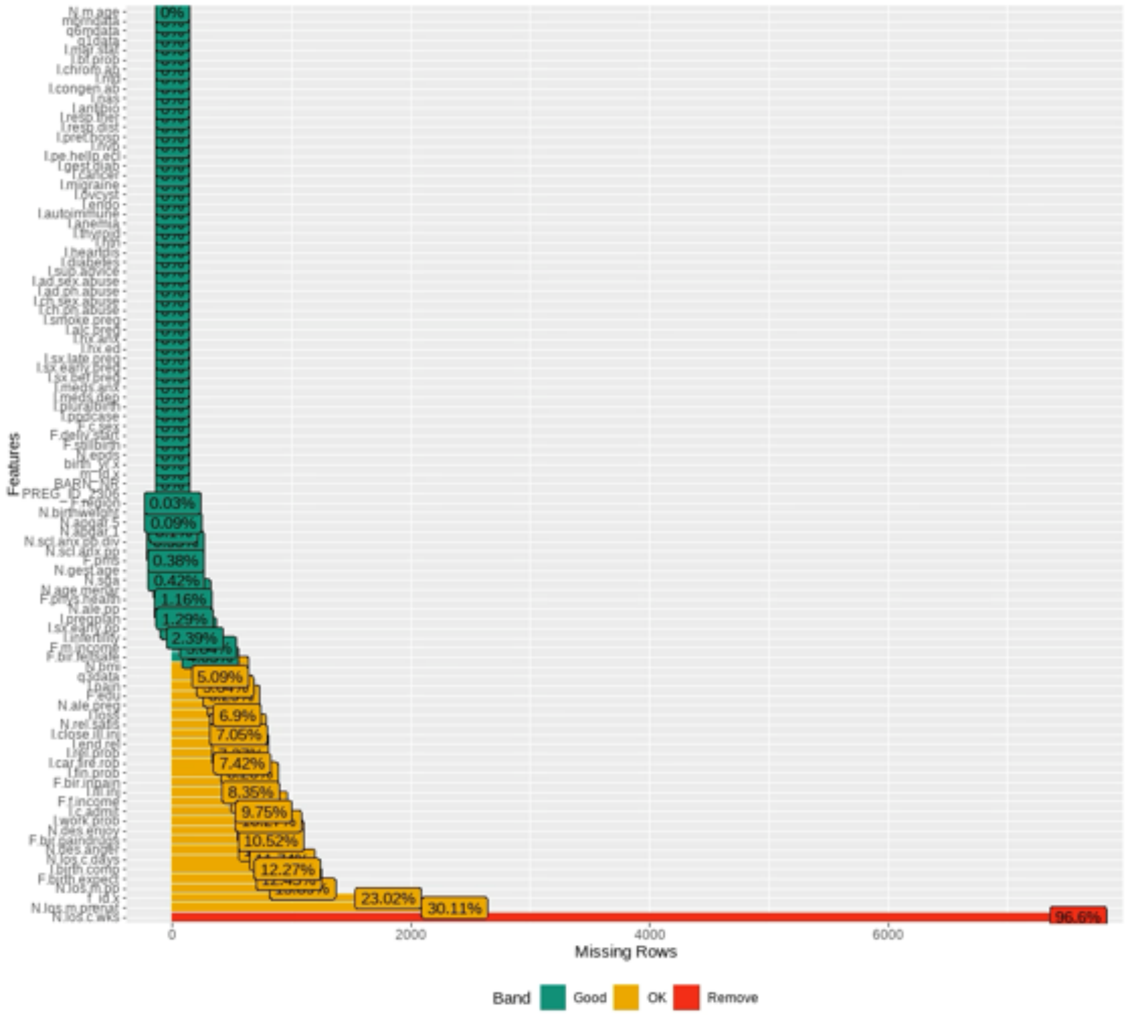

Proportion of variance explained by each principal component in PGS PCA
PC1 loadings for each p-value threshold

ADHD	
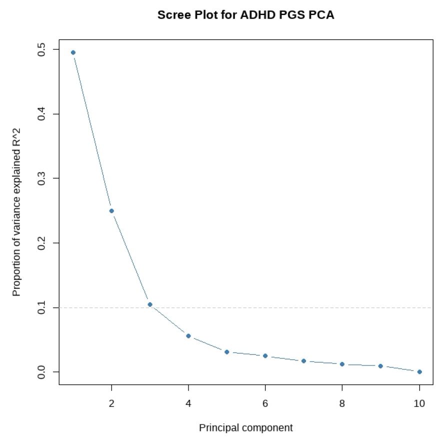	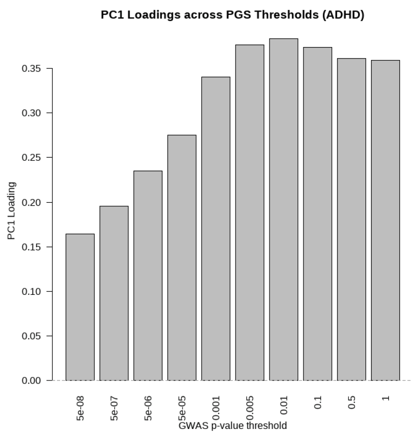
AN	
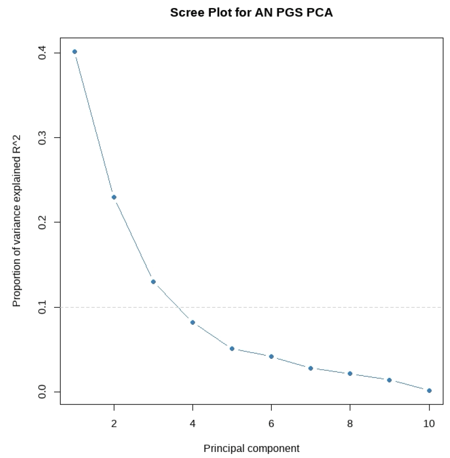	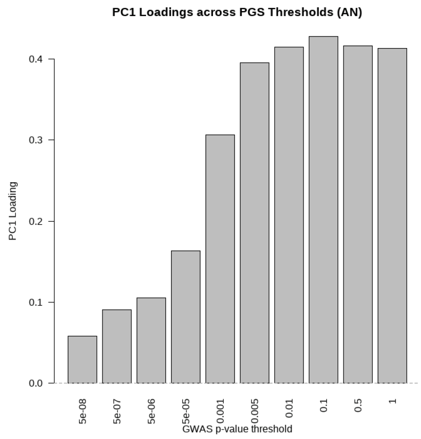
ANX	
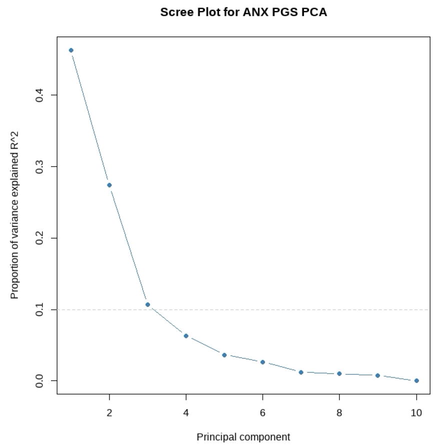	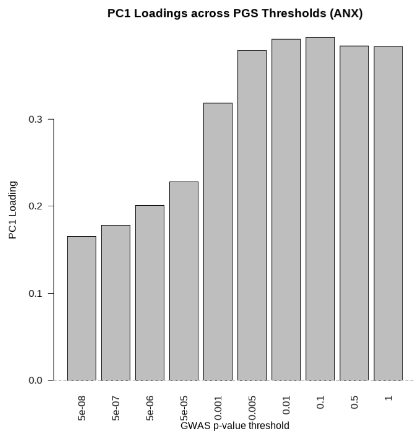
BIP	

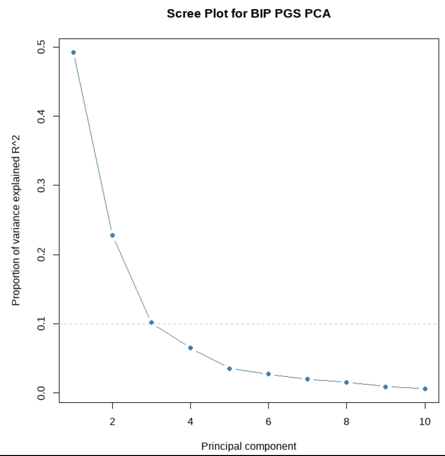
Scree Plot for BIP PGS PCA
Proportion of variance explained R^2
Principal component

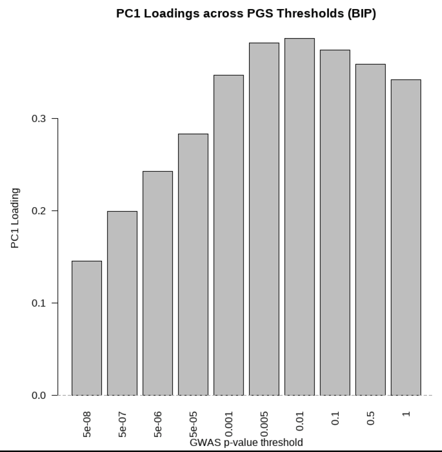
PC1 Loadings across PGS Thresholds (BIP)
PC1 Loading
5e-08
5e-07
5e-06
5e-05
0.001
0.005
0.01
0.1
0.5
1
GWAS p-value threshold

MDD

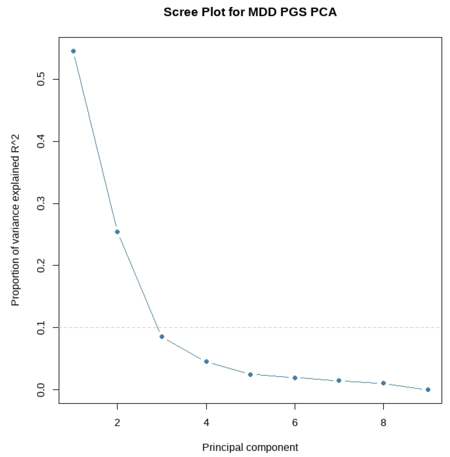
Scree Plot for MDD PGS PCA
Proportion of variance explained R^2
Principal component

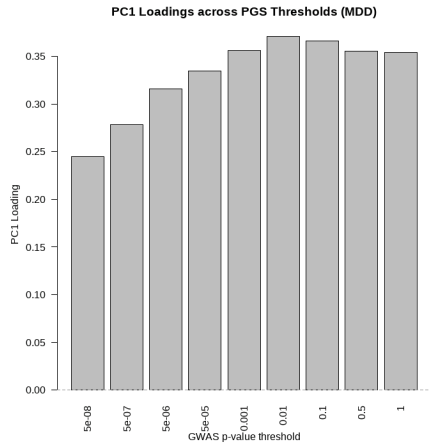
PC1 Loadings across PGS Thresholds (MDD)
PC1 Loading
5e-08
5e-07
5e-06
5e-05
0.001
0.01
0.1
0.5
1
GWAS p-value threshold

OCD

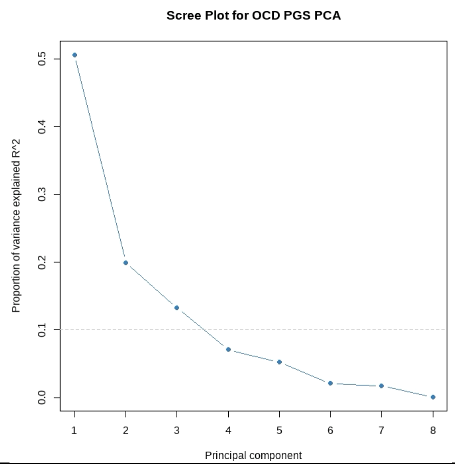
Scree Plot for OCD PGS PCA
Proportion of variance explained R^2
Principal component

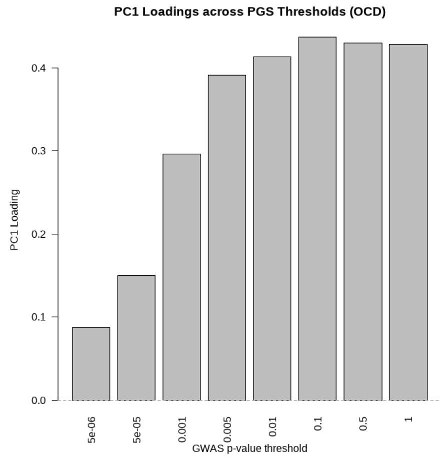
PC1 Loadings across PGS Thresholds (OCD)
PC1 Loading
5e-06
5e-05
0.001
0.005
0.01
0.1
0.5
1
GWAS p-value threshold

PTSD

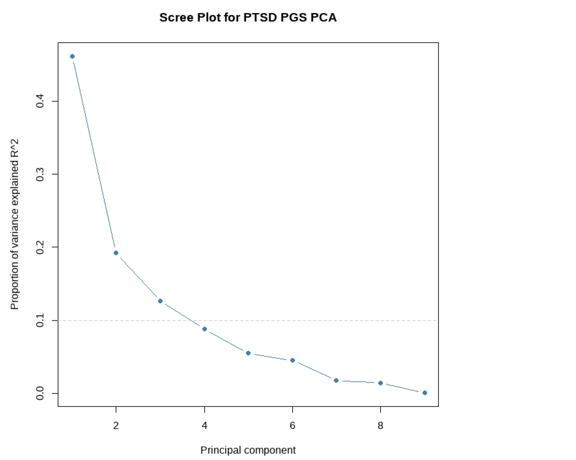
Scree Plot for PTSD PGS PCA
Proportion of variance explained R^2
0.0
0.1
0.2
0.3
0.4
2
4
6
8
Principal component

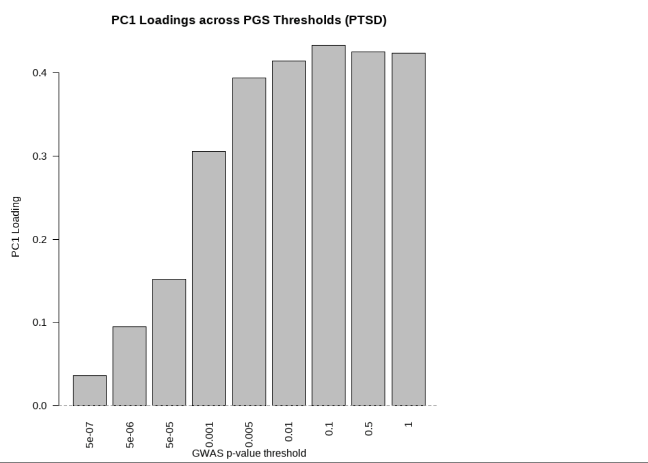
PC1 Loadings across PGS Thresholds (PTSD)
PC1 Loading
0.0
0.1
0.2
0.3
0.4
5e-07
5e-06
5e-05
0.001
0.005
0.01
0.1
0.5
1
GWAS p-value threshold

SCZ

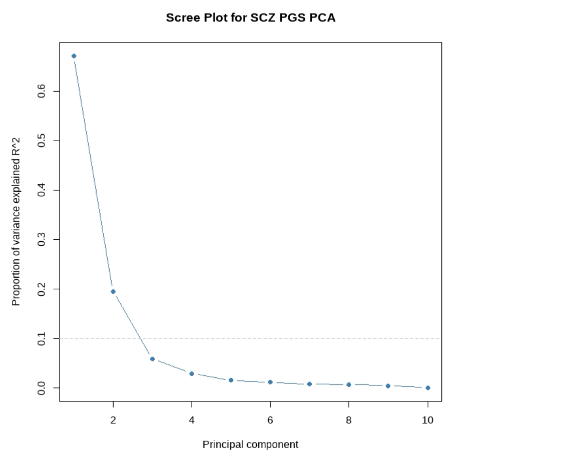
Scree Plot for SCZ PGS PCA
Proportion of variance explained R^2
0.0
0.1
0.2
0.3
0.4
0.5
0.6
2
4
6
8
10
Principal component

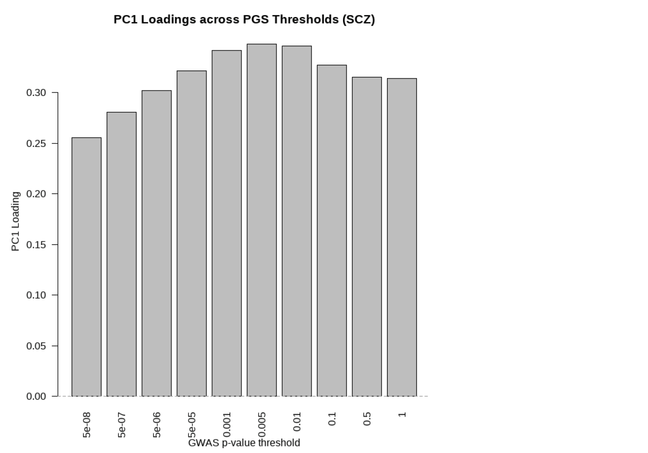
PC1 Loadings across PGS Thresholds (SCZ)
PC1 Loading
0.00
0.05
0.10
0.15
0.20
0.25
0.30
5e-08
5e-07
5e-06
5e-05
0.001
0.005
0.01
0.1
0.5
1
GWAS p-value threshold

Histograms of residuals by threshold for ADHD PGS

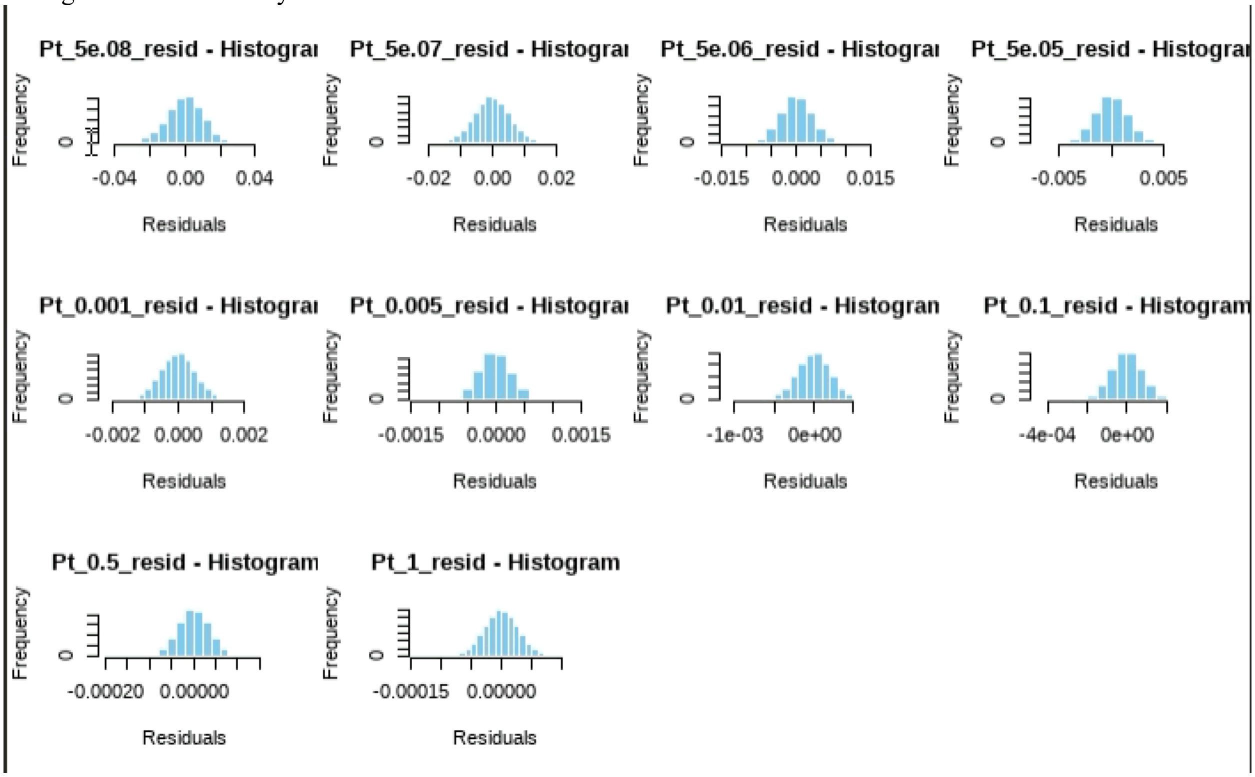

Q-Q plots of residualised ADHD PGS by threshold

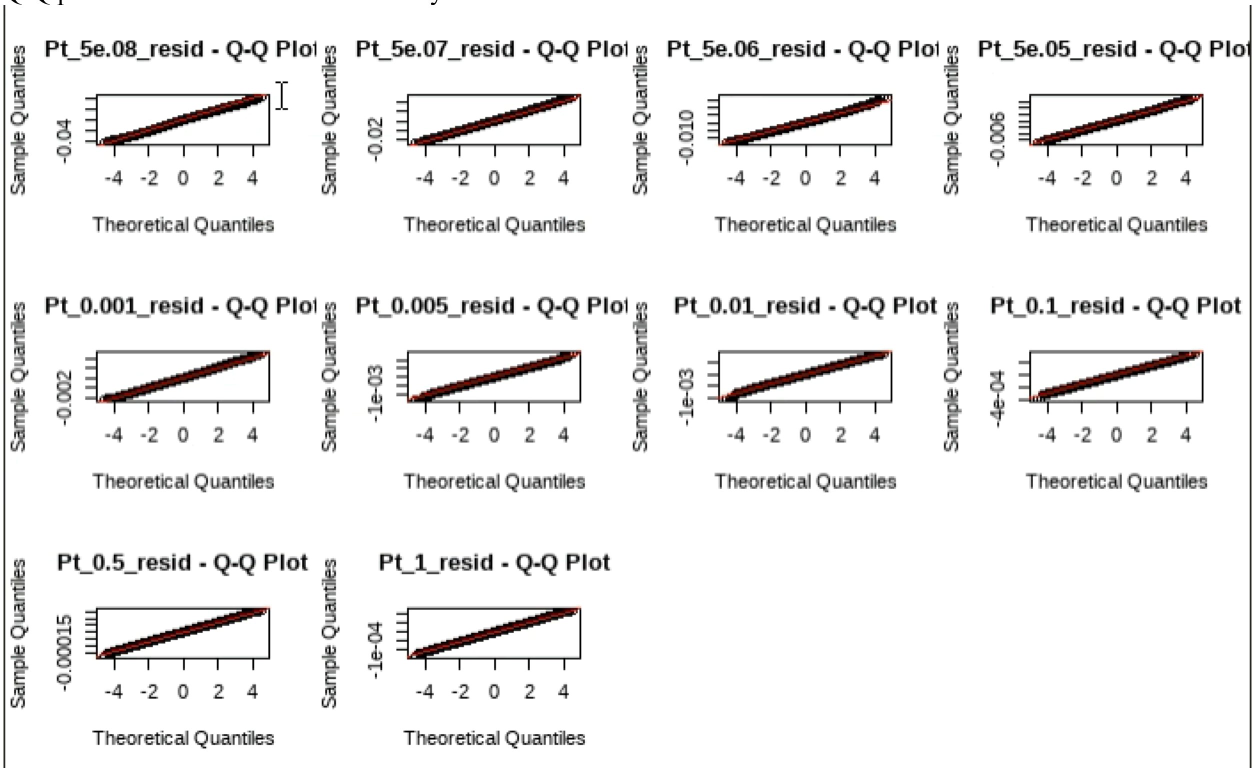

Residuals vs Fitted

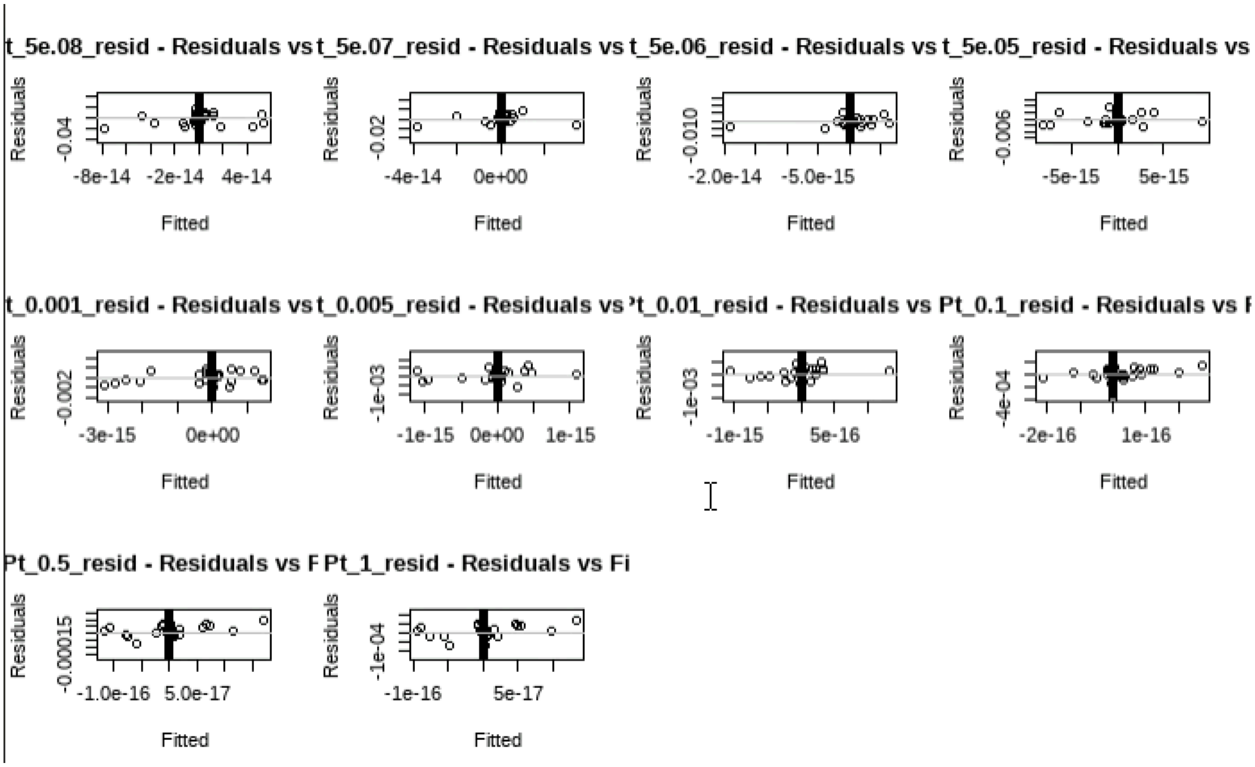

PGS Multinomial Model with cluster as multi-level variable with more than two categories; Reference category as Cluster 9: low risk (accompanies Table S12a)

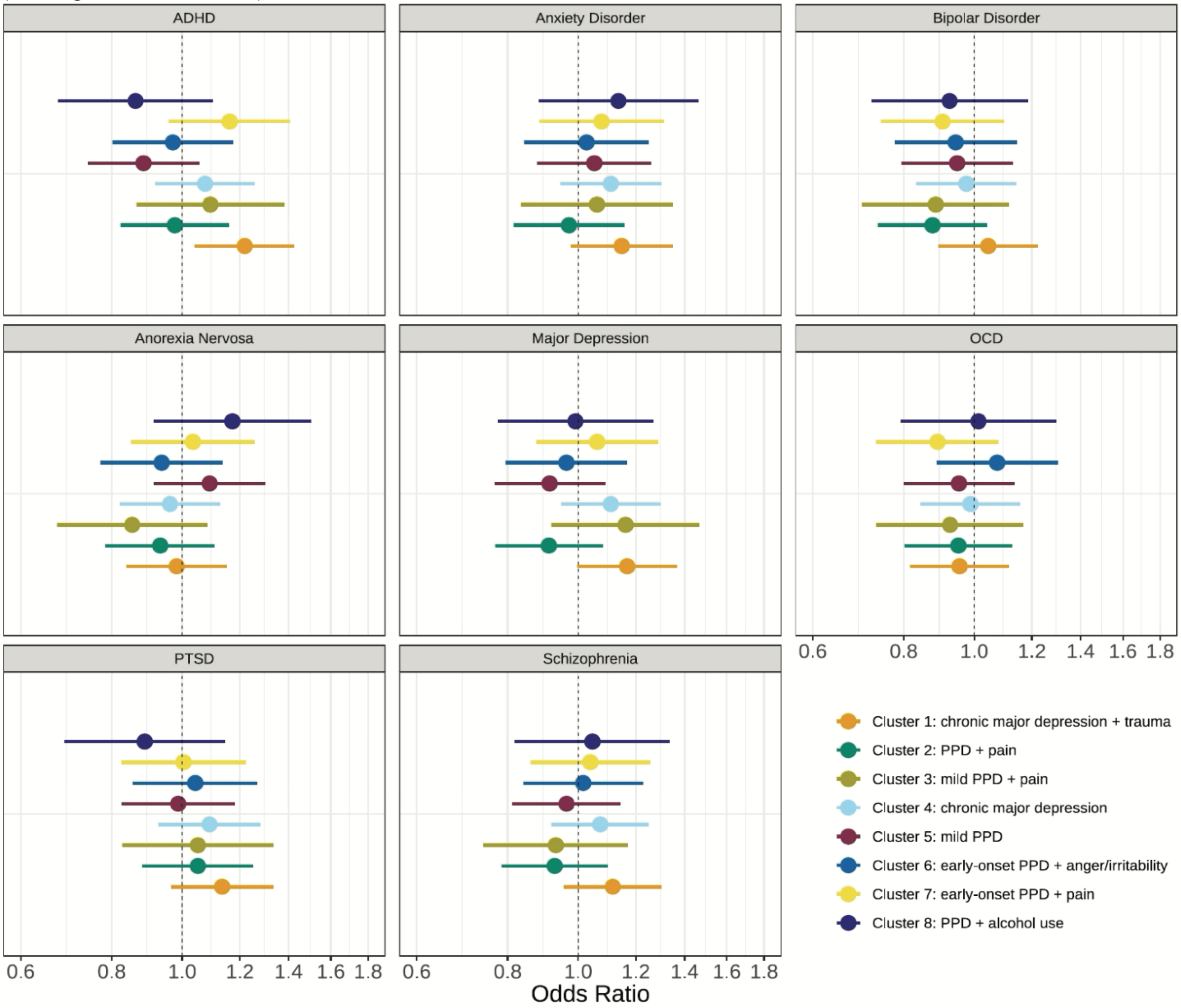

PGS Multinomial Model with cluster as multi-level variable with more than two categories; Reference category as Cluster 1: chronic depression + trauma (accompanies Table S12b)

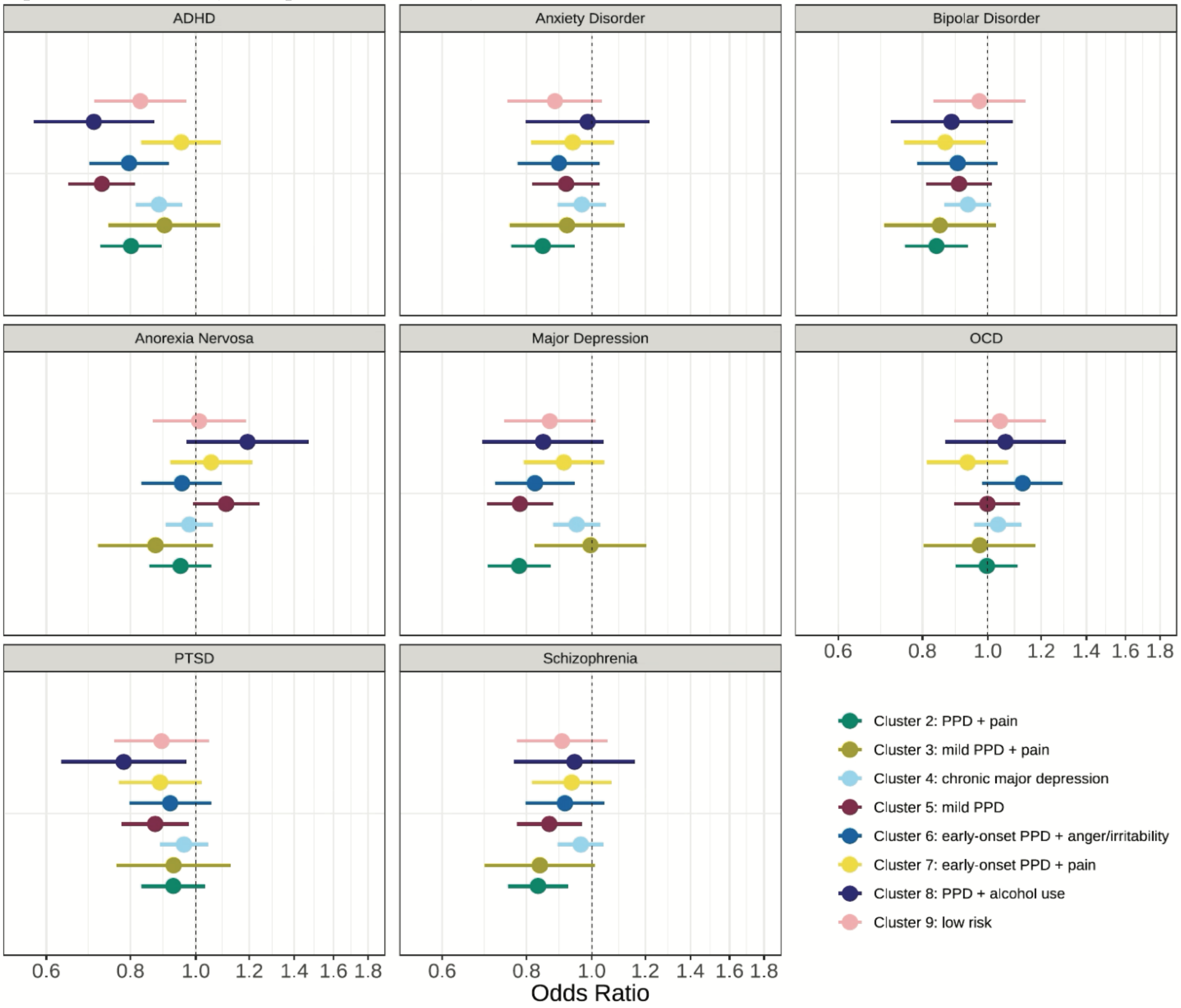

References

1. Magnus P, Birke C, Vejrup K, et al. Cohort Profile Update: The Norwegian Mother and Child Cohort Study (MoBa). *International Journal of Epidemiology* 2016; **45**(2): 382-8.
2. Magnus P, Irgens LM, Haug K, et al. Cohort profile: The Norwegian Mother and Child Cohort Study (MoBa). *International Journal of Epidemiology* 2006; **35**(5): 1146-50.
3. Paltiel L, Haugan A, Skjerden T, et al. The biobank of the Norwegian Mother and Child Cohort Study -- present status. *Nor J Epidemiol* 2014; **24**(1-2): 29-35.
4. Corfield EC, Frei O, Shadrin AA, et al. The Norwegian Mother, Father, and Child cohort study (MoBa) genotyping data resource: MoBaPsychGen pipeline v.1. *bioRxiv* 2022: 2022.06.23.496289-2022.06.23.
5. Cox JL, Holden JM, Sagovsky R. Detection of Postnatal Depression: Development of the 10-item Edinburgh Postnatal Depression Scale. *British Journal of Psychiatry* 1987; **150**: 782-6.
6. Eberhard-Gran M, Eskild A, Tambs K, Opjordsmoen S, Samuelsen SO. Review of validation studies of the Edinburgh postnatal depression scale. *Acta Psychiatrica Scandinavica* 2001; **104**(4): 243-9.
7. Eberhard-Gran M, Eskild A, Tambs K, Schei B, Opjordsmoen S. The Edinburgh postnatal depression scale: Validation in a Norwegian community sample. *Nordic Journal of Psychiatry* 2001; **55**(2): 113-7.
8. Guintivano J, Byrne EM, Kiewa J, et al. Meta-Analyses of Genome-Wide Association Studies for Postpartum Depression. *American Journal of Psychiatry* 2023; **180**(12): 884-95.
9. Ammon Avalos L, Galindo C, Li D-K, et al. Pregnancy complications in polycystic ovary syndrome patients. *American journal of epidemiology* 2014; **319**(1): 1617-.
10. Cox JL, Murray D, Chapman G. A Controlled Study of the Onset, Duration and Prevalence of Postnatal Depression. *The British Journal of Psychiatry* 1993; **163**: 27-31.
11. Boyd RC, Le HN, Somberg R. Review of screening instruments for postpartum depression. *Archives of Women's Mental Health* 2005; **8**(3): 141-53.
12. Hewitt CE, Gilbody SM. Is it clinically and cost effective to screen for postnatal depression: A systematic review of controlled clinical trials and economic evidence. *BJOG: An International Journal of Obstetrics and Gynaecology* 2009; **116**(8): 1019-27.
13. Waqas A, Nadeem M, Rahman A. Exploring Heterogeneity in perinatal depression: a comprehensive review. *BMC Psychiatry* 2023; **23**(1): 643-.
14. Putnam KT, Wilcox M, Robertson-Blackmore E, et al. Clinical phenotypes of perinatal depression and time of symptom onset: analysis of data from an international consortium. *The Lancet Psychiatry* 2017; **4**(6): 477-85.
15. Sun J-W, Cao D-F, Li J-H, et al. Profiles and characteristics of clinical subtypes of perinatal depressive symptoms: A latent class analysis. *Journal of advanced nursing* 2019; **75**(11): 2753-65.
16. Baron E, Bass J, Murray SM, Schneider M, Lund C. A systematic review of growth curve mixture modelling literature investigating trajectories of perinatal depressive symptoms and associated risk factors. *Journal of Affective Disorders* 2017; **223**(February): 194-208.
17. Hammerton G, Mahedy L, Mars B, et al. Association between Maternal Depression Symptoms across the First Eleven Years of Their Child's Life and Subsequent Offspring Suicidal Ideation. *PloS one* 2015; **10**(7): e0131885-e.
18. Saldaña KS, Shaffer JA, Everhart KD, Kim SL, Kaplan PS. Dimensions of depressive symptomatology in mothers derived from factor analyses. *Archives of gynecology and obstetrics* 2022; **306**(3): 707-15.
19. Eastwood J, Wang A, Khanlari S, Montgomery A, Yang JYH. Psychosocial stratification of antenatal indicators to guide population-based programs in perinatal depression. *BMC pregnancy and childbirth* 2021; **21**(1): 277-.
20. Fisher SD, Sit DK, Yang A, Ciolino JD, Gollan JK, Wisner KL. Four maternal characteristics determine the 12-month course of chronic severe postpartum depressive symptoms. *Depression and anxiety* 2019; **36**(4): 375-83.
21. Giallo R, Pilkington P, McDonald E, Gartland D, Woolhouse H, Brown S. Physical, sexual and social health factors associated with the trajectories of maternal depressive symptoms from pregnancy to 4 years postpartum. *Social psychiatry and psychiatric epidemiology* 2017; **52**(7): 815-28.
22. Tebeka S, Le Strat Y, Mandelbrot L, et al. Early- and late-onset postpartum depression exhibit distinct associated factors: the IGEDEPP prospective cohort study. *BJOG: An International Journal of Obstetrics & Gynaecology* 2021; **128**(10): 1683-93.
23. Aoyagi S-S, Takei N, Nishimura T, Nomura Y, Tsuchiya KJ. Association of late-onset postpartum depression of mothers with expressive language development during infancy and early childhood: the HBC study. *PeerJ* 2019; **7**(3): e6566-e.
24. van der Waerden J, Galéra C, Saurel-Cubizolles MJ, Sutter-Dallay AL, Melchior M, Group EMCCS. Predictors of persistent maternal depression trajectories in early childhood: results from the EDEN mother-child cohort study in France. *Psychological medicine* 2015; **45**(9): 1999-2012.
25. Waqas A, Rahman A. Does One Treatment Fit All? Effectiveness of a Multicomponent Cognitive Behavioral Therapy Program in Data-Driven Subtypes of Perinatal Depression. *Frontiers in Psychiatry* 2021; **12**(November): 1-12.
26. Choi SW, Shin T, Mak H, Reilly PFO. A guide to performing Polygenic Risk Score analyses. 2010; **5**: 11-3.
27. Watson HJ, Yilmaz Z, Thornton LM, et al. Genome-wide association study identifies eight risk loci and implicates metabo-psychiatric origins for anorexia nervosa. *Nature genetics* 2019; **51**(8): 1207-14.
28. Demontis D, Walters GB, Athanasiadis G, et al. Genome-wide analyses of ADHD identify 27 risk loci, refine the genetic architecture and implicate several cognitive domains. *medRxiv* 2022; **15**: 2022.02.14.22270780-2022.02.14.

29. Purves KL, Coleman JRI, Meier SM, et al. A major role for common genetic variation in anxiety disorders. *Molecular Psychiatry* 2020; **25**(12): 3292-303.
30. Mullins N, Forstner AJ, O'Connell KS, et al. Genome-wide association study of more than 40,000 bipolar disorder cases provides new insights into the underlying biology. *Nature genetics* 2021; **53**(6): 817-29.
31. Wray NR, Ripke S, Mattheisen M, et al. Genome-wide association analyses identify 44 risk variants and refine the genetic architecture of major depression. *Nature genetics* 2018; **50**(5): 668-81.
32. International Obsessive Compulsive Disorder Foundation Genetics C, Studies OCDCGA. Revealing the complex genetic architecture of obsessive-compulsive disorder using meta-analysis. *Molecular psychiatry* 2018; **23**(5): 1181-8.
33. Logue MW, Amstadter AB, Baker DG, et al. The Psychiatric Genomics Consortium Posttraumatic Stress Disorder Workgroup: Posttraumatic Stress Disorder Enters the Age of Large-Scale Genomic Collaboration. *Neuropsychopharmacology : official publication of the American College of Neuropsychopharmacology* 2015; **40**(10): 2287-97.
34. Trubetskoy V, Pardiñas AF, Qi T, et al. Mapping genomic loci implicates genes and synaptic biology in schizophrenia. *Nature* 2022; **604**(7906): 502-8.
35. Dudbridge F, Gusnanto A. Estimation of significance thresholds for genomewide association scans. *Genetic Epidemiology* 2008; **32**(3): 227-34.
36. Coombes BJ, Ploner A, Bergen SE, Biernacka JM. A principal component approach to improve association testing with polygenic risk scores. *Genetic Epidemiology* 2020; **44**(7): 676-86.
37. Hannigan LJ, Askeland RB, Ask H, et al. Developmental milestones in early childhood and genetic liability to neurodevelopmental disorders. *Psychological Medicine* 2023; **53**(5): 1750-8.
38. Hannigan LJ, Corfield E, Askelund A, et al. phenotools: an R package to facilitate efficient and reproducible use of phenotypic data from MoBa and linked registry sources in the TSD environment. OSF; 2021.
39. Stekhoven DJ, Bühlmann P. MissForest--non-parametric missing value imputation for mixed-type data. *Bioinformatics (Oxford, England)* 2012; **28**(1): 112-8.
40. Hastie T, Tibshirani R, Friedman J. The Elements of Statistical Learning. *Springer Series in Statistics* 2009.
41. McInnes L, Healy J, Melville J. UMAP: Uniform Manifold Approximation and Projection for Dimension Reduction. 2018.
42. Hahsler M, Piekenbrock M, Doran D. dbscan : Fast Density-Based Clustering with R. *Journal of Statistical Software* 2019; **91**(1).
43. Ester M, Kriegel H-P, Sander J, Xu X. A Density-Based Algorithm for Discovering Clusters in Large Spatial Databases with Noise. *KDD Proceedings* 1996; **96**(34): 226-31.
44. Nakazawa M. Package 'fmsb': Functions for Medical Statistics Book with some Demographic Data. 2024.
45. Benjamini Y, Hochberg Y. Controlling the False Discovery Rate: A Practical and Powerful Approach to Multiple Testing. *Journal of the Royal Statistical Society: Series B (Methodological)* 1995; **57**(1): 289-300.
46. Liu T, Yu H, Blair RH. Stability estimation for unsupervised clustering: A review. *Wiley Interdisciplinary Reviews: Computational Statistics* 2022; **14**(6): 1-17.
47. Rand WM. Objective criteria for the evaluation of clustering methods. *Journal of the American Statistical Association* 1971; **66**(336): 846-50.
48. Fowlkes EB, Mallows CL. A Method for Comparing Two Hierarchical Clusterings. *Journal of the American Statistical Association* 1983; **78**(383): 553-69.

Research in Context: Evidence before this study
Full List of Terms Searched in NHGRI-EBI GWAS Catalog

breast cancer
cervical cancer
ovarian cancer
uterine cancer
endometrial cancer
petioneal cancer
prostate cancer
testicular cancer
osteoporosis
premenstrual syndrome
premenstrual dysphoric disorder
migraine
use of hormonal birth control
use of psychotropic medication
fibroadenoma
breast cyst
breastfeeding
Kallman Syndrome
Turner Syndrome
polycystic ovarian syndrome
endometriosis
fibroids
vulvovaginitis
bacterial vaginosis
preterm birth
gestational age
birthweight
preeclampsia/eclampsia
gestational diabetes
infertility
use of artificial reproductive technologies
premature ovarian failure
nausea and vomiting of pregnancy
hyperemesis gravidarum
mode of delivery
spontaneous abortion
induced abortion
fetal anomalies
organ prolapse
postpartum hemorrhage
ovarian cyst
amenorrhea
dysmenorrhea
meningiorrhea
pelvic and perineal pain
pituitary tumor
Addison's disease
Cushing's syndrome
hyperthyroidism
hyperparathyroidism
Grave's disease
Hashimoto's Thyroiditis
age at menarche
age at menopause
age at first birth
parity
partner status
major depressive disorder
bipolar disorder
schizophrenia
postpartum depression
postpartum psychosis
PTSD
anxiety
OCD
metabolic syndrome
BMI
Type 1 diabetes
Type 2 diabetes
family psychiatric history
previous psychiatric history
early life adversity
loss of a family member
perinatal loss
baby time in NICU
sleep quality
socioeconomic status
parity
parity
